# MicroRNAs Play Key Roles in Progenitor Maintenance, Proliferation, and Osteogenic Differentiation of Osteogenic Progenitor Cells in Syndromic and Nonsyndromic Craniosynostosis

**DOI:** 10.3390/ijms27146140

**Published:** 2026-07-09

**Authors:** Akiko Suzuki, Chihiro Iwaya, Kamran Rahimi, Junichi Iwata

**Affiliations:** Department of Orthodontics and Pediatric Dentistry, School of Dentistry, University of Michigan, Ann Arbor, MI 48109, USA; akikosuz@umich.edu (A.S.); ciwaya@umich.edu (C.I.);

**Keywords:** microRNA, craniofacial anomalies, bioinformatics, bone biology, developmental biology, genetics

## Abstract

Craniosynostosis (CS) is a congenital birth defect defined by the premature closure of one or more cranial sutures. Approximately 70% of CS cases are nonsyndromic, with underlying causes frequently remaining unidentified. This study seeks to identify short noncoding RNAs, specifically microRNAs (miRNAs), associated with syndromic, nonsyndromic, and all forms of CS to advance understanding of its etiology. Through a systematic review, a total of 165 genes were identified as associated with human CS (112 syndromic, 37 nonsyndromic, and 13 overlapping). Bioinformatic analyses identified several miRNAs capable of regulating these CS-related genes. We found that miR-377 and miR-335-3p were specifically involved in the regulation of genes associated with syndromic CS, while miR-371-5p, miR-329, and miR-204-5p were specifically involved in gene regulation related to nonsyndromic CS. In contrast, miR-651-3p, miR-362-3p, and miR-425 play a role in both syndromic and nonsyndromic CS. Subsequent enrichment analysis using ShinyGO revealed that the predicted targets of these nine candidate miRNAs were preferentially enriched in the TGF-beta signaling pathway. Notably, functional modulation of these miRNAs altered the undifferentiated state, cell proliferation, and osteogenic differentiation of suture progenitor cells. Taken together, our study indicates that these miRNAs play a role in CS by changing the cell characteristics of suture progenitor cells.

## 1. Introduction

Craniosynostosis (CS) is a congenital birth defect resulting from the premature closure of one or more cranial sutures, primarily affecting the calvarial sutures. The occurrence of primary CS, a developmental condition characterized by the fusion of calvarial bones due to abnormal bone formation, is estimated to be about 1 in 2000 to 2500 live births worldwide [1,2]. Primary CS is classified into two categories: syndromic and nonsyndromic. Approximately 70% to 75% of cases are nonsyndromic, while the remaining 25% to 30% are syndromic [3]. Genetic predispositions are identified in approximately 20% of nonsyndromic CS cases, whereas the remaining 80% are attributed to a complex interplay of epigenetic and environmental factors [4]. Syndromic cases of craniosynostosis, which account for approximately 30% of all cases, are frequently attributed to pathogenic variants in specific genes, such as *FGFR2*, *FGFR3*, *EFNB1*, *ERF*, *SMAD6*, *TWIST1*, and *TCF1*.

The pathogenesis and clinical prognosis of congenital anomalies are profoundly shaped by diverse epigenetic modifications. In particular, microRNAs (miRNAs) orchestrate post-transcriptional gene silencing by targeting the 3′ untranslated regions (UTRs) of messenger RNAs, thereby inducing transcript degradation or translational repression [5]. Notably, gene expression is often subject to combinatorial regulation by multiple miRNAs, while individual miRNAs exhibit pleiotropic effects by modulating a broad spectrum of downstream targets. Consequently, dysregulation of miRNA expression can exert systemic effects on cellular homeostasis, often surpassing the impact of individual gene variants. Given this broad regulatory capacity, miRNAs have emerged as compelling therapeutic targets for a diverse range of pathologies [6]. However, it remains largely unknown whether and how miRNAs contribute to craniosynostosis in syndromic and nonsyndromic CS phenotypes.

## 2. Results

### 2.1. A Comprehensive Compendium of Human Craniosynostosis-Related Genes Identified via Systematic Literature Curation

**Figure 1:**
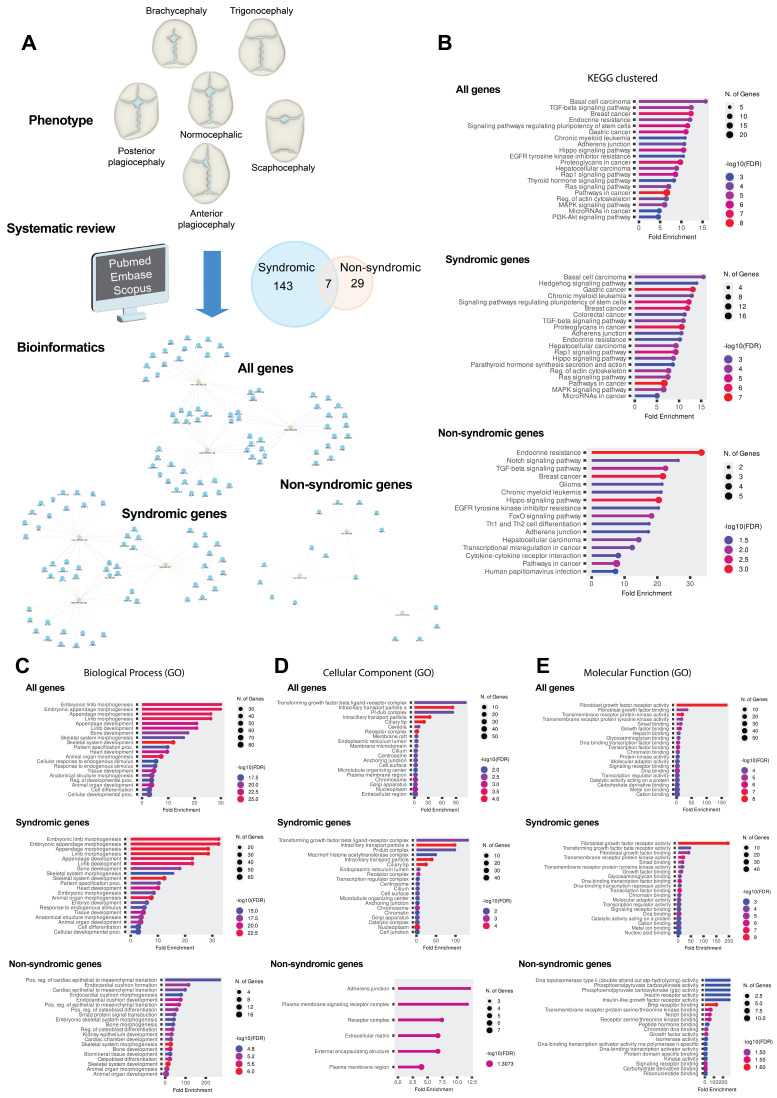
Identification of genes related to craniosynostosis and bioinformatic characterization of these genes. (**A**) Schematic summary of this study. (**B**) KEGG analysis for identified genes in all CS (all genes), syndromic CS (syndromic genes), and nonsyndromic CS types (non-syndromic genes). (**C**–**E**) GO analysis for biological processes (**C**), cellular component (**D**), and molecular function (**E**) for identified genes in all CS, syndromic CS, and nonsyndromic CS types.

Through the systematic review, we identified 8594 articles as of January 2026. After removing 2874 duplicates, we screened 5720 articles by title and abstract, excluding 4918 that did not meet our inclusion criteria. As a result, 714 articles underwent full-text review, resulting in the collection of 607 articles that provided information on genetic variants (442 articles) and structural variants (172 articles) associated with human craniosynostosis (Supplementary Figure S1). We included genes identified in patients with various types of craniosynostosis: trigonocephaly (due to metopic synostosis), scaphocephaly (due to sagittal synostosis), anterior plagiocephaly (due to unilateral coronal synostosis), posterior plagiocephaly (due to lambdoid synostosis), brachycephaly (due to bilateral coronal synostosis), cloverleaf skull (due to multiple suture synostosis), metopic synostosis, sagittal synostosis, coronal synostosis (both unilateral and bilateral), lambdoid synostosis, and pan synostosis. Through this systematic review, we identified a total of 165 gene variants and 178 structural variants (Supplementary Tables S2 and S3). Of these, 112 genes were associated with syndromic CS, 37 genes with nonsyndromic CS, and 13 genes overlapped between the syndromic and nonsyndromic CS groups (Figure 1A and Table 1).

Data from the Online Mendelian Inheritance in Man (OMIM) catalog revealed that both syndromic CS and all CS, including both syndromic and nonsyndromic CS, gene sets were associated with a wide range of systemic conditions. In contrast, nonsyndromic CS genes were predominantly linked to localized developmental anomalies (Supplementary Figure S2). Kyoto Encyclopedia of Genes and Genomes (KEGG) pathway analysis, which organizes genes by cellular functions and pathways, identified novel enrichment of CS genes related to hedgehog and transforming growth factor beta (TGFβ) signaling pathways and the pluripotency of stem cells in syndromic genes. In nonsyndromic CS, genes were enriched in endocrine resistance and Notch, TGFβ, and Hippo signaling pathways (Figure 1B; Supplementary Table S4). Thus, KEGG analysis demonstrated that major growth factor signaling pathways were significantly enriched across all CS classifications.

Gene Ontology (GO) analysis, which examines trends in biological processes, cellular components, and molecular functions, revealed significant enrichments in embryonic skeletal morphogenesis in both all CS and syndromic CS genes. In contrast, cardiac morphogenesis was enriched in nonsyndromic CS genes (Figure 1C; Supplementary Table S5). Interestingly, analysis of cellular components showed enrichment in the TGFβ ligand-receptor complex, primary cilium-associated factors, and the polycomb repressive deubiquitinase complex in all CS and syndromic CS, while adherens junction and extracellular matrix-associated factors were enriched in nonsyndromic CS genes (Figure 1D; Supplementary Table S6). Regarding molecular functions, significant enrichments were found in fibroblast growth factor (FGF) and TGFβ activity factors and transcription-associated factors in all CS and syndromic CS genes (Figure 1E; Supplementary Table S7). Interestingly, although one-third of the nonsyndromic CS genes overlapped with the syndromic CS gene set, the nonsyndromic CS gene set exhibited distinct results. These clustering analyses indicate genetic differences between syndromic and nonsyndromic CS. These findings underscore the importance of this work in deciphering the molecular mechanisms underlying calvarial development.

Next, we aimed to identify miRNAs that can regulate the expression of genes associated with CS. We analyzed CS genes separately, distinguishing between syndromic and nonsyndromic CS genes using 14 distinct databases. The top 10 miRNAs predicted from all CS genes were highly concordant with those of the syndromic group, but notably diverged from the nonsyndromic CS profile (Supplementary Tables S9–S11). For instance, several miRNAs, including miR-651-3p, miR-377, and miR-335-3p, overlapped between the total CS-related and the syndromic CS-specific gene sets. In contrast, only miR-362-3p was shared between the total CS and nonsyndromic gene sets. Notably, no miRNAs were found to be common to both the syndromic and nonsyndromic CS groups. These observations suggest that gene regulatory landscapes may diverge significantly between syndromic and nonsyndromic CS phenotypes.

**Figure 2:**
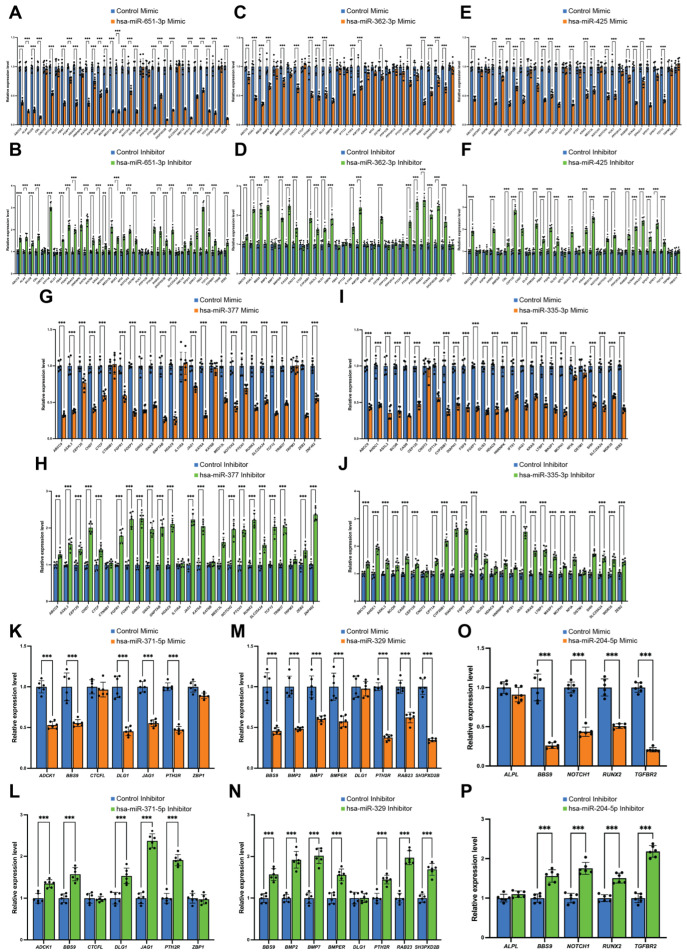
Identification of craniosynostosis-related genes regulated by candidate miRNAs. (**A**–**P**) Quantitative RT-PCR analysis of the indicated genes in cells treated with mimics or inhibitors for hsa-miR-651-3p (**A**,**B**), hsa-miR-362-3p (**C**,**D**), hsa-miR-425 (**E**,**F**), hsa-miR-377 (**G**,**H**), hsa-miR-355-3p (**I**,**J**), hsa-miR-371-5p (**K**,**L**), hsa-miR-329 (**M**,**N**), and hsa-miR-204-5p (**O**,**P**). Blue bars represent control samples; orange bars represent samples treated with the respective mimic; and light green bars represent samples treated with the respective inhibitor. * *p* < 0.05, ** *p* < 0.01, *** *p* < 0.001.

Next, to identify the genes targeted by each miRNA in suture mesenchymal cells, we analyzed the expression of CS-related genes in immortalized human cranial suture progenitor cells (iSuPs) treated with specific miRNA mimics and inhibitors (Figure 2). In all CS cases, the following genes were significantly downregulated by the mimic and upregulated by the inhibitor: miR-651-3p regulated the expression of a total of 26 CS-related genes (Figure 2A,B); miR-362-3p regulated 15 genes (Figure 2C,D); miR-425 regulated 25 genes (Figure 2E,F). In syndromic CS, miR-377 regulated 19 CS-related genes (Figure 2G,H) and miR-335-3p regulated 24 genes (Figure 2I,J). In nonsyndromic CS, miR-371-5p regulated 5 genes (Figure 2K,L); miR-329 regulated 7 genes (Figure 2M,N); miR-204-5p regulated 4 genes (Figure 2O,P). While no overlapping CS-related genes were identified as miRNA-targets across syndromic and nonsyndromic cases, three genes—*ABCC9*, *HDAC9*, and *ZEB2*—emerged as convergent targets of miRNAs associated with syndromic CS. In contrast, *BBS9* was uniquely identified as a frequent target of miRNAs related to nonsyndromic CS.

To understand the functional networks regulated by these 9 miRNAs, we conducted a pathway enrichment analysis. This analysis tested whether the predicted target genes were overrepresented within specific functional clusters of the initial 165 CS-associated gene set. The analysis showed that these target genes were preferentially enriched in the TGFβ signaling pathway. This result suggests that the identified candidate miRNAs primarily converge on a key signaling pathway associated with calvarial suture patency.

### 2.2. Overexpression of CS-Related miRNAs Alters Progenitor Marker Expression and Inhibits Cell Proliferation and Osteogenesis in Human Suture Mesenchymal Cells

Bioinformatic analyses indicated that CS-related genes were enriched in processes related to progenitor maintenance and bone formation. These results suggest that the predicted miRNAs may interfere with multiple cellular processes, including the maintenance of the undifferentiated state, cell proliferation, apoptosis, and osteogenic differentiation of suture mesenchymal cells. The top three predicted miRNAs from each CS category were selected for further analysis: hsa-miR-651-3p, hsa-miR-362-3p, and hsa-miR-425 for all CS cases; hsa-miR-651-3p, hsa-miR-377, and hsa-miR-335-3p for syndromic CS; and hsa-miR-371-5p, hsa-miR-329, and hsa-miR-204-5p for nonsyndromic CS.

**Figure 3:**
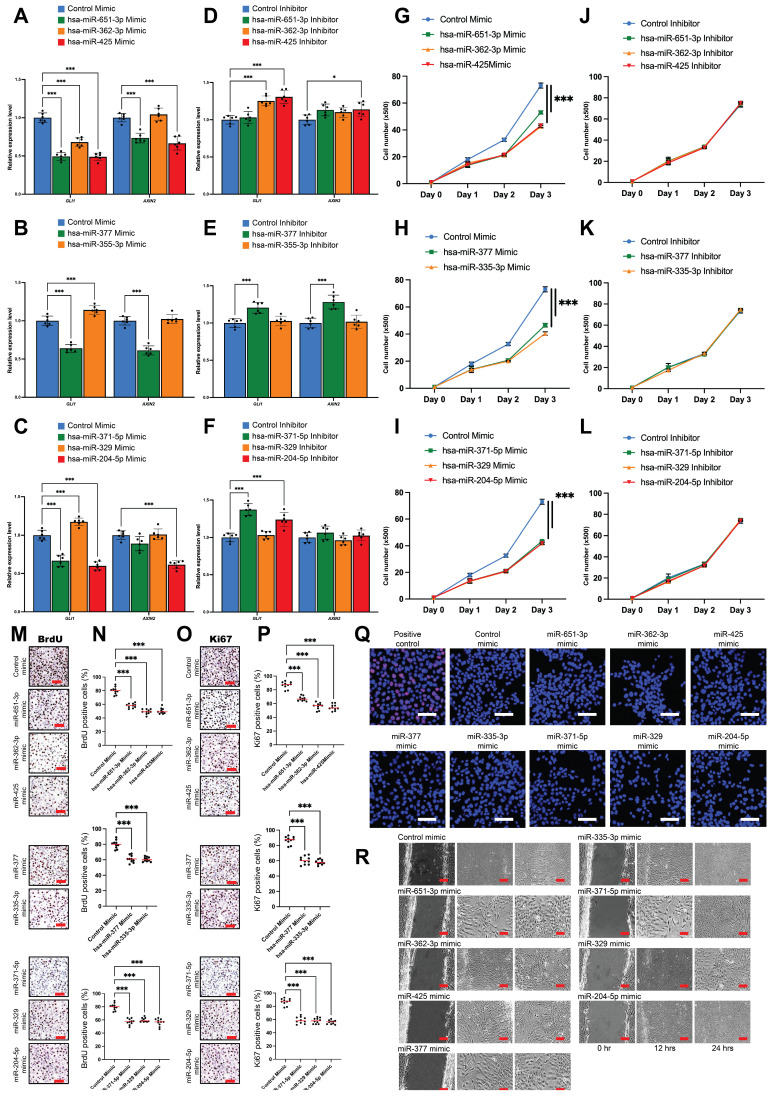
Effects of candidate miRNAs on progenitor maintenance, proliferation, death, and migration. (**A**–**F**) Quantitative RT-PCR analysis of *GLI1* and *AXIN2* expression in cells treated with specific miRNA mimics (**A**–**C**) and inhibitors (**D**–**F**). Panels (**A**,**D**) correspond to all CS types; panels (**B**,**E**) correspond to syndromic CS type; panels (**C**,**F**) correspond to nonsyndromic type. * *p* < 0.05, *** *p* < 0.001. (**G**–**L**) Cell proliferation assays over three days with cells treated with the indicated miRNA mimics (**G**–**I**) and inhibitors (**J**–**L**). *** *p* < 0.001. (**M**–**P**) BrdU incorporation assays (**M**,**N**) and Ki67 immunocytochemical analysis (**O**,**P**) with predicted miRNA mimics in human suture mesenchymal stem cells. Scale bars: 50 µm. ***: *p* < 0.001. (**Q**) TUNEL assays with predicted miRNA mimics in human suture mesenchymal stem cells. Scale bars: 50 µm. (**R**) Scratch assays with predicted miRNA mimics in human suture mesenchymal stem cells. Scale bars: 50 µm.

The impact of miRNA overexpression on the undifferentiated state of these cells was assessed by measuring the expression of suture progenitor markers *Gli1* and *Axin2* [7,8]. Ectopic expression of these miRNAs significantly downregulated both markers (Figure 3A–F). Cell proliferation and cell death assays were performed to evaluate cell survival. Overexpression of the selected miRNAs using specific mimics significantly reduced cell proliferation, whereas miRNA inhibitor treatment had no effect (Figure 3G–L). The suppression of proliferation by these miRNA mimics was further confirmed by BrdU incorporation assays (Figure 3M,N) and Ki-67 immunocytochemistry (Figure 3O,P). Overexpression of these miRNAs did not induce cell death (Figure 3Q). In addition, cell migration was unaffected by miRNA overexpression (Figure 3R).

**Figure 4:**
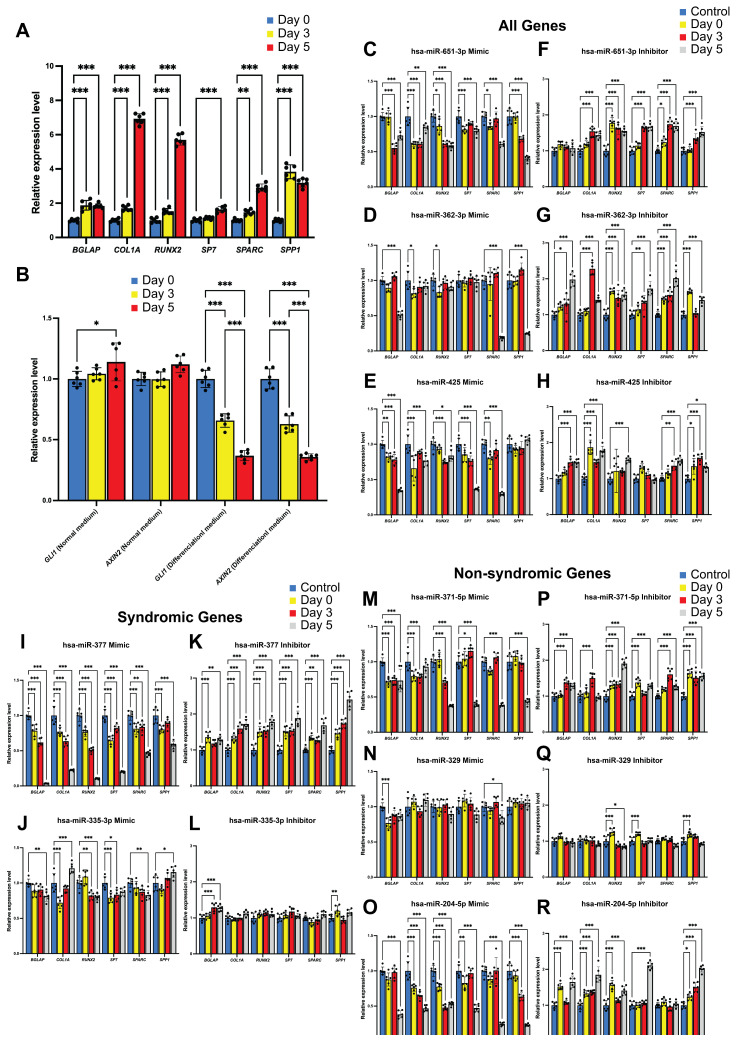
Effects of candidate miRNAs on osteogenic differentiation. (**A**) Quantitative RT-PCR analysis of the indicated osteogenic differentiation-related genes in cells induced osteogenic differentiation at Days 0, 3, and 5. ** *p* < 0.01, *** *p* < 0.001. (**B**) Effect of osteogenic induction on *GLI1* and *AXIN2* expression. * *p* < 0.05, *** *p* < 0.001. (**C**–**R**) Quantitative RT-PCR analysis of osteogenic differentiation-related genes in cells treated with either the indicated mimic (**C**–**E**,**I**,**J**,**M**–**O**) or inhibitor (**F**–**H**,**K**,**L**,**P**–**R**) in all craniosynostosis genes (**C**–**H**), syndromic craniosynostosis genes (**I**–**L**), and nonsyndromic craniosynostosis genes (**M**–**R**). * *p* < 0.05, ** *p* < 0.01, *** *p* < 0.001.

Next, we investigated whether overexpression of predicted miRNAs dysregulates osteogenic differentiation. We found that within three days of osteogenic induction, iSuPs expressed osteogenic differentiation genes (Figure 4A,B). Overexpression of miR-651-3p, miR-377, miR-371-5p, and miR-204-5p resulted in a marked downregulation of all osteogenic marker gene expression, whereas inhibition of these miRNAs led to upregulation in all CS genes (Figure 4C–H), syndromic CS genes (Figure 4I–L), and nonsyndromic CS genes (Figure 4M–R). Interestingly, miR-362-3p and miR-329 did not appear to influence osteogenic differentiation.

Collectively, these findings indicate that ectopic expression of these miRNAs diminishes the progenitor characteristics of suture mesenchymal cells and suppresses cell proliferation and osteogenic differentiation, potentially impairing the maintenance of long-term suture width.

## 3. Discussion

While the genetic basis of CS is predominantly documented in syndromic cases (involving 112 genes), 37 genes have been implicated in nonsyndromic (isolated) forms, with 13 genes overlapping between both categories. Our bioinformatic analysis of upstream miRNAs suggests that the mechanisms underlying syndromic and nonsyndromic CS may differ. Although calvarial suture mesenchymal progenitor cells possess undifferentiated characteristics that parallel those of mesenchymal cells in other organs, they also possess unique traits related to bone development as well as bone injury repair and regeneration [9,10]. This indicates that certain epigenetic factors, including miRNAs, could play a role in regulating these progenitor cells.

A recent study shows that miRNAs are involved in the regulation of osteoblast, osteoclast, and chondrocyte function [11]. The expression levels of miR-425-5p, miR-362-3p, and miR-335-3p are significantly reduced in pre-osteoblasts, and miR-335-3p is downregulated in mature osteoblasts during osteogenic induction in human amniotic stem cells [12]. Furthermore, miR-335 expression is diminished in human bone marrow stem cells (hBMSCs) during osteogenic differentiation [13], and overexpression of miR-335 in hBMSCs suppresses cell proliferation and promotes osteogenic differentiation [14,15]. Interestingly, transgenic mice with miR-335-5p overexpression driven by the *Osterix* promoter exhibit increased trabecular bone formation in the distal femur metaphysis, which is attributed to enhanced osteoblast proliferation and differentiation [16]. In this study, we demonstrated that treatment with a miR-335-3p inhibitor specifically upregulates *BGLAP* expression, a gene involved in osteoblast maturation, without inducing other early osteogenic genes. These findings indicate that miR-335-3p is critical for osteoblast maturation.

Overexpression of miR-204-5p inhibits osteogenic differentiation in human adipose-derived stem cells [17]. Interestingly, miR-204-5p levels are significantly higher in skin fibroblasts from patients with osteogenesis imperfecta compared to healthy controls [18], and miR-204-5p inhibits BMP2-induced osteogenic differentiation in ligament fibroblasts from patients with ankylosing spondylitis [19]. In this study, we demonstrated that overexpression of miR-204-5p suppresses *RUNX2*, *ALPL*, and *NOTCH1*, which are essential for osteogenesis, thereby inhibiting osteogenic differentiation.

Maintenance of calvarial suture width depends on the preservation of stemness in suture mesenchymal cells [7,8]. Numerous studies on miRNA-mediated stemness maintenance indicate that these cells express tissue- and condition-specific miRNAs. Dysregulation of these miRNA expression patterns disrupts stemness and promotes differentiation. Indeed, overexpression of miR-329 in human colorectal cancer cells inhibits tumorigenesis and stemness [20]. This suggests that miR-329 may regulate cell fate and proliferation in sutures. In this study, we confirmed that miR-329 regulates both the undifferentiated state and proliferation in suture osteogenic progenitor cells. Notably, while miR-329 and miR-362-3p suppressed proliferation and downregulated undifferentiated markers, they did not alter osteogenic differentiation. This distinct phenotype suggests that these specific miRNAs are primary regulators of the uncommitted suture progenitor pool size, triggering cell cycle exit without automatically initiating downstream matrix mineralization signaling. Our sub-cluster enrichment further revealed that the predicted targets of the 9 candidate miRNAs preferentially converge on the TGFβ signaling pathway. Given that dysregulated TGFβ signaling is a well-established hallmark of suture pathologies, these candidate miRNAs may serve as critical epigenetic tuners governing the fine balance of TGFβ ligands and receptors during calvarial morphogenesis.

We envision utilizing these distinct convergent targets in two main clinical areas: (1) Differential Diagnosis (Biomarkers): Since clinical presentations can sometimes overlap, analyzing the expression profiles of these specific candidate miRNAs in patient serum or localized suture biopsies could serve as a molecular diagnostic toolkit. This would allow clinicians to differentiate between syndromic and nonsyndromic forms at an early stage, especially in cases where standard genetic sequencing yields variants of uncertain significance (VUS). (2) Targeted Therapeutics (Personalized Medicine): For therapeutics, these distinct axes suggest that a “one-size-fits-all” approach will not work. Instead, stratified or personalized microRNA-based therapies could be developed. For example, localized delivery (e.g., via specialized hydrogels or nanoparticle injections into the affected suture) of specific miRNA mimics or antagomirs could be used to selectively normalize the dysregulated expression of *ZEB2* in syndromic patients or *BBS9* in nonsyndromic patients, thereby preventing premature suture fusion without causing systemic off-target effects.

In summary, to date, the biological functions of miR-651-3p, miR-362-3p, and miR-425/miR-425-5p in development remain largely unknown. Our study suggests that these specific miRNAs may contribute to CS by regulating the undifferentiated state of suture progenitor cells, offering potential targets for miRNA expression manipulation in CS tissue. Future studies to validate these specific miRNA-mRNA axes in primary tissues and cells from patients will prioritize a direction for the next phase of research.

## 4. Materials and Methods

### 4.1. Systematic Review

The literature search, conducted according to the PRISMA guidelines, included original articles or case reports in English that described genes related to craniosynostosis using the search string: craniosynostosis, human gene, and English. A literature search was conducted in January 2026 through three major literature databases: PubMed (National Library of Medicine), Embase (Ovid), and Scopus (Elsevier). The search was specifically designed to identify literature on craniosynostosis, genetics, and gene variants in any species. A combination of Medical Subject Headings (MeSH) terms, titles, and abstracts was developed for the initial PubMed search and then adapted for the other databases. Articles were excluded when they reported no gene variants or structural variants, focused on secondary craniosynostosis, provided only in vitro results, or fell into categories such as review articles, editorials, conference proceedings, or comments. Any articles not meeting these criteria were also excluded. The citations retrieved were stored in the reliable online application for systematic reviews, Rayyan (https://www.rayyan.ai/, 24 April 2026).

### 4.2. Bioinformatic Analysis

miRNAs were predicted using a miRNA-target interaction *R* package (version 4.5.3; https://cran.r-project.org/, 24 April 2026) and an associated database containing 50 million records from 14 distinct sources [21]. The enrichment significance of the miRNAs was assessed using Fisher’s exact test. The miRNA-gene network was visualized using an *R* package specific to human craniosynostosis. To cluster the candidate genes identified in the literature search, KEGG pathway and GO enrichment analyses were conducted using ShinyGO 0.85.1 (http://bioinformatics.sdstate.edu/go/, 30 June 2026). Categories were filtered for significance with a false discovery rate (FDR)-adjusted *p*-value of less than 0.05 and at least four genes related to hypertelorism. The *p*-value was calculated using a hypergeometric test, and a hierarchical level of 4 was established as a cutoff to avoid overly general GO terms.

### 4.3. Suture Mesenchymal Cell Culture

iSuPs, a mesenchymal cell line derived from human cranial suture (T0697, Applied Biological Materials Inc., Richmond, British Columbia, Canada), were maintained in PriGrow III medium (TM003, Applied Biological Materials Inc., Richmond, British Columbia, Canada). The medium was supplemented with 10% non-heat-inactivated fetal bovine serum (FBS) and 1% penicillin/streptomycin solution (P4333, Sigma-Aldrich, Darmstadt, Germany). Cells were cultured at 37 °C in a 5% CO_2_ atmosphere.

### 4.4. Quantitative RT-PCR

iSuPs were plated in a 60 mm culture dish. Once the cells reached 80% confluence, they were treated with either mimic or inhibitor for has-miR-651-3p, has-miR-362-3, has-miR-425, has-miR-377, has-miR-335-3, has-miR-371-5, has-miR-329, and has-miR-204-5p, along with a negative control (n = 6 per group). After 24 h of treatment, total RNA was extracted, and the mRNA levels of the target genes were measured by quantitative RT-PCR, as previously described [22]. The primers used for qRT-PCR are listed in Supplementary Table S1.

### 4.5. Cell Proliferation Assay

iSuPs were seeded into 96-well cell culture plates at a density of 1000 cells per well. After 6 h, the cells were transfected with either mimic for negative control (4464061, Thermo Fisher Scientific, Waltham, MA, USA) or various miRNA mimics (has-miR-651-3p, has-miR-362-3, has-miR-425, has-miR-377, has-miR-335-3, has-miR-371-5, has-miR-329, and has-miR-204-5p) using Lipofectamine RNAiMAX (Thermo Fisher Scientific, Waltham, MA, USA) according to the manufacturers’ protocol. Similarly, inhibitors for the negative control and the mentioned miRNAs were also applied (4464079, Thermo Fisher Scientific, Waltham, MA, USA). Cell proliferation was assessed using the Cell Counting Kit-8 (Dojindo Molecular Technologies, Rockville, MD, USA) at 24, 48, and 72 h post-transfection (n = 6 per group).

### 4.6. Osteogenic Induction Assay

iSuPs were seeded into 12-well culture plates and maintained until 100% confluence was achieved. Osteogenic induction was initiated by switching to osteogenic induction medium (MEM-alpha supplemented with 100 mg/mL of ascorbic acid (A4544, Sigma-Aldrich), 5 mmol/L beta-glycerophosphate (G9422, Sigma-Aldrich, Darmstadt, Germany), 10 nmol/L dexamethasone (D4902, Sigma-Aldrich, Darmstadt, Germany), 10% FBS, penicillin/streptomycin, and L-glutamine), and 100 ng/mL human recombinant BMP2 (355-BM, R&D Systems, Minneapolis, MN, USA) for up to 7 days. The osteogenic induction medium was refreshed every other day. The expression of osteogenic differentiation marker genes was analyzed by quantitative RT-PCR at Day 7 post-induction (n = 6 per group).

### 4.7. Cell Migration Assay

iSuPs cells were seeded in 12-well culture plates. Upon reaching 70% confluence, cells were transfected with either an individual miRNA mimic or a negative control. After 24 h, a straight scratch was introduced at the center of the cell layer using a P100 pipette tip. Cell migration was assessed at 0, 12, and 24 h post-scratch, and images were captured using a phase-contrast microscope (CKY, Olympus, Tokyo, Japan) (n = 3 per treatment).

### 4.8. Statistical Analysis

Statistical analyses were performed using Prism software ver.11 (GraphPad Software, San Diego, CA, USA). For comparisons between two groups, a two-tailed Student’s *t*-test was employed. Multiple group comparisons were conducted using a one-way analysis of variance (ANOVA) followed by the Tukey–Kramer post hoc test. Cell proliferation data were evaluated using a two-way ANOVA. All quantitative results are representative of at least three independent experiments and are presented as mean ± standard deviation (SD). A *p*-value of <0.05 was considered statistically significant.

## Figures and Tables

**Table 1:** Genes associated with syndromic and nonsyndromic CS.

	Genes
Syndromic (112 genes)	*ABCC9*, *ACTB*, *ADAMTSL4*, *AHDC1*, *ANTXR1*, *ASPM*, *ASXL1*, *ASXL3*, *AXIN2*, *B3GAT3*, *BCOR*, *CASR*, *CDC45*, *CETP*, *CHD7*, *CHST3*, *CNOT2*, *COLEC10*, *COLEC11*, *CPT1A*, *CRTAP*, *CTCF*, *CTNNB1*, *CTSK*, *CYP17A1*, *CYP21A2*, *CYP26B1*, *DDX3X*, *DIAPH1*, *DIS3L2*, *DMPK*, *DPH1*, *EFNA4*, *EFNB1*, *EFTUD2*, *ERCC2*, *ERF*, *ESCO2*, *FAM20C*, *FBN1*, *FGF9*, *FGFR4*, *FGFRL1*, *FLNA*, *FOXP1*, *FTO*, *GINS2*, *GLI3*, *GLIS3*, *GNPTAB*, *GPC3*, *HDAC9*, *HNRNPK*, *HUWE1*, *IDS*, *IFT122*, *IFT140*, *IFT81*, *IL11RA*, *KAT6A*, *KAT6B*, *KMT2D*, *KRAS*, *LHX3*, *LRP5*, *LTBP1*, *MAGEL2*, *MAP3K20*, *MAP4K4*, *MASP1*, *MBTPS1*, *MCPH1*, *MED13L*, *MEGF8*, *MEIS2*, *MYADML2*, *NFIA*, *OSGEP*, *OSTM1*, *P4HB*, *POR*, *PPP2R1A*, *PTCH1*, *PTPN11*, *PTPRD*, *RAB5IF*, *RECQL4*, *RNU12*, *SCN4A*, *SHH*, *SHOC2*, *SKI*, *SLC25A24*, *SMAD3*, *SMAD6*, *SMC1A*, *SPAG17*, *SPRY1*, *TBX3*, *TCOF1*, *TCTN3*, *TEDC1*, *TGFBR1*, *TGFBR3*, *TRAF7*, *TRIM37*, *TRPM3*, *WDR19*, *WDR35*, *ZEB2*, *ZIC1*, *ZNF462*
Nonsyndromic (37 genes)	*ADCK1*, *ALPL*, *AXIN1*, *BBS9*, *BMP2*, *BMP7*, *BMPER*, *CASS4*, *CEP135*, *CIMIP1*, *CTCFL*, *DLG1*, *DLX6-AS1*, *FAM209A*, *FGF23*, *FREM1*, *HSD3B7*, *IGF1R*, *ITPA*, *NELL1*, *NOTCH1*, *PCCA*, *PCK1*, *PDILT*, *PLEKHA6*, *PPP1CB*, *PTH2R*, *RBM38*, *RTF2*, *SDHAF3*, *SEM1*, *SH3PXD2B*, *SHC4*, *SPO11*, *TRPV4*, *TSHR*, *ZBP1*
Overlapped between Syndromic and Nonsyndromic (13 genes)	*ALX4*, *FGFR1*, *FGFR2*, *FGFR3*, *GNAS*, *JAG1*, *MSX2*, *NOTCH2*, *RAB23*, *RUNX2*, *TCF12*, *TGFBR2*, *TWIST1*
Unclear (3 genes)	*CBL*, *HADHA*, *MCM5*

## Data Availability

All data from this study are available in the Supplemental Information.

## Supplementary Materials

The following supporting information can be downloaded at: https://www.mdpi.com/article/10.3390/ijms27146140/s1.
