# Supplementary material for: MicroRNAs Play Key Roles in Progenitor Maintenance, Proliferation, and Osteogenic Differentiation of Osteogenic Progenitor Cells in Syndromic and Nonsyndromic Craniosynostosis
Source: Int J Mol Sci. 2026 Jul 9;27(14):6140. doi: 10.3390/ijms27146140 (PMC13410375; doi:10.3390/ijms27146140)
Supplement: Supplementary file 1 [file ijms-27-06140-s001.zip › ijms-4427830-supplementary.pdf]

Supplementary Information

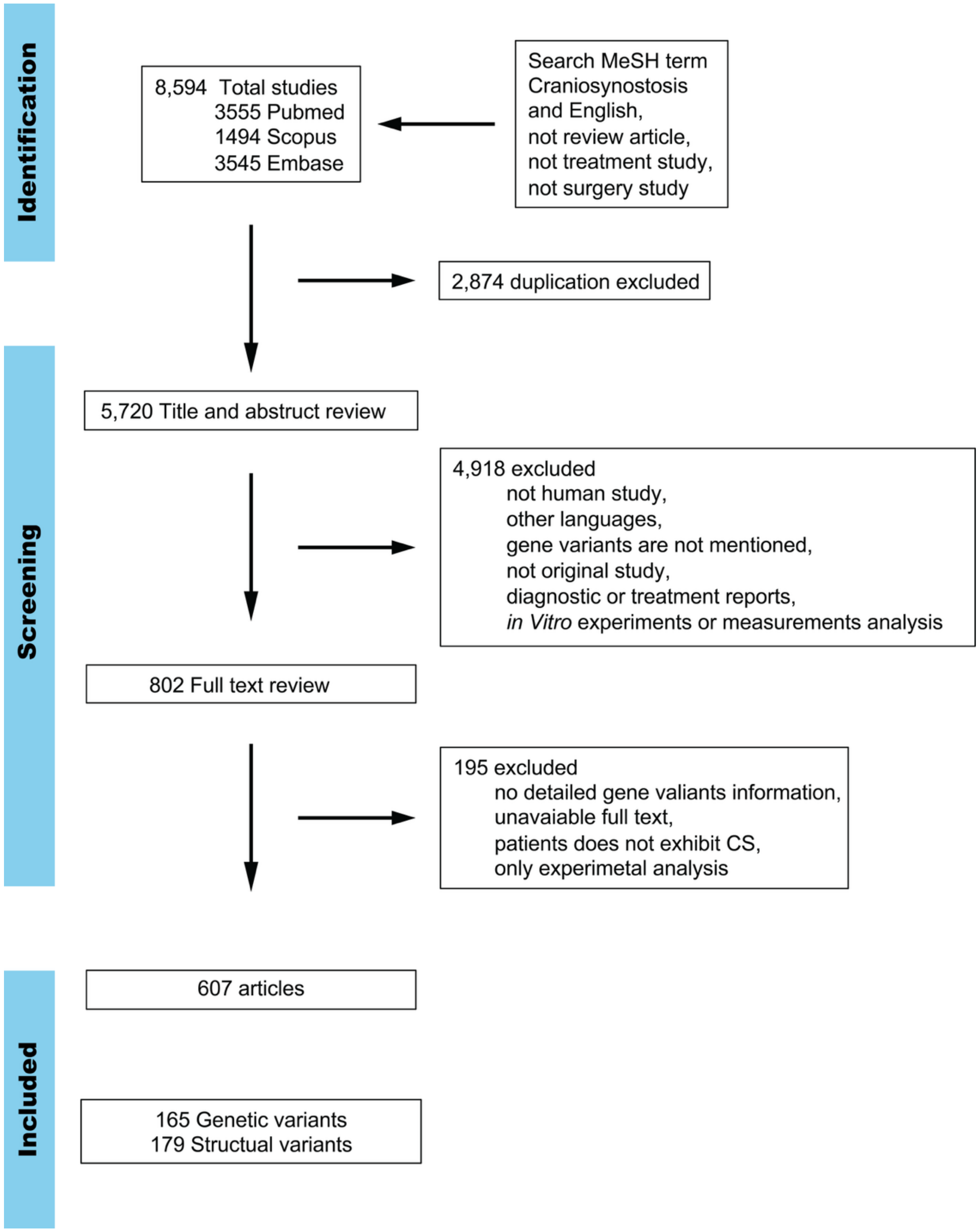

Figure S1. PRISMA flow chart.

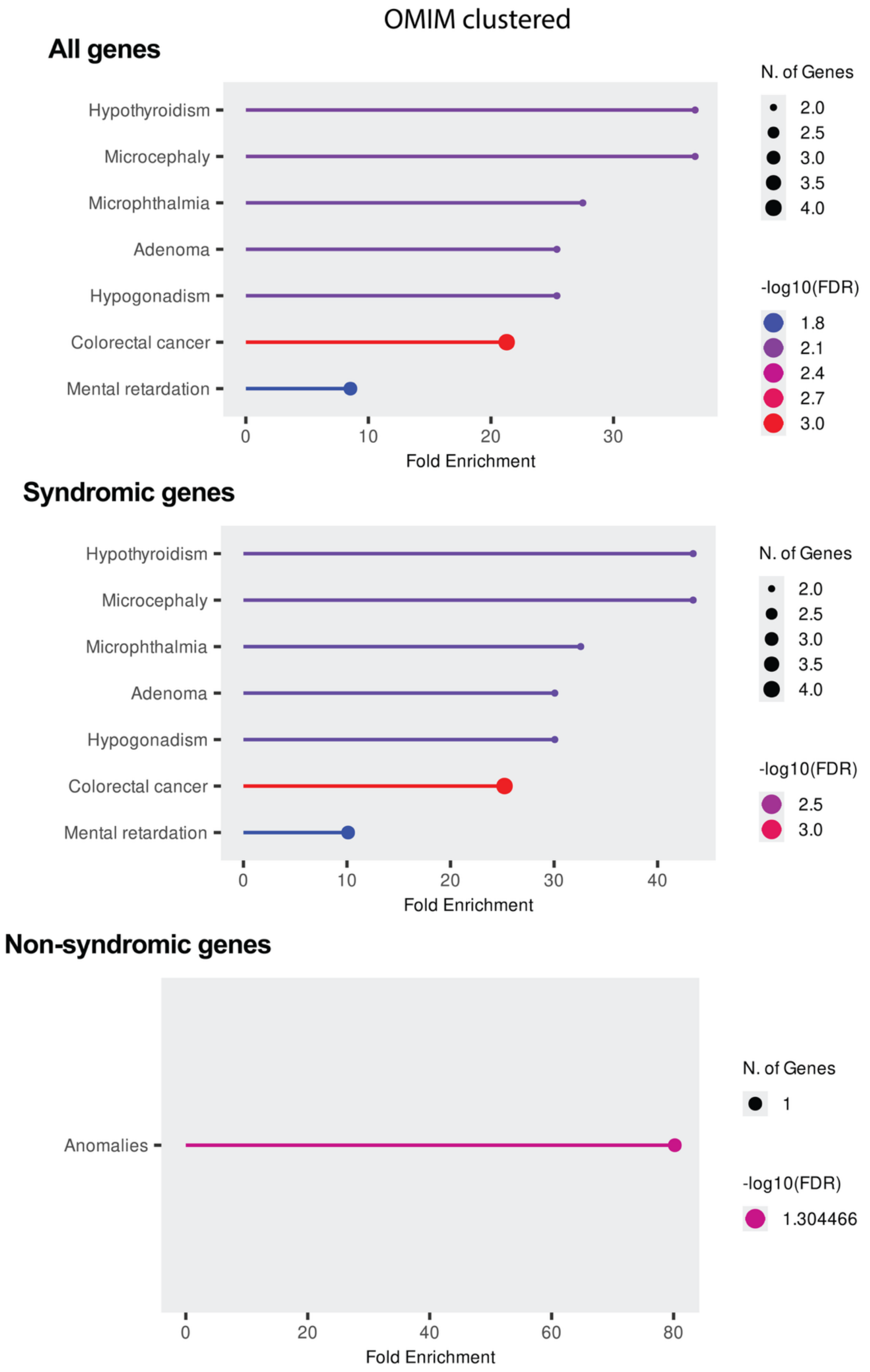

Figure S2. OMIN clustering for identified genes in all CS, syndromic CS, and nonsyndromic CS types.

Table S1. Primers used in this study.

Gene symbol	Forward primer sequence	Reverse primer sequence
ABCC9	ATCCTGGTCACACCACTGTTCC	AGTTCGCCAACTGTCGTCACCA
ADCK1	GACTCGGTCAACAGAGGCATCA	GTGGTTGAGGAGATGGCTGATC
AHDC1	GACCTTCTCTGAGTCATCCTCC	GCAGATGATGCCTCGTTCCAGT
ALPL	GCTGTAAGGACATCGCCTACCA	CCTGGCTTTCTCGTCACTCTCA
ALX4	TGAGGACAGACCTCACTGAGGC	GGCAGCTCATATGCAGTGGAGA
ANTXR1	GAATGACGGCTTTCAGGCTCTG	GAAGCCGTTTCCTCTCACGACA
ASPM	GAGACCTTGGTGGAATACCTGC	ACGAAGATCCAAAAGCCTTGCAC
ASXL1	CGGCTTGAAGATCGTCAGTCCT	GGCTGACCTTTAACCACCCAGG
ASXL3	GAAAGAACAGCCTCCCAGAGAG	CTGACAGATGTGCTAGTGGATGC
AXIN2	CAAACTTTCGCCAACCGTGGTTG	GGTGCAAAGACATAGCCAGAACC
BBS9	GCTTAGTGCTGACCAGGTTGCT	AAATGGCGGCATCCACCGTTTC
BCOR	TGTCTACCCGCTGCTTACTGTG	TCTCGGAGTCTTTGGTTGCTGG
BGLAP	CGCTACCTGTATCAATGGCTGG	CTCCTGAAAGCCGATGTGGTCA
BMP2	TGTATCGCAGGCACTCAGGTCA	CCACTCGTTTCTGGTAGTTCTTC
BMP7	GAGTGTGCCTTCCCTCTGAACT	AGGACGGAGATGGCATTGAGCT
BMPER	GTGAAGAGTGCCTCCTACGAGT	GTCCTGCCTTTCACACAAGCAC
CASR	GCTCTTCACCAATGGCTCCTGT	CCACACTCATCAAAGGTCACCTG
CASS4	GACTCTGCCTTCCCAGGTGTAT	GGATGGAGTCCTGCCTTCTTGG
CBL	GCACGTTCAGTCTGGATACCTC	GCAGTTTTGGCACAGGAAGAGG
CEP135	GCAAGATGACCTGGCTACAATGG	TCTCCCATCGTGACACCTCTGT
CHD7	CCTTTCCATGCTGAAGTTCCTGC	TCAGGCATACCGACTCGTTCCA
CNOT2	CTGGGCATCTTCACCTTGTCGA	GAGAAGGTCTTCACCATATCGGC
COL1A1	GATTCCCTGGACCTAAAGGTGC	AGCCTCTCCATCTTTGCCAGCA
CPT1A	GATCCTGGACAATACCTCGGAG	CTCCACAGCATCAAGAGACTGC
CTCF	GACCACACAAGTGCCATCTCTG	ATGTCGCAGTCTGGGCACTTGT
CTCFL	AGTGAGAAGCCTCACCTGTGTC	GACGAGTTCTCCACTGGTGACA
CTNNB1	CACAAGCAGAGTGCTGAAGGTG	GATTCCTGAGAGTCCAAAGACAG
CYP26B1	GCACCTCTTTGAGGTCTACCAG	AGGATCTGCCGAGCCTGAATGC
DIAPH1	ATCCTGGAGGTGAATGAGGCTG	ACCACGCCAAACTGCTCTGACT
DIS3L2	CATGTCCGACAAGCTGACCTTC	CGCAGGGATTTTCTCAGTTGGG
DLG1	GTGAAAAGGCGGAAGCCAGCAT	GTGCTGATTTCCAACACCTCCAG
DMPK	CACCGACACATGCAACTTCGAC	AGTAGCCCACAAAAGGCAGGTG
FAM20C	CTGACTACTGCGAGGAGGTGAA	TCTCGTAGTGGTGACGGTCCAT
FBN1	GGATACACAGGTGATGGCTTCAC	GTCGCATTCACAGCGGTATCCT
FGF9	CCAGGAAAGACCACAGCCGATT	CCATACAGCTCCCCCTTCTCAT
FGFR1	GCACATCCAGTGGCTAAAGCAC	AGCACCTCCATCTCTTTGTCGG
FOXP1	CAAAGAACGCCTGCAAGCCATG	GGAGTATGAGGTAAGCTCTGTGG
GAPDH	GTCTCCTCTGACTTCAACAGCG	ACCACCCTGTTGCTGTAGCCAA
GINS2	AGCCAAACTCCGAGTGTCTGCT	CTTGTGTGAGGAAAGTCCCGCT
GLI1	AGCCTTCAGCAATGCCAGTGAC	GTCAGGACCATGCACTGTCTTG
GLIS3	AAGCCAGGTCTCTACAGCATGC	ACTCAAGGTCGTGGACGCCAAA
GNAS	GCAGACAGATGCGCAAAGAAGC	GCTTTTACCAGATTCTCCAGCAC
GNPTAB	ATTCAGCCTGCGATTGGGATGG	TATTCCTCCACCAAACTGCCAGG
GPC3	CATTGGAGGCTCTGGTGATGGA	TTGTCCTTCGGAGTTGCCTGCT
HDAC9	TCTCGTCTCCAGGACTCACTCT	GCACTGGTGTTTCAGCATCAAGG
HNRNPK	GCAGATGGCTTATGAACCACAGG	AATCCGCTGACCACCTTTGCCA
IFT122	GTCTTCTCCATTTCTGCCGTGG	CACGCCAATCAGTGTCTGTGAC
IFT81	AGTTTCAGAGCCAGCTATGGGC	GTGAGAGTTTGCCTTCAATGGGC
IL11RA	GCTGTGTTGTCCTGGAGTGACT	ATGTAGGTGCCCTCATCAGTGC
JAG1	TGCTACAACCGTGCCAGTGACT	TCAGGTGTGTCGTTGGAAGCCA
KAT6A	CTTCAGTGAGAGCAGCGAGGAG	GTGGTGTTTGCGCTTTCGGACT
KAT6B	GGCAAGGATTTGGACGGTTTCTC	CGCTCTTCCAATATGCCAGGTAG
KMT2D	GGAATGGGTAGCTCTTTGGCGA	TGCCGAATCAGCAGCTCTCGTA
KRAS	CAGTAGACACAAAACAGGCTCAG	TGTCGGATCTCCCTCACCAATG
LTBP1	TGAATGCCAGCACCGTCATCTC	CTGGCAAACACTCTTGTCCTCC
MASP1	CCAATGAGGAGCGTTTCACAGG	AGGAGCAGTAGTAGCCGCCAAT
MCPH1	GTGACGGCTTTAAGGACCTCATC	CAACCTGGATGACGACATTCTGC
MED13L	GAAGTCCCAAGCCCGAGGAAAT	CTCGGCAACATCTTCAGTGGAG
MSX2	CGGAAAATTCAGAAGATGGAGCG	CGGCTTCCGATTGGTCTTGTGT
NFIA	GTGGAGGATGAAATGGACAGTCC	CTGCTGAAACCAGACTTCTCCG
NOTCH1	GGTGAACTGCTCTGAGGAGATC	GGATTGCAGTCGTCCACGTTGA
NOTCH2	GTGCCTATGTCCATCTGGATGG	AGACACCTGAGTGCTGGCACAA
OSTM1	CAGGTCGTCAGCAAGATGGACA	GCACAATTTGCCTCCTGCCATG
PCK1	CATTGCCTGGATGAAGTTTGACG	GGGTTGGTCTTCACTGAAGTCC
PPP1CB	GAGGAAACCATGAGTGTGCTAGC	CCACAATGGCTGCTATAGGCAG
PPP2R1A	ACCGCATGACTACGCTCTTCTG	TTGAAGCGGACATTGGCAACCG
PTCH1	GCTGCACTACTTCAGAGACTGG	CACCAGGAGTTTGTAGGCAAGG
PTH2R	CCATTGAGGCAACTTCTGTGGAC	AGGTAGAGACCTTCCACCAGGA
PTPRD	TAGTCCCAGTGTCCAGTTCAGG	CCAAGACAGCAACACGGAAGTC
RAB23	GTAGCCGAAGTGGGAGATATACC	ACCTTTTTGCCAGTGCCTCAGC
RAB5IF	AGTCTCCGTCTGGAGTAAGGTG	CCTGCTATTCCCAAGAACCCTC
RUNX2	CCCAGTATGAGAGTAGGTGTCC	GGGTAAGACTGGTCATAGGACC
SCN4A	GCCTGCTTCAAGATTGTCGAGC	TAGGATGGTGCGAATGACTCGC

SH3PXD2B	CACCAAAGATGAGGCAGAGACC	TGATGCCGTCTGGGATGGTTGT
SHH	CCGAGCGATTTAAGGAACTCACC	AGCGTTCAACTTGTCCTTACACC
SKI	CCTTCCGAAAAGGACAAGCCGT	GCTCTTTCTCACTCGCTGACAC
SLC25A24	GGCTTTGGTGAGAACTCGCATG	GGTGATGCCTCTGTAAAGTCCTG
SMC1A	CCTGAGACCTTCTTGCCTCTTG	GAGGTGGCTCATAGCGAATCAC
SP7	TTCTGCGGCAAGAGGTTCACTC	GTGTTTGCTCAGGTGGTCGCTT
SPAG17	CCAGAGCCTAAATGTGTCTTGCC	GGTAGCTCTGACGGACCATGAT
SPARC	TGCCTGATGAGACAGAGGTGGT	CTTCGGTTTCCTCTGCACCATC
SPO11	CTTTGGTAACCAGACTGTCGTCG	CACTTTGGTGCCATCTTCCTCG
SPP1	CGAGGTGATAGTGTGGTTTATGG	GCACCATTCAACTCCTCGCTTTC
SPRY1	GAAAGAGGACCTGACACAGCAC	CTCTCAGCAGAGCAAAGGCACT
TBX3	GGACACTGGAAATGGCCGAAGA	GCTGCTTGTTCACTGGAGGACT
TCF12	CCAGCAGTTCACCTTACGTTGC	GCCTTTCCAAGTGCATCACCTG
TGFBR1	GACAACGTCAGGTTCTGGCTCA	CCGCCACTTTCCTCTCCAAACT
TGFBR2	GTCTGTGGATGACCTGGCTAAC	GACATCGGTCTGCTTGAAGGAC
TRIM37	GCTCTGACATGCTTCTCGAAGG	CCAGATCTCCATCTGAAAGCTCG
TRPM3	GGAAGTGTTTGCGGACCAGATAG	GCACGATCCAAGCTCCTGTCTT
TSHR	GAGTTTCCTTCACCTCACACGG	CTGCTCTCATTACACATCAAGGAC
TWIST1	GCCAGGTACATCGACTTCCTCT	TCCATCCTCCAGACCGAGAAGG
WDR35	TTCGGCAGGTTTGAAGAGGCTG	GGAGACTGTCATCTGCATCACC
ZBP1	GCAAACTCCGAAGCCATCCAGA	CCAAGTTGAGGAATCACCTGGTG
ZEB2	AATGCACAGAGTGTGGCAAGGC	CTGCTGATGTGCGAACTGTAGG
ZIC1	GATGTGCGACAAGTCCTACACG	TGGAGGATTCGTAGCCAGAGCT
ZNF462	GCAATCACCTCCGAAAGCACGT	AACAAACGAGCAATACTGGCAGC

Table S2. Genes and SNPs reported in human craniosynostosis.

Official Gene Symbol	Full Gene Name	Zygosity	Variant	Syndromic vs Nonsyndromic	Type of craniosynostosis	PMID	Published Year
ABCC9	ATP binding cassette subfamily C member 9	Het	c.3605C>T, p.Thr1202Met	Cantu syndrome	sagittal and bicoronal synostosis (brachycephaly)	24352916	2014
ACTB	actin beta	Het	c.890_891delCA, p.Thr297Serfs*37 (LOF)	syndromic	sagittal synostosis	31898838	2020
ADCK1	aarF domain containing kinase 1	Het	c.467C>T, p.Thr156Met	nonsyndromic	sagittal synostosis	30651579	2019
ADAMTSL4	ADAMTS like 4	homo	c.767_786del, p.Gln256Profs*38	CS with ectopia lentis	sagittal synostosis	35378950	2022
		compound Het	c.767_786del, p.Gln256Profs*38 and c.2177+3_2177+6delGAGT	CS with ectopia lentis	bilateral coronal synostosis	35378950	2022
AHDC1	AT-hook DNA binding motif containing 1	Het	c.1206delA, p.Arg403Alafs*49	Xia-Gibbs syndrome	dolichocephaly	35596688	2022
		Het	c.1758delA, p.Lys586Asnfs*37	Xia-Gibbs syndrome	brachycephaly	35596688	2022
		Het	c.2473C>T, p.Gln825Ter	Xia-Gibbs syndrome	bicoronal synostosis	30152016	2018
		Het	c.4370 A>G, p.Asp1457Gly	Xia-Gibbs syndrome	brachycephaly	30858058	2019
ALPL	alkaline phosphatase, biomineralization associated	Het	c.212G>C, p.Arg71Pro	nonsyndromic	sagittal synostosis	30651579	2019
		Het	c.1034C>T, p.A345V	Hypophosphatasia (HPP)	multisutural bony craniosynostosis	40409424	2025
ALX4	ALX homeobox 4	homo	c.793C>T, p.Arg265Ter	Frontnasal dysostosis syndrome	coronal synostosis	19692347	2009
		Het	c.19G>T, p.Val7Phe	nonsyndromic	sagittal synostosis	22829454	2012
		Het	c.126G>C, p.Lys42Asn	nonsyndromic	sagittal synostosis	30651579	2019
		Het	c.198G>N, p.Pro306Pro	nonsyndromic	sagittal synostosis	22829454	2012
		Het	c.304G>T, p.Pro102Ser	nonsyndromic	sagittal synostosis	22829454	2012
		Het	c.398dupC, p.Pro133fs	syndromic	bilateral coronal synostosis	33369125	2021
		Het	c.467-45C>T, not protein coding	nonsyndromic	sagittal synostosis	22829454	2012
		Het	c.594C>A, p.Leu198Leu	nonsyndromic	sagittal synostosis	22829454	2012
		Het	c.631A>G, p.Lys211Glu	nonsyndromic	sagittal synostosis	22829454	2012
		Het	c.729G>A, p.Ala243Ala	nonsyndromic	sagittal synostosis	22829454	2012
		Het	c.777+70A>G, not protein coding	nonsyndromic	sagittal synostosis	22829454	2012
		Het	c.778-11G>A, not protein coding	nonsyndromic	sagittal synostosis	22829454	2012
		Het	c.879C>T, p.Leu293Leu	nonsyndromic	sagittal synostosis	22829454	2012
		Het	c.917C>T, p.Pro306Thr	nonsyndromic	sagittal synostosis	22829454	2012
		Het	c.1074C>T, p.His358His	nonsyndromic	sagittal synostosis	22829454	2012
		Het	c.1464C>T, not protein coding	nonsyndromic	sagittal synostosis	22829454	2012
ANTXR1	ANTXR cell adhesion molecule 1	homo	c.152+1G>A, not protein coding	GAPO syndrome		30575274	2019
ASPM	assembly factor for spindle microtubules	compound Het	c.1402_1406del, p.Asn468Serfs*2 and c.9697C>T, p.Arg3233Ter	microcephaly	metopic synostpsis (trigonocephaly)	31934343	2020

ASXL1	ASXL transcriptional regulator 1	Het	c.1049G>A, p.Trp350Ter	Bohring-Opitz syndrome (BOS, or BOPS)		33529703	2021
		Het	c.1210C>T, p.Arg404Ter	Bohring-Opitz syndrome	prominent metopic ridge (trigonocephaly)	21706002	2011
		Het	c.2033dupG, p.Arg678fs*6	Bohring-Opitz syndrome (BOS)	trigonocephaly	31819025	2019
		Het	c.2100dupT, p.Pro701Serfs*16	Bohring–Opitz syndrome (BOS)	trigonocephaly	26768331	2016
		Het	c.2197C>T, p.Gln733Ter	Bohring-Opitz syndrome	prominent metopic ridge (trigonocephaly)	21706002	2011
		Het	c.2332C>T, p.Gln778Ter	Bohring-Opitz syndrome	prominent metopic ridge (trigonocephaly)	21706002	2011
		Het	c.2468T>G, p.Leu823Ter	Bohring-Opitz syndrome	prominent metopic ridge (trigonocephaly)	21706002	2011
		Het	c.2535dup, p.Ser846Glnfs*5	Bohring-Opitz syndrome	prominent metopic ridge (trigonocephaly)	21706002	2011
		Het	c.2689delC, p.His897Ilefs*11	Bohring-Opitz syndrome (BOS, or BOPS)		33529703	2021
		Het	c.2893C>T, p.Arg965Ter	Bohring–Opitz syndrome (BOS)	metipic synostosis (trigonocephaly)	22419483	2012
		Het	c.3083C>A, p.Ser1028Ter	Bohring-Opitz syndrome	prominent metopic ridge (trigonocephaly)	21706002	2011
ASXL3	ASXL transcriptional regulator 3	Het	c.1897_1898delCA, p.Gln633Valfs*13	syndromic	metipic synostosis (trigonocephaly)	24044690	2013
AXIN1	axin 1	Het	c.717delG, p.T240fs*2	nonsyndromic	metopic synostosis	38905707	2024
		Het	c.1560_1561del, p.A522Efs*68	nonsyndromic	lambdoid and posterior sagittal synostosis		
		Het	c.A965G, p.E322G c.G1235A, p.R412Q c.A2187-2G, IVS10-2A>G	nonsyndromic	sagittal synostosis		
AXIN2	axin 2	Het	c.1045_1046delCT, p.Leu349fs*24	syndromic	sagittal synostosis (scaphocephaly)	30088857	2018
		Het	c.1181G>A, p.Arg394His	syndromic	sagittal synostosis	34134783	2021
B3GAT3	Beta-1,3-glucuronyltransfase 3	homo	c.598C>A, p.Pro200Thr	craniosynostosis and dental anomalies (MIM614188).	brachycephaly	29926465	2018
		homo	c.667G>A, p.Gly223Ser	syndromic	brachycephaly	28771243	2018
BBS9	Bardet-Biedl syndrome 9	Het	c.1760G>A, p.Arg587Gln	nonsyndromic	sagittal synostosis	35627201	2022
		Het	c.2209C>G, p.Leu737Val	nonsyndromic	right coronal synostosis	30651579	2019
			between rs10262453 and rs17724206	nonsyndromic	sagittal synostosis	23160099	2012
			noncoding region	nonsyndromic		35627201	2022
			rs148673350	nonsyndromic	sagittal synostosis	37973980	2023
BMP2	Bone morphogenetic protein 2		several variants in noncording region	nonsyndromic		35627201	2022
			intronic between rs1884302 and rs6140226	nonsyndromic	sagittal synostosis	23160099	2012
			rs6054748 rs1884302	nonsyndromic	sagittal synostosis	38609424	2024
			rs6054748	nonsyndromic	sagittal synostosis	37973980	2023

BMP7	Bone morphogenetic protein 7		several variants in noncording region	nonsyndromic		35627201	2022
BMPER	BMP binding endothelial regulator	Het	c.1663C>T, p.Arg555Trp	nonsyndromic	sagittal synostosis	30651579	2019
		Het	c.1663C>T, p.Arg555Trp	nonsyndromic	metopic synostosis	35627201	2022
BCOR	BCL6 corepressor	Hemi	c.1148delT	Oculofaciocardiodental syndrome (OFCD)	bicoronal and bilambdoid and squamous temporal craniosynostosis	32748437	2020
		Hemi	c.4540C>T, p.Arg1514Ter	oculo-facio-cardio-dental syndrome (OFCD)	right coronal and right lambdoid and squamous temporal craniosynostosis (brachycephaly)	28317252	2017
CASR	calcium sensing receptor	homo	c.2303G>T, p.Gly768Val	Neonatal severe primary hyperparathyroidism (NSHPT)	coronal and sphenofrontal and frontoethmoidal synostosis (brachycephaly)	24854525	2014
CASS4	Cas scaffold protein family member 4	Het	c.110T>C, p.Ile37Thr	nonsyndromic	metopic	35627201	2022
CBL	Cbl proto-oncogene	somatic	c.1111T>C, p.Tyr371His			34716204	2021
CDC45	cell division cycle 45	compound Het	c.326_329dup, p.Asn111Ilefs*11 and c.1512C >T, p.His504His	Meier-Gorlin syndrome 7 (MGS7)	bicoronal synostosis (turribrachycephaly)	34000999	2021
		compound Het	c.1541_1544del, p.Lys514 Thrfs*10 and a synonymous variant in trans in exon 7	Meier-Gorlin syndrome (MGORS)	coronal synostosis	33639314	2021
		Het	c.36G>C	22q11.2 deletion syndrome	metopic and bicoronal synostosis	31474763	2020
		Het	c.241G>A, p.Val81Ile	22q11.2 deletion syndrome	unilateral coronal synostosis	31474763	2020
		Het	c.847C>T, p.Arg283Cys	22q11.2 deletion syndrome	bicoronal synostosis	31474763	2020
		Het	c.204G>A, p.Gln68	Meier-Gorlin syndrome 7 (MGS7)		38467731	2024
		compound Het	c.1416C>T, p.His472				
		compound Het	c.*1+5G>A				
CEP135	centrosomal protein 135	compound Het	c.2863C>T, p.Arg955Ter and c.1372_1375del, p.Lys459Serfs*77	nonsyndromic Primary microcephaly (MCPH)		33933664	2021
CETP	chlesteryl ester transfer protein	homo	c.1207C>T, p.Arg403Ter	syndromic	metopic synostosis	26740239	2016
CIMIP1	ciliary microtuble inner protein 1	Het	c.60T>G, p.Asp20Glu	nonsyndromic	metopic synostosis	35627201	2022
CHD7	chromodomain helicase DNA binding protein 7	Het	c.3106C>T, p.Arg1036Ter	CHARGE syndrome	bicoronal and metopic and left lamboid and squamouse synostosis (turricephaly)	30498854	2019
		Het	c.6157C>T, p.Arg2053Ter	CHARGE syndrome	bicoronal synostosis	33844462	2021
CHST3	carbohydrate sulfotransferase 3	homo	c.407_426dup, p.Thr143Cysfs*80	Larsen syndrome	sagittal synostosis	24300290	2014
CNOT2	CCR4-NOT transcription complex subunit 2	Het	c.1396A>C, p.Asn466His	syndromic	either brachycephaly, oxycephaly, and plagiocephaly	36224108	2023

		Het	c.1482 T>G, p.Tyr494Ter	syndromic	either brachycephaly, oxycephaly, and plagiocephaly	36224108	2023
		Het	c.1622A>T, p.Ter541Leuext*65 (extension of encoded protein)	syndromic	either brachycephaly, oxycephaly, and plagiocephaly	36224108	2023
		Het	ch12 microdeletion	syndromic	either brachycephaly, oxycephaly, and plagiocephaly	36224108	2023
COLEC10	collection subfamily member 10	homo	c.128_129delCA, p.Thr43Asnfs*799	3M syndrome	craniosynostosis (dolichocephaly) w/o CL/P	34636477	2021
		compound Het	c.25C>T, p.Arg9Ter and c.226delA, p.Gly77Glufs*66	3MC syndrome	w/unilateral CLP	28301481	2017
		compound Het	c.25C>T, p. Arg9Ter and c.528C>G, p.Cys176Trp	3MC syndrome	w/bilateral CLP	28301481	2017
COLEC11	collection subfamily member 11	homo	c. 89_98delATGACGCCTG, p.Asp30Alafs*68	3MC syndrome		28301481	2017
		homo	c.309delT, p.Gly104Valfs*29	3MC syndrome		28301481	2017
		homo	c.496G>A, p.Ala166Thr	3MC syndrome		28301481	2017
CPT1A	carnitine palmitoyltransferase 1A	compound Het	c.1163+1G>A and c.1393G>A, p.Gly465Arg	syndromic (CPT1A deficiency)	sagittal synostosis (scaphocephaly)	27066452	2016
CRTAP	cartilage associated protein	homo	c.118G>T, p.Glu40Ter	Cole-Carpenter syndrome (CCS)	bicoronal and bilambdoid synostosis	25604815	2015
CTCF	CCCTC-binding factor	Het	c.612delAAAG, p.Lys206Profs*13	syndromic	microbrachycephaly	28619046	2017
CTCFL	CCCTC-binding factor like	Het	c.1159C>G, p.Ala387Pro	nonsyndromic	metopic synostosis	35627201	2022
		Het	c.520C>T, p.Ala174Thr	nonsyndromic	metopic synostosis	35627201	2022
CTNNB1	catenin beta 1	Het	c.1759C>T, p.Arg587Ter	syndromic		34558805	2022
CTSK	cathepsin K	homo	c.244-29A>G (intronic-not affecte to protein coding)	Pycnodysostosis (PKND)		33963797	2021
		homo	c.618+2T>G, splice donor	Pycnodysostosis (PKND)	unicoronal synostosis	35186389	2020
		Het	c.436G>C, p.Gly146Arg	syndromic with Pycnodysostosis (PKND)		34680947	2021
		Het	c.509G>T, p.Cys170Phe	syndromic with Pycnodysostosis (PKND)		34680947	2021
		Het	c.761_763delCCT, p.Ser255 (inframe deletion)	syndromic with Pycnodysostosis (PKND)		34680947	2021
CYP17A1	cytochrome P450 family 17 subfamily A member 1	Het	c.684C>T, p.Thr228Ile	Antley-Bixler syndrome		15491389	2004
CYP21A2	cytochrome P450 family 21 subfamily A member 2	compound Het	c.IVS2–13A/C>G and p.Pro30Leu	P450 oxidoreductase (POR) deficiency		17389698	2007
CYP26B1	cytochrome P450 family 26 subfamily B member 1	homo	c.1088G>T, p.Arg363Leu	syndromic	cornal synostosis and broadened sagittal suture	22019272	2011
		homo	c.1303G>A, p.Gly435Ser	syndromic	sagittal & coronal synostosis (brachycephaly)	27410456	2016

		compound Het	c.376G>T, p.Gly126Cys and c.701G>A, p.Arg234Gln	syndromic	coronal and metopic and lamboid synostosis	34160123	2021
		compound Het	c.1088G>A, p.Arg363His and c.1190G>A, p.Arg397Gln	syndromic	coronal and metopic synostosis	34160123	2021
		compound Het	c.1088G>A, p.Arg363His and c.1190G>A, p.Arg397Gln	syndromic	coronal and metopic and lamboid synostosis	34160123	2021
				Crouzon syndrome		Embase only: Gazi Med. J. - Volume 33, Issue 1, P55	2022
DDX3X	DEAD-box helicase 3 X-linked	Het	c.625C>G, p.His209Asp	DDX3X syndrome (MRXSSB)	brachycephaly	33789733	2021
DIAPH1	diaphanous related formin 1	homo	c.3145C>T; p.Arg1049Ter	syndromic	metopic synostosis (trigonocephaly)	26463574	2016
DIS3L2	DIS3 like 3'-5' exoribonuclease 2	Het	c.695C>G, p.Ser232Ter	syndromic with Wilms tumor	plagiocephaly	35700413	2023
DLG1	discs large Maguk scaffold protein 1	Het	c.521G>C, p.Pro174Arg	nonsyndromic	sagittal synostosis	35627201	2022
DLX6-AS1	DLX6 antisense RNA 1		rs17656761	nonsyndromic	coronal synostosis	39345948	2024
DMPK	DM1 protein kinase	homo	2 copies of expansion mutation of 1260 and 60 CTG repeats	Myotonic dystrophy type 1	sagittal and left coronal and metopic synostosis (anterior plagiocephaly)	18474935	2008
DPH1	diphthamide biosynthesis 1	homo	c.17T>A, p.Met6Lys	syndromic	sagittal synostosis (scaphocephaly)	26220823	2015
EFNA4	ephrin A4	Het	c.178C>T, p.His60Tyr	Ulnar-mammary syndrome (UMS)	sagittal synostosis	36140816	2022
EFNB1	ephrin B1	Het	see supplemental table S2a	Craniofrontonasal syndrome (CFNS)	uni or by coronal synostosis		
EFTUD2	elongation factor Tu GTP binding domain containing 2	Het	c.491A>G p.Asp164Gly	acrofacial dysostosis Guion-Almeida type	metopic synostosis (trigonocephaly)	33262786	2020
		Het	c.779T>A p.Ile260Asn	acrofacial dysostosis Guion-Almeida type	trigonocephaly	33262786	2020
ERCC2	ERCC excision repair 2, TFIH core complex helicase subunit	compound Het	c.1201G>C, p.Ala401Pro and c.2164C>T, p.Arg722Trp	syndromic with Trichothiodystrophy (TTD)	bicoronal and metopic synostosis	31803976	2020
ERF	ETS2 repressor factor	Het	c.3G>A, p.0		metopic synostosis	23354439	2013
		Het	c.21A>T, p.Gly8_Phe9ins147		sagittal synostosis	23354439	2013
		Het	c.194G>A, p.Arg65Gln		metopic synostosis	23354439	2013
		Het	c.256C>T, p.Arg86Cys		metopic synostosis	23354439	2013
		Het	c.256C>T, p.Arg86Cys		sagittal synostosis	23354439	2013
		Het	c.286A>T, p.Lys96Ter	ERF-related craniosynostosis syndrome type 4 (CRS4)	pansynostosis	35852485	2022
		Het	c.547C>T, p.Arg183Ter		pansynostosis (cloverleaf skull)	23354439	2013
		Het	c.547C>T, p.Arg183Ter		metopic and sagittal and left coronal synostosis	23354439	2013
		Het	c.547C>T, p.Arg183Ter		metopic synostosis	23354439	2013

		Het	c.891_892delAG, p.Gly299Argfs*9		unilateral lamboid synostosis	23354439	2013
		Het	c.891_892delAG, p.Gly299Argfs*9		metopic synostosis	23354439	2013
		Het	c.1270C>T, p.Gln424Ter		pansynostosis (cloverleaf skull)	23354439	2013
		Het	c.1201_1202delAA, p.Lys401Glufs*10		pansynostosis (cloverleaf skull)	23354439	2013
		Het	c.1512delT, p.Phe504Leufs*27		pansynostosis (cloverleaf skull)	23354439	2013
		Het	c.1201_1202del, p.Lys401Glufs*10	Noonan syndrome		38824261	2024
		Het	c.185del, p.Glu62Glyfs*15	Noonan syndrome		38741564	2024
ESCO2	establishment of sister chromatid cohesion N-acetyltransferase 2	homo	c.417delA, p.Lys139Asnfs*6	Roberts syndrom		31192177	2019
		homo	c.1131+1G>A, p.Arg338fs*17	Roberts syndrom		31192177	2019
FAM20C	FAM20C golgi associated secretory pathway kinase	Het	c.1094G>A, p.Gly365Asp	Raine syndrome	pansynostosis	27862258	2017
		Het	c.1094G>A, p.Gly365Asp	Raine syndrome	metipic synostosis (trigonocephaly)	27862258	2017
FAM209A	family with sequence similarity 209 member A	Het	c.165G>T, p.Trp55Cys	nonsyndromic	metopic synostosis	35627201	2022
		Het	c.409A>G, p.Met137Val	nonsyndromic	metopic synostosis	35627201	2022
FBN1	fibrillin 1	Het	c.3217G > A, p.Glu1073Lys	Marfan syndrome	right occipital synostosis	16596670	2006
		Het	c.3302G > A, p.Tyr1101Cys	Marfan syndrome	anterior sagittal synostosis and maybe coronal synostosis (scaphocephaly)	16596670	2006
		Het	c.3442C>G, p.Pro1148Ala	Shprintzen-Goldberg syndrome (SGS)		8563763	1996
		Het	c.3662G >A, p.Cys1221Tyr	Shprintzen-Goldberg syndrome (SGS)	dolichocephaly	16333834	2006
		Het	c.3668G>A, p.Tyr1223Cys	Shprintzen-Goldberg syndrome (SGS)		8563763	1996
FGF9	fibroblast growth factor 9	Het	c.184A> G, p.Arg62Gly	syndromic	sagittal synostosis (dolicocephaly)	28730625	2017
FGF23	fibroblast growth factor 23	Het	c.527G > A, p.Arg176Gln	autosomal dominant hypophosphatemic rickets (ADHR)	sagittal craniosynostosis	38988138	2024
FGFR1	fibroblast growth factor receptor 1	Het	see supplemental table S2b	Pfeiffer syndrome, Osteoglophnic dysplasia, Nonsyndromic			
FGFR2	fibroblast growth factor receptor 2	Het/Homo	see supplemental table S2c-l	Apert syndrome, Beare-Stevenson syndrome, Crouzon syndrome, Pfeiffer syndrome, 46XY gonadal dysgenesis, Nonsyndromic			
FGFR3	fibroblast growth factor receptor 3	Het	see supplemental table S2m-q	Thanatophoric dysplasia type I & type II, Muenke syndrome, achondroplasia, Nonsyndromic			

FGFR4	fibroblast growth factor receptor 4	Het	c.C>T, p.Pro136Leu, rs376618	Apert syndrome		30258940	2017
		Het	c.1162G>A, p.Gly388Arg	Apert syndrome		30258940	2017
		Het	c.G>A, p.Ala485Thr, rs201831200	Apert syndrome		30258940	2017
FGFRL1	fibroblast growth factor receptor like 1	compound Het	p.Ala287Pro and c.AGgtacca into Agatacca	Antley-Bixler syndrome		19056490	2009
FLNA	filamin A	Hemi	c.3557C>T, p.Ser1186Leu	Frontometaphy seal dysplasia 1 (FMD1)	pansynostosis	34277511	2021
FOXP1	forkhead box P1	Het	c.1428+1G>A, p.Ala450GLyfs*13	Opitz C trigonocephaly syndrome	metopic synostosis (trigonocephaly)	29330474	2018
FREM1	FRAS1 related extracellular matrix 1	Het	c.1394G>C, p.Gly465Ala	nonsyndromic	either sagittal or coronal synostosis	30651579	2019
		Het	c.1493G>A; p.Arg498Gln	nonsyndromic	metopic synostosis (trigonocephaly)	21931569	2011
		Het	c.3819T>A, p.Asp1273Glu	nonsyndromic	either sagittal or coronal synostosis	30651579	2019
		Het	c.4499A>T; p.Glu1500Val	nonsyndromic	metopic synostosis (trigonocephaly)	21931569	2011
FTO	FTO alpha-ketoglutarate dependent dioxygenase	homo	c.812A>C, p.His271Pro	syndromic	metopic synostosis	26740239	2016
		homo	c.965G>A, p.Arg322Gln	syndromic		26697951	2016
GINS2	GINS complex subunit 2	homo	c.341G>T, p.Arg114Leu	Meier-Gorlin syndrome (MGORS)	bilateral coronal synostosis	34353863	2022
GLI3	GLI family zinc finger 3	Het	c.327del, p.Phe109Leufs*50	Greig cephalopolysyn dactyly syndrome (GCPS)	scaphocephaly	24736735	2015
		Het	c.1018delA, p.Ser340Valfs*7	Pallister-Hall, Grieg cephalopolysyn dactyly syndrome-like	metopic synostosis (trigonocephaly)	20583172	2010
		Het	c.1728C>A, p.Tyr576Ter	Greig cephalopolysyn dactyly syndrome (GCPS)	metipic and saggital synostosis (trigonocephaly)	21326280	2011
		Het	c.1793dupA, pAsn598Lysfs*7	Greig cephalopolysyn dactyly syndrome (GCPS)	metipic synostosis (trigonocephaly)	21326280	2011
		Het	c.2786T > C, p.Leu929Pro	Acrocallosal syndrome (ACS)	saggital and bilamboid synostosis (dolichocephaly)	23633388	2013
		Het	c.4542_4545delCCA C, p.His1515Profs*3	Pallister-Hall, Grieg cephalopolysyn dactyly syndrome-like	metopic synostosis (trigonocephaly)	20583172	2010
		Het	8.3-Mb deletion 7p12.3-p14.1	Greig cephalopolysyn dactyly syndrome (GCPS)	metipic synostosis (trigonocephaly)	21326280	2011
		Het	6.8 Mb-deletion 7p13-p14.1	Greig cephalopolysyn dactyly syndrome (GCPS)	metipic and saggital synostosis (trigonocephaly and scaphocephaly)	21326280	2011
		Het	6.0-Mb deletion 7p13-p14.1	Greig cephalopolysyn dactyly syndrome (GCPS)	metipic synostosis (trigonocephaly)	21326280	2011

		Het	del(7)(p14.1)(GLI3-)[56]/7p14.1(GLI3x2)[44]	Greig cephalopolysyndactyly syndrome (GCPS	trigonocephaly	24736735	2015
GLIS3	GLIS family zinc finger 3	compound Het	c.2298_2657+del and c.2298_2657+del	syndromic	sagittal synostosis	26259131	2015
GNAS	GNAS complex locus	Het	c.1A>C, p.Met1Val	Albright's hereditary osteodystrophy (AHO) associated with pseudohypoparathyroidism type 1A	partial sagittal and lambdoid synostosis	28396140	2017
		Het	c.286A > G, p.K96 c.758A > G, p.Y253C c.691C > T, p.R231C	nonsyndromic and syndromic		41307550	2026
GNPTAB	N-acetylglucosamine-1-phosohate transferase subunits alpha and beta	homo	c.136C>T, p.Arg46Ter	Mucolipidosis II (MLII)	multiple synostosis	24060719	2013
GPC3	glypican 3	Het	c.1077C>T, p.Ser359Ser (MANE: c.1239C>T, p.Ser413Ser)	Simpson-Golabi-Behmel syndrome (SGBS)	left coronal synostosis	24115482	2013
HADHA	hydroxyacyl-CoA dehydrogenase trifunctional multienzyme complex subunit alpha	Het	c.1324G > C, p.D442H		multiple cranial sutures, including metopic suture, coronal suture, sagittal suture and lambdoid suture	37977316	2024
HDAC9	histone deacetylase 9	Het	336kb deletion at TWIST1 regulatory location (7p21.1)	syndromic	bicoronal synostosis (brachycephaly)	35710300	2022
		Het	401kb deletion at TWIST1 regulatory location	syndromic	sagittal synostosis (brachycephaly)	35710300	2022
		Het	1759kb deletion	Saethre-Chotzen syndromie like phenotype	sagittal and bicoronal synostosis	31754721	2020
		Het	t(7;12)(p21.2;p12.3)	Saethre-Chotzen syndromie like phenotype	coronal synostosis (brachycephaly)	21708297	2011
			structual variant			Embase only: Eur. J. Hum. Genet. - Volume 28, Issue 0, pp. 74-75	2020
HNRNPK	heterogeneous nuclear ribonucleoprotein K	Het	p.52Lys_56AsninsLeuLeuGln, g.86591976A>C	Au–Kline syndrome	scaphocephaly	32588992	2020
		Het	c.257G > A, p.Arg85His (splice site)		possible & matipic ridge	26173930	2015
		Het	c.931_932insTT, p.Pro311Leufs*40		sagittal synostosis & matipic ridge	26954065	2016
		Het	c.953+1dup, p.Gly319Argfs*6	Kabuki syndrome	Sagittal and lambdoid synostosis (plagiocephaly)	26173930	2015
HSD3B7	hydroxy-delta-5-steroid dehydrogenase, 3 beta- and steroid delta-isomerase 7	homo	c.531 + 1G>C	nonsyndromic		39803807	2025
HUWE1	HECT, UBA and WWE domain containing E3	Hemi	c.145-2A>G, p.Cys49-Glu50del, g.53674519T>C	Say-Meyer syndrome	trigonocephaly	30797980	2020

	ubiquitin protein ligase 1	Het	c.328 C > T, p.Arg110Trp	syndromic		29180823	2017
		Het	c.329 G > A, p.Arg110Gln	syndromic	unicoronal and metopic synostosis or multiple synostosis	29180823	2017
IDS	iduronate 2-sulfatase		c.823G>A, p.D275N	Hunter syndrome	scaphocephaly	39097272	2024
IFT81	Intraflagellar transport 81	compound Het	c.1934_1937delinsGAAG, p.Leu645Ter and c.1557+3_1557+6del	Short-rib polydactyly syndromes (ciliopathy)	sagittal synostosis (dolichocephaly)	32783357	2020
IFT122	Intraflagellar transport 122	homo	c.1868G>T, p.Gly623Val	Cranioectodermal dysplasia (CED)	sagittal synostosis (dolichocephaly)	24689072	2014
		compound Het	c.1862T>G, p.F621C and c.3228insG, p.Tyr1077Valfs*11	Cranioectodermal dysplasia type 1 (CED)	complex multisynostosis	26792575	2016
		compound Het	c.2288T>C, p.763P and c.2005-13T>A	Cranioectodermal dysplasia type 1 (CED)	complex multisynostosis	26792575	2016
		compound Het	c.3184G>C, p.Ala1062Pro and c.3228dupG;c.3231_3233delCAT, p.Tyr1077Valfs*10	Beemer–Langer syndrome	turribrachycephaly	28370949	2017
		compound Het	c.3385C>T and c.628C>T, p.Arg210Trp (MANE: c.475C>T, p.Arg159Trp)	Cranioectodermal dysplasia (CED)	metopic synostosis	29037998	2018
IFT140	Intraflagellar transport 140	compound Het	c.326T>C, p.Leu109Pro and c.3454-488_4182+2588dup, p.Tyr1152_Thr1394dup	Sensenbrenner syndrome	dolichocephaly	32007091	2020
		compound Het	c.3141+1G>T (splice donor) and c.-11_6del	Opitz C trigonocephaly syndrome (OTCS)	metopic synostosis (trigonocephaly)	27874174	2017
		Het	c.903-13(IV S8)T>G	Short-rib thoracic dysplasia		41307550	2026
IGF1R	Insulin lime growth factor 1 receptor	Het	c.568C>T, p.Pro190Ser	nonsyndromic	coronal synostosis	21204214	2011
		Het	p.Arg406His	nonsyndromic	sagittal synostosis	21204214	2011
		Het	c.1336A>G, p.Met446Val	nonsyndromic	coronal synostosis	21204214	2011
		Het	c.1784G>A, p.Arg595His	nonsyndromic	coronal synostosis	21204214	2011
		Het	c.2570A>G, p.Asn857Ser	nonsyndromic	sagittal synostosis	21204214	2011
IL11RA	Interleukin 11 receptor subunit alpha	homo	c.707T>C, p.Leu236Pro	Crouzon-like craniosynostosis syndrome with associated dental anomalies (CRSDA)	bicoronal and sagittal synostosis	32277509	2020
		homo	c.328G>T, p.Gly110Cys	craniosynostosis and dental anomalies (MIM614188).	coronal synostosis (brachycephaly)		
		homo	c.916_924dup, p.Thr306_Ser308dup	craniosynostosis and dental anomalies (MIM614188).	brachycephaly		
		compound Het	C.281G>T, p.Cys94Phe and c.781C>T, p.Arg261Cys	craniosynostosis and dental anomalies (MIM614188).	coronal and lamboid synostosis	29926465	2018

		compound Het	c.328G > T, p.Gly110Cys and c.866A > G, p.His289Arg	craniosynostosis and dental anomalies (MIM614188).	sagittal synostosis (brachycephaly)		
		compound Het	c.916_924dup, p.Thr306_Ser308dup and c.781C > T, p.Arg261Cys	Crouzon-like craniosynostosis syndrome with associated dental anomalies (CRSDA)	sagittal synostosis	30811827	2019
		compound Het	c.598C>A, p.Pro200Thr and c.710G>C, p.Arg237Pro	Crouzon syndrome -like with midfacial hypoplasia, variable exophthalmos, relative prognathiism	Pansynostosis	24498618	2013
		Homo	c.696C>A, p.Tyr232Ter c.874C>T, p.Arg292Ter c.479+6T>G, p.Gly338Gln	Crouzon syndrome -like with midfacial hypoplasia, variable exophthalmos, relative prognathiism	Pansynostosis		
		homo	c.692G>T, p.Gly231Val c.811-2A>G c.707 T>C, p.Leu236Pro c.700C>T, p.Arg234Ter c.395G>A, p.Trp132Ter	Crouzon-like craniosynostosis syndrome with associated dental anomalies (CRSDA)		40353334	2025
		homo	exon 9, p.E364_V368del			37994264	2024
ITPA	inosine triphosphatase	Het	c.136 C > T, p.Gln46	familial epilepsy		41003830	2025
JAG1	Jagged canonical Notch ligand 1	Het	c.2070G>T, p.Glu553Ter	Alagille syndrome	unilateral coronal synostosis	12244552	2002
		Het	c.2504delGAAAG	Alagille syndrome	unilateral coronal synostosis	12244552	2002
		Het	c.2740G>A, p.Gly914Arg	nonsyndromic	coronal synostosis	30651579	2019
KAT6A	lysine acetyltransferase 6A	compound Het	c.3782delC, p.Pro1261Leufs*33 and c.3780delT, p.Pro1261euLfs*33	syndromic	late onset pansynostosis	33770237	2022
		Het	c.3399_3400dup, p.Lys1134Argfs*14	Rett syndrome	sagittal synostosis	33386251	2022
		Het	c.3661G>T, p.Glu1221Ter	Rett syndrome	sagittal synostosis	33386251	2022
KAT6B	lysine acetyltransferase 6B	Het	c.4205_4206delCT, p.Ser1402Cysfs*5	Lin-Gettig syndrome	sagittal synostosis	28696035	2017
		Het	c.4572dupT, p.Thr1525Tyrfs*16	Lin-Gettig syndrome	sagittal synostosis	28696035	2017
KMT2D	Lysine methyltransferase 2D	Het	c.8200C>T, pArg2734Ter	Kabuki syndrome		29914387	2018
KRAS	KRAS proto-oncogene, GTPase	Het	c.173C>T, p.Thr58Ile	Noonan syndrome	sagittal and pertial bicoronal synostosis	19396835	2009
		Het	c.178G>A, p.Gly60Ser	Noonan syndrome	left lamboid synostosis	19396835	2009
		Het	c.214A>C or c.214A>T, p.Met72Leu	Noonan syndrome	metopic synostosis (trigonocephaly)	22488932	2012
		Het	c.178G>C, p.Gly60Arg	Noonan syndrome Cardiofaciocutaneous syndrome		37774117	2024

LHX3	LIM homeobox 3	homo	c.437G>T, p. Cys146Phe	Congenital combined pituitary hormone deficiency (CPHD)	sagittal synostosis (dolichocephaly)	28302169	2017
		homo	c.466C>T, p. Arg156Ter	Congenital combined pituitary hormone deficiency (CPHD)	sagittal synostosis (dolichocephaly)	28302169	2017
LRP5	LDL receptor related protein 5	Het	c.640G>A , p.Ala214Thr	syndromic with high bone mass phenotype	pansynostosis or metopic synostosis (trigonocephaly) or coronal and sagittal synostosis	15940380	2005
LTBP1	latent transforming growth factor beta binding protein 1	homo	c.1342C>T, p.Gln448Ter	cutis laxa syndrome	dolichocephaly	34861177	2021
		homo	c.1342C>T, p.Gln448Ter	syndromic	pansynostosis	33991472 (erratum 34861177)	2021
		homo	c.3991dup, p.Thr1331Asnfs*22	syndromic	right coronal and sagittal synostosis	33991472 (erratum 34861177)	2021
		homo	c.4431T>A, p.Cys1477Ter	cutis laxa syndrome	brachycephaly	34861177	2021
		homo	c.4431T>A, p.Cys1477Ter	syndromic	coronal synostosis or coronal and sagittal and lambdoid synostosis	33991472 (erratum 34861177)	2021
MAGEL2	MAGE family member L2	Het	c.1912C>T, p.Gln638Ter	Opitz C trigonocephaly syndrome (OTCS)	matopic synostosis (trigonocephaly)	28281571	2017
MAP4K4	mitogen-activated protein kinase kinase kinase kinase 4	Het	c.569G>T, p.Gly190Val	RASopathies (distinct class of neurodevelopmental syndromes)	multiple sutures	38679877	2024
MAP3K20	mitogen-activated protein kinase kinase kinase 20	Het	c.837_839delCAA, p.Asn279del	syndromic		38451290	2024
MASP1	MBL associated serine protease 1	homo	c.9G>A, p.Trp3Ter	Malpuech–Michels–Mingarelli–Carnevale (3MC) syndrome		28301481	2017
		homo	c.2111T>G, p.Val704Gly	Malpuech–Michels–Mingarelli–Carnevale (3MC) syndrome	CS	30601195	2019
MBTPS1	membrane bound transcription factor peptidase, site 1	homo	c.2948G>A, p.Trp983Ter	spondyloepiphyseal dysplasia	bicoronal and sagittal synostosis	32420688	2020
MCPH1	microcephalin 1	Het	c. 302C>G, p.Ser101Ter	Craniosynostosis-microcephaly with chromosomal breakage and other abnormalities (CMCB)		20101680	2010
MCM5	minichromosome maintenance complex component 5	Het	c.1591-3C > T		multiple cranial sutures, including metopic suture, coronal suture, sagittal suture and lambdoid suture	37977316	2024

MEIS2	Meis homeobox 2	Het	microdeletion at 17q11.2 c.27_28del, p.His10Leufs*84	NF1 microdeletion syndrome	bicoronal craniosynostosis	33722742	2021
MED13L	mediator complex subunit 13L	Het	chr12:116,440,797-116,549,327, mosaic microdeletion	MED13L haploinsufficiency syndrome	metopic and sagittal synostosis	28371282	2017
MEGF8	multiple EGF like domains 8	homo	c.595G>C, p.Gly199Arg	Carpenter syndrome (CRPT)	metopic and sagittal synostosis	23063620	2012
		homo	c.4496G>A, p.Arg1499His	Carpenter syndrome (CRPT)	bicoronal synostosis (trigonocephaly)	23063620	2012
		compound Het	c.1342C>T, p.Arg448* and c.7099A>G, p.Ser2367Gly	Carpenter syndrome (CRPT)	metopic synostosis	23063620	2012
		compound Het	c.3349+3_3349+4dupAA and c.7069-2A>G (splice donor)	Carpenter syndrome (CRPT)	metopic synostosis	23063620	2012
		Het	c.4657(exon 27)G>A, p.V1553I(p.Val1553Ile)	Carpenter syndrome (CRPT)		41307550	2026
		Het	p.Leu293Pro, p.Arg2376Cys and p.Val2713Met	Carpenter syndrome (CRPT)		38760421	2024
MSX2	msh homeobox 2	Het	c.443C>T, p.Pro148Leu	Boston-type craniosynostosis syndrome	sagittal and metopic synostosis (scaphocephaly and trigonocephaly)	23918290	2013
		Het	c.443C>T, p.Pro148Leu	nonsyndromic or syndromic	sagittal synostosis	23918290	2013
		Het	c.443C>T, p.Pro148Leu	syndromic	left coronal synostosis	23918290	2013
		Het (copy number)	46,XX.arr 4q22.1(88,282,368-88,336,595)x1, 5q35.2(173,984,926-174,145,340)(174,718,444-174,907,513)x5, 8q24.3(142,215,092-142,412,727)x1, 9q34.2q34.3(136,982,786-137,166,802)x1	syndromic	coronal synostosis	22948472	2012
MYADML2	myeloid associated differentiation marker like 2	homo	7.3Kb deletion (17q25.3)	syndromic	sagittal synostosis	32778762	2021
NELL1	neural EGFL like 1	Het	c.368A>G, p.Asp123Gly	nonsyndromic	left coronal synostosis	30651579	2019
NFIA	nuclear factor 1A	Het	1p31.3, 109Kb microdeletion	syndromic	sagittal or lamboid synostosis	25714559	2015
NOTCH1	notch receptor 1	Het	c.2734C>T, p.Arg912Trp	nonsyndromic	sagittal synostosis	30651579	2019
NOTCH2	notch receptor 2	Het	c.1076C>T, p.Ser359Phe	Alagille syndrome (ALGS) type2		36201396	2022
		Het	c.7223T>A, p.Leu2408His	nonsyndromic	sagittal synostosis	30651579	2019
OSGEP	O-sialoglycoprotein endopeptidase	Het	c.689 G>T, p.C230F	Galloway-Mowat syndrome type 3 (GAMOS3)	microcephaly	39661309	2025
OSTM1	osteoclastogenesis associated transmembrane protein 1	homo	c. 365A>T, p.Val122Asp	syndromic	sagittal and bicoronal synostosis	23772242	2013
P4HB	prolyl 4-hydroxylase subunit beta	Het	c.1178A>G, p.Tyr393Cys	Cole-Carpenter syndrome (CCS)	coronal synostosis	30063094	2018
		Het	c.1178A>G, p.Tyr393Cys	Cole-Carpenter syndrome (CCS)	coronal and metopic synostosis	30063094	2018

		Het	c.1178A>G, p.Tyr393Cys	Cole-Carpenter syndrome	coronal and frontal synostosis	25683117	2015
		Het	deletion of ex5-8	Cole-Carpenter syndrome (CCS)	pansynostosis	29384951	2017
PCK1	phosphoenolpyruvate carboxykinase 1	Het	c.410G>A, p.Arg137his	nonsyndromic	metopic	35627201	2022
		Het	c.413C>T, p.THr138Ile	nonsyndromic	metopic	35627201	2022
PCCA	propionyl-CoA carboxylase subunit alpha		rs7981517	nonsyndromic	coronal synostosis	39345948	2024
PDILT	protein disulfide isomerase like, testis expressed	Het	c.1243C>T, p.Pro415Ser	nonsyndromic	sagittal synostosis	30651579	2019
PLEKHA6	pleckstrin homology domain containing A6		rs114264214	nonsyndromic	coronal synostosis	39345948	2024
POR	cytochrome p450 oxidoreductase	homo	c.458A>G, p.Gln153Arg	Antley-Bixler syndrome		15793702	2005
		homo	c.859G>C, p.Ala287Pro	Antley-Bixler syndrome	brachycephaly	15793702	2005
		homo	c.859G>C, p.Ala287Pro	Antley-Bixley syndrome		14758361	2004
		homo	c.1370G>A, p.Arg457His	Antley-Bixler syndrome	brachycephaly	15793702	2005
		compound Het	c.424A>G, p.Thr142Ala and frameshift	Antley-Bixler syndrome	brachycephaly	15793702	2005
		compound Het	c.667C>T, p.Arg223Ter and c.1223T>A, p.Met408Lys	Antley-Bixley syndrome	hammered silver" appearance	23295302	2012
		compound Het	c.859G>C, p.Ala287Pro and 7q11.2 (75608488_75615534) 7Kb deletion	Antley-Bixler syndrome	too early for detect CS	26969897	2016
		compound Het	c.859G>C, p.Ala287Pro and c.1845C>T, p.Arg616Ter	Antley-Bixler syndrome	cloverleaf skull, brachycephaly	15793702	2005
		compound Het	c.859G>C, p.Ala287Pro and c.732-2A>T (splice acceptor)	Antley-Bixler syndrome	coronal synostosis (trapezoidocephaly)	26670660	2015
		compound Het	c.859G>C, p.Ala287Pro and frameshift	Antley-Bixler syndrome		15793702	2005
		compound Het	c.859G>C, p.Ala287Pro and frameshift	Antley-Bixler syndrome	brachycephaly	15793702	2005
		compound Het	c.859G>C, p.Ala287Pro and frameshift	Antley-Bixler syndrome	brachycephaly	15793702	2005
		compound Het	c.859G>C, p.Ala287Pro and intronic mutation	Antley-Bixler syndrome	brachycephaly	15793702	2005
		compound Het	c.1329insC and c.1370G>A, p.Arg454His	Antley-Bixler syndrome	brachycephaly	15264278	2004
		compound Het	c.1329_1330insC, p.I444Hfs*6 and c.1370G>;A, p.R457H	Antley-Bixler syndrome		32615689	2020
		compound Het	c.1370G>A, p.Arg457His and microdeletion in exon5	Antley-Bixler syndrome	cloverleaf skull	35070845	2021
		compound Het	c.1370G>A, p.Arg457His and ?	Antley-Bixler syndrome		16470797	2006

		compound Het	c.1370G>A, p.Arg457His and c.1375T>C, p.Tyr459His	Antley-Bixler syndrome		15793702	2005
		compound Het	c.1370G>A, p.Arg457His and c.1694T>C, p.Leu565Pro	Antley-Bixler syndrome		15793702	2005
		compound Het	c.1370G>A, p.Arg457His and c.731+1G>A	Antley-Bixley syndrome		14758361	2004
		compound Het	c.1370G>A, p.Arg457His and intronic mutation	Antley-Bixler syndrome	brachycephaly	15793702	2005
		compound Het	c.1615G>A, p.Gly539Arg and frameshift	Antley-Bixler syndrome	sagittal and lambdoid synostosis	15793702	2005
		compound Het	c.1937-1939delTCT, p.Phe646del and p.Ala287Pro	Antley-Bixler syndrome	brachycephaly	15793702	2005
		compound Het	c.1694T>C, p.Leu565Pro and c.1370G>A, p.Arg457His	Antley-Bixler syndrome	brachycephaly	15793702	2005
		Het	p.Ala115Val	Antley-Bixler syndrome		15793702	2005
		Het	c.787A>G, p.Met263Val	Antley-Bixler syndrome		15793702	2005
		Het	c.859G>C, p.Ala287Pro	Antley-Bixler syndrome	cloverleaf skull	15793702	2005
		Het	c.1370G>A, p.Arg457His	Antley-Bixler syndrome	cloverleaf skull	15793702	2005
		Het	c.1370G>A, p.Arg457His	Antley-Bixler syndrome		16470797	2006
		Het	c.1475T>A, p.Val492Glu	Antley-Bixley syndrome		14758361	2004
PPP1CB	protein phosphatase 1 catalytic subunit beta	Het	c.146C>G, p.Pro49Arg	RASopathies	sagittal and pertial bicoronal synostosis	28211982	2017
PPP2R1A	protein phosphatase 2 scaffold subunit Alpha	Het	c.773G > A, p.Arg258His	syndromic	metopic synostosis	34716204	2021
PTCH1	patched 1	Het	c.1502Adup, p.Val502Glyfs*13	Gorlin syndrome	pansynostosis	31578813	2020
PTH2R	parathyroid hormone 2 receptor	Het	inversion of 2q14.3 and 2q34	nonsyndromic	sagittal and metopic synostosis	26044810	2015
PTPN11	protein tyrosine phosphatase non-receptor type 11	Het		Noonan syndrome		37774117	2024
			p.Thr2Ile	Noonan syndrome	bilateral coronal craniosynostosis	39484914	2025
			p.Gln256Arg	Noonan syndrome	unicoronal and lambdoidal craniosynostosis		
PTPRD	protein tyrosine phosphatase receptor type D	homo	deletion of exon 9, a part of intron 9, and exons 10 to 15	syndromic	trigonocephaly	26082802	2015
RAB5IF	RAB5 interacting factor	homo	c.75G>A, p.Trp25Ter	Cerebrofaciothoracic Dysplasia (CFTD)	brachycephaly	36150687	2022
RAB23	RAB23, member RAS oncogene family	homo	c.35T>A, p.Met12Lys	Carpenter syndrome (CRPT)	multiple synostosis	21412941	2011
		homo	c.86dupA, p.Tyr29Ter	Carpenter syndrome (CRPT)	cloverleaf skull to metopic ridge	20358613	2010
		homo	c.140_141insA, p.Glu48fs*7	Carpenter syndrome (CRPT)	metopic and sagittal and bicoronal synostosis or multiple synostosis	17503333	2007

		homo	c.232delT, p.Tyr78fs*30	Carpenter syndrome (CRPT)	multiple synostosis	17503333	2007
		homo	c.398+1G>A (splice donor)	Carpenter syndrome 1 (CRPT1)		34748996	2022
		homo	c.234_236delCTA, p.Tyr79del	Carpenter syndrome (CRPT)	multiple synostosis	21412941	2011
		homo	c.362 363insG, p.Asn121fs*4	Carpenter syndrome (CRPT)	multiple synostosis	21412941	2011
		homo	c.408_409insT, p.Glu137Ter	Carpenter syndrome (CRPT)	metopic and sagittal and bicoronal and unilamboid synostosis or sagittal and bicoronal synostosis	17503333	2007
		homo	c.416T>C, p.Leu139Pro	Carpenter syndrome 1 (CRPT1)		34748996	2022
		homo	C.434T>A, p.Leu145Ter	Carpenter syndrome (CRPT)	metopic and sagittal and bicoronal synostosis	17503333	2007
		homo	c.481G>C, p.Val161Leufs*16	Carpenter syndrome (CRPT)	metopic and partial sagittal and left lambdoid synostosis	25168863	2014
		homo	c.482-1G>A, p.Val161fs*3	Carpenter syndrome (CRPT)	pansynostosis (cloverleaf skull)	23599695	2013
		compound Het	c.82C>T, p.Arg28Ter and c.434T>A, p.Leu145Ter	Carpenter syndrome (CRPT)	multiple synostosis	21412941	2011
		compound Het	c.253T>C, p.Cys85Arg and c.434T>A, p.Leu145Ter	Carpenter syndrome (CRPT)	metopic and sagittal synostosis	17503333	2007
		compound Het	c.434T>A, p.Leu145Ter and C.156-3T>G, p.Val53fs*13	Carpenter syndrome (CRPT)	multiple synostosis	21412941	2011
		Het	c.546A>C	nonsyndromic	sagittal synostosis	26910679	2016
RBM38	RNA binding motif protein 38	Het	c.479G>A, p.Ala160Thr	nonsyndromic	metopic synostosis	35627201	2022
		Het	c.661G>A, p.Ala221Thr	nonsyndromic	metopic synostosis	35627201	2022
RECQL4	RecQ like helicase 4	homo	g.5428A>C, c.IVS17-2A>C	Baller-Gerold syndrome (BGS)	lamboid and coronal synostosis (turribrachycephaly)	15964893	2006
		compound Het	c.2492_2493delAT, p.His831Argfs*52 and c.2506_2518del13bp, p.Thr836Trpfs*3	Baller-Gerold syndrome (BGS) with Rothmund-Thomson syndrome-like phenotype	right cornal synostosis (brachycephaly)	19291770	2009
		compound Het	g.2881G>C, p.Ser523Thr (cosegregate g.2886delT)and g.5435C>T, p.Arg1021Trp	Baller-Gerold syndrome (BGS)	multiple synostosis or bilamboidand bicoronal synostosis (brachycephaly)	15964893	2006
		compound Het	c.3061C>T, p.Arg1021Trp and c.1573delT, p.Cys525Alafs*33	Rothmund–Thomson syndrome (RTS)		24635570	2015
		Het	c.212A>G, p.Glu71Gly	nonsyndromic	sagittal synostosis	30651579	2019
		Het	c.1565G>A, p.Arg522His	nonsyndromic	sagittal synostosis	30651579	2019

		Het	c.2340G>T, p.Pro780Pro	nonsyndromic	sagittal synostosis	30651579	2019
		Het	c.2237C>T, p.Ala746Val	nonsyndromic	sagittal synostosis	30651579	2019
RNU12	RNA, U12 small nuclear	compound Het	n.75A>G, g.43011324A>G, rs552666394 and n.*3C>T (no c. number)	CDAGS Syndrome	brachycephaly	34085356	2021
		compound Het	n.77T>C, g.43011326T>A, rs768684008 and n.*3C>T (no c. number)	CDAGS Syndrome	bilateral coronal synostosis (brachycephaly)	34085356	2021
		compound Het	n.86G>A, g.43011335G>A, rs548281798 and n.*3C>T, g.43011402C>T (no c. number)	CDAGS Syndrome	bilateral coronal synostosis	34085356	2021
RTF2	Replication termination factor 2	Het	c.109T>G, p.Cys37Trp	nonsyndromic	metopic	35627201	2022
RUNX2	RUNX family transcription factor 2	Het	c.225GGCGGCTGCGGCGGCGGC[1], p.Ala84-Ala89del, rs11498192 (GOF)	nonsyndromic		32360898	2020
		Het	c.751C>T, p.Arg251Cys (GOF)	nonsyndromic		32360898	2020
		Het	c. 1000G>A, p.Asp334NAsn (GOF)	nonsyndromic		32360898	2020
		Het	c. 1361A>G, p.Tyr454Cys	nonsyndromic		32360898	2020
		Het	c.1489G>A, p.Gly497Ser	nonsyndromic		32360898	2020
		Het	c. 1531G>A, p.Gly511Ser(LOF)	nonsyndromic		32360898	2020
		Het	c.IVS6+46 G>A	nonsyndromic		32360898	2020
			quadruplication	syndromic		23348268	2013
SCN4A	sodium voltage-gated channel alpha subunit 4	compound Het	c.3425G>A, p.Arg1142Gln and c.1123T>C, p.Cys375Arg	congenital myopathy	sagittal and metopic synostosis (scaphocephaly)	28262468	2017
SEM1	SEM1 26S proteasome subunit		rs4727341	nonsyndromic	coronal synostosis	39345948	2024
SDHAF3	succinate dehydrogenase complex assembly factor 3		rs12154925	nonsyndromic	coronal synostosis	39345948	2024
SH3PXD2B	SH3 and PX domains 2B	Het	c.970C>T, p.Arg324Trp	nonsyndromic	sagittal synostosis	30651579	2019
		homo	complete deletion of ex13	Frank-ter Haar syndrome	sagittal synostosis	23140272	2012
SHC4	SHC adaptor protein 4	Het	c.1810A>G, p.Ile604Val	nonsyndromic	coronal synostosis	30651579	2019
SHH	sonic hedgehog signaling molecule	Het	c.50T>C, p.Leu17Pro	syndromic with holoprosencephaly		10762164	2000
SHOC2	SHOC2 leucine repeat scaffold protein	Het	c.4A>G p.Ser2Gly	syndromic (Noonan-like facial dysmorphism)	right coronal and bilateral lambdoid and sagittal synostosis	25123707	2014
SKI	SKI proto-oncogene	compound Het	c.346G>A, p.Gly116Arg and c.687G>C, p.Lys229Asn	Shprintzen-Goldberg syndrome (SGS)	sagittal & coronal synostosis (brachycephaly)	34177429	2021
		Het	c.82T>A, p.Ser28Thr	Shprintzen-Goldberg syndrome (SGS)	dolichocephaly	24736733	2015
		Het	c.92C>T, p.Ser31Leu	Shprintzen-Goldberg		23103230	2012

				syndrome (SGS)			
		Het	c.94C>G, p.Leu32Val	Shprintzen-Goldberg syndrome (SGS)		23103230	2012
		Het	c.94C>G, p.Leu32Val	Shprintzen-Goldberg syndrome (SGS)		23103230	2012
		Het	c.95T>C, p.Leu32Pro	Shprintzen-Goldberg syndrome (SGS)		23103230	2012
		Het	c.100G>A, p.Gly34Ser	Shprintzen-Goldberg syndrome (SGS)	dolichocephaly	24736733	2015
		Het	c.100G>A, p.Gly34Ser	Shprintzen-Goldberg syndrome (SGS)		23103230	2012
		Het	c.100G>T, p.Gly34Cys	Shprintzen-Goldberg syndrome (SGS)		23103230	2012
		Het	c.103C>T, p.Pro35Ser	Shprintzen-Goldberg syndrome (SGS)	dolichocephaly	24736733	2015
		Het	c.101G>A, p.Gly34Asp	Shprintzen-Goldberg syndrome (SGS)	dolichocephaly	24736733	2015
		Het	c.101G>A, p.Gly34Asp	Shprintzen-Goldberg syndrome (SGS)	dolichocephaly	24736733	2015
		Het	c.101G>T, p.Gly34Val	Shprintzen-Goldberg syndrome (SGS)		23103230	2012
		Het	c.103C>T, p.Pro35Ser	Shprintzen-Goldberg syndrome (SGS)		23103230	2012
		Het	c.104C>A, p.Pro35Gln	Shprintzen-Goldberg syndrome (SGS)		23103230	2012
		Het	c.289_300del, p.Ser97_Arg100del	Shprintzen-Goldberg syndrome (SGS)	dolichocephaly	24736733	2015
SLC25A24	solute carrier family 25A member 24	Het	c.650G>A, p.Arg217His	Fontaine progeroid syndrome (FPS)	brachycephaly	36093452	2022
		Het	c.650G>A, p.Arg217His	Fontaine progeroid syndrome (FPS)	brachycephaly		
SMC1A	structural maintenance of chromosomes 1A	Het	c.3581A>G	Cornelia de Lange syndrome	metopic synostosis	29037998	2018
SMAD3	SMAD family member 3	Het	c.221G > T, p.Arg74Leu	Loeys-Dietz syndrome (LDS)		40464278	2025
SMAD6	SMAD family member 6	Het	c.465_471del c.1296dupC c.43C>T, p.Arg15 c.793C>T, p,His265Tyr c.817G>A,		trigonocephaly	40488688	2025

			p.Glu273Lys c.857A>G, p.Asp286Gly				
		Het	c.874+4A>G		scaphocephaly		
		homo	c.584T>G; c.584T>G, p.Val195Gly; p.Val195Gly c.817G>A; c.817G>A, p.[Glu273Lys,Glu273Serfs*72];[Glu273Lys,Glu273Serfs*72]		metopic synostosis	38290823	2024
		Het	c.41G > A: p.W14*		multiple cranial sutures, including metopic suture, coronal suture, sagittal suture and lambdoid suture, as well as the fontanelle	37977316	2024
		Het	c.787C>T, p.Pro263Ser	complex congenital heart disease (CHD)		40133303	2025
SPAG17	sperm associated antigen 17	homo	c.1069G > C; p.Asp357His	Cranioectodermal dysplasia (CED)	dolichocephaly	29174089	2018
SPO11	SPO11 initiator of meiotic double strand breaks	Het	c.547A>G, p.Arg183Gly	nonsyndromic	metopic	35627201	2022
SPRY1	sprouty RTK signaling antagonist 1	homo	c.80T>A, p.Leu27Ter	syndromic	saggital synostosis and bilateral subcutaneous cystic lesions over the lambdoid sutures(scaphocephaly & turricephaly)	36543535	2022
TBX3	T-box transcription factor 3	Het	c.804 + 1G>A, g.IVS3 + 1G>A	Ulnar-mammary syndrome (UMS)	sagittal synostosis	36140816	2022
TCF12	transcription factor 12	Het	See supplementary table S2r-s	Syndromic (Saethre-Chotzen syndrome-like), Nonsyndromic			
TCOF1	treacle ribosome biogenesis factor 1	Het	c.2731C>T, p.Arg911Stop	Treacher Collines syndrome	bicoronal and right squamosal and lambdoid synostosis	15214011	2004
TCTN3	tectonic family member 3	homo	c.257-1G>A	syndromic	scaphocephaly	36039988	2022
TEDC1	tubulin epsilon and delta complex 1	compound Het	c.104-5C>G;787delG, p.Ala263LeufsTer29	syndromic		39979680	2025
TGFBR1	transforming growth factor beta receptor 1	compound Het	c.239G>A; p.Arg80Gln and c.313C>G; p.His105Asp	Loeys-Dietz syndrome (LDS)		36584339	2023
		Het	c.722C > T, p.Ser241Leu	Marfan syndrome	sagittal synostosis	16596670	2006
		Het	c.722C > T, p.Ser241Leu	Marfan syndrome	dolichocephaly	16596670	2006
		Het	c.934G>A, p.Gly312Ser	syndromic with thoracic aortic aneurysms and dissections (TAAD)	dolichocephaly	19542084	2009
		Het	c.953T>G, p.Met318Arg	syndromic		15731757	2005
		Het	c.1199A>G, p.Asp400Gly	syndromic		15731757	2005

			in preperation	Loeys-Dietz syndrome (LDS) type II	dolichocephaly	18978651	2009
TGFBR2	transforming growth factor beta receptor 2	Het	c.95-2A>G, splice site	syndromic		15731757	2005
		Het	c.1069G>T, p.Gly357Trp	syndromic		15731757	2005
		Het	c.1547C>A, p.Thr516Lys	Shprintzen-Goldberg syndrome (SGS)	sagittal synostosis (scaphocephaly)	17979970	2008
		Het	c.1582C>T, p.Arg528Cys	Loeys-Dietz syndrome (LDS)	sagittal synostosis (scaphocephaly)	19875893	2009
		Het	c.1583G>A , p.Arg528His	Loeys-Dietz syndrome (LDS)	squamosal suture synostosis	22488992	2012
		Het	c.1732T>A, p.Ser578Thr	nonsyndromic	sagittal synostosis	30651579	2019
		Het	c.IVS5-2A >G	Shprintzen-Goldberg syndrome (SGS)	dolichocephaly	16333834	2006
			in preperation	Loeys-Dietz syndrome (LDS) type II	dolichocephaly	18978651	2009
TGFBR3	transforming growth factor beta receptor 3	homo/Het	c.2418G>A, p.Trp806Ter	syndromic	Mercedes-Benz pattern craniosynostosis (bilateral lambdoid and posterior sagittal synostosis, 'BLSS')	41312766	2025
TRAF7	TNF receptor associated factor 7	Het	c.1204C>G, p.Leu402Val	syndromic	metopic and sagittal synostosis	32459067	2020
		Het	c.1921C >T, p.Arg641Cys	syndromic	sagittal synostosis (scaphocephaly)	35733823	2022
		Het	1964G>A, p.Arg655Gln	syndromic	metopic and sagittal synostosis	32459067	2020
TRIM37	tripartite motif containing 37	homo	c.1295A>G, p.Gln432Arg	syndromic		26697951	2016
TRPM3	transient receptor potential cation channel subfamily M member 3	Het	c.2509G>A, p.Val837Met	syndromic	metopic synostpsis (trigonocephaly)	34438093	2021
TRPV4	transient receptor potential cation channel subfamily V member 4	Het	c.496C>A, p.Leu166Met	nonsyndromic	sagittal synostosis	36905673	2023
TSHR	hyroid stimulating hormone receptor	Het	c.842G>A, p.Ser281Asn	congenital hyperthyroidism		9589634	1998
		Het	c.1514A>G, p.Ser505Asn	congenital hyperthyroidism		9360555	1997
		Het	c.1535T>A, p.Leu512Gln	congenital hyperthyroidism		16960398	2006
		Het	c.1899C>A, p.Asp633Glu	congenital hyperthyroidism		30599487	2018
		Het	c. 2016 T>G, p.Cys672Trp	syndromic with thyrotoxicosis	bicoronal synostosis	29225840	2017
TWIST1	twist family bHLH transcription factor 1	Het	see supplemental table S2t-w	Saether-Chotzen syndrome, Baller-Gerold syndrome, Nonsyndromic			
WDR19	WD repeat domain 19	compound Het	c.3533G>A, p.Arg1178Gln and c.953delA, p.Asn319Ilefs*16	Sensenbrenner syndrome	dolichocephaly	28621010	2017

		compound Het	c.3533G>A, p.Arg1178Gln and c.2654+1G>T	Sensenbrenner syndrome	dolichocephaly	28621010	2017
WDR35	WD repeat domain 35	homo	c.1415G>A, p.Arg472Gln	Cranioectodermal dysplasia (CED)	dolichocephaly	29174089	2018
		homo	c.1559T>C, p.Leu520Pro, g.20146297A>G	Sensenbrenner syndrome	sagittal synostosis	22987818	2012
		homo	c.2912A>G, p.Tyr971Cys	Sensenbrenner syndrome (Cranioectodermal dysplasia: CED)	dolichocephaly	22486404	2013
		compound Het	c.3G>A, p.Met1-Ala30delinsMetfs*4 and c.2522A>T, p.Asp841Val	Cranioectodermal dysplasia (CED)	sagittal synostosis and pertial left lamboid synostosis	32804427	2020
		compound Het	c.3G>A, p.Met1-Ala30delinsMetfs*4 and c.2522A>T, p.Asp841Val	Cranioectodermal dysplasia (CED)	sagittal synostosis (dolicocephaly)	32804427	2020
		compound Het	c.337C>T, p.Arg113Ter and c.2522A>T, p.Asp841Val	Sensenbrenner syndrome (cranioectodermal dysplasia, CED)	dolichocephaly	29134781	2018
		compound Het	c.504T>A, p.Ser168Arg and c.1922T>G, p.Leu641Ter	Sensenbrenner syndrome (Cranioectodermal dysplasia: CED)	dolichocephaly	22486404	2013
		compound Het	c.907G>A, p.Gly303Arg and c.1922T>G, p.Leu641*, rs199952377	Cranioectodermal dysplasia (CED)	saggital & coronal & lamboid synostosys (dlichocephaly)	35875935	2022
		compound Het	c.1922T>G, p.Leu641Ter and c.2522A>T, p.Asp841Val	Cranioectodermal dysplasia (CED)		33421337	2021
		compound Het	c.1922T>G, p.Leu641Ter and c.2522A>T, p.Asp841Val	Cranioectodermal dysplasia (CED)	sagittal and right coronal synostosis (dolichocephaly and plagiocephaly)	28332779	2017
		compound Het	c.1877A>G, p.Glu626Gly and c.25-2A>G,p.Ile9Thrfs*7	Sensenbrenner syndrome		20817137	2010
		compound Het	c.2891delT, p.Pro964Leufs*15 and c.2623G>A, p.Ala875Thr	Sensenbrenner syndrome		20817137	2010
ZBP1	Z-DNA binding protein 1	Het	c.172C>A, p.Val58Phe	nonsyndromic	metopic	35627201	2022
ZEB2	zinc finger E-box binding homeobox 2	Het	c.714delA	Mowat-Wilson syndrome (MWS)	right cornal synostosis (brachycephaly)	18076118	2008
		Het	p.Glu181Argfs*211	Mowat–Wilson syndrome (MWS)	right coronal synostosis	25123255	2014
		Het	c.855_856del, p.Glu286Valfs*7	Mowat-Wilson syndrome		26809768	2016
		Het	c.3544del, p.Gly1182Lysfs*59	Mowat–Wilson syndrome (MWS)	right coronal synostosis	25123255	2014
ZIC1	Zic family member 1	Het	c.1183C>G, p.Pro395Ala	syndromic	caput membranaceum and partial bicoronal craniosynostosis	32975022	2020
		Het	c.1214_1215delinsCCACCATCGTG, p.Pro406fs	syndromic		30391508	2018

		Het	e novo inversion of chromosome 3q	Gomez-Lopez-Hernandez syndrome	bicoronal craniosynostosis	37950019	2024
ZNF462	zinc finger protein 462	Het	c.763C>T, p.Arg255Ter	syndromic	metopic synostosis	31361404	2019
		Het	c.6794dup, p.Tyr2265Ter	syndromic	metopic synostosis	31361404	2019
		Het	c.3787C>T, p.Arg1263Ter	syndromic	metopic synostosis	31361404	2019
		Het	c.2979_2980delinsA, p.Val994Trpfs*147	syndromic	metopic synostosis	31361404	2019
		Het	c.4263del, p.Glu1422Serfs*6	syndromic	metopic synostosis	31361404	2019
		Het	c.6214_6215del, p.His2072Tyrfs*8	syndromic	metopic synostosis	31361404	2019
DLX5 / TAC1			rs78353978	nonsyndromic	coronal synostosis	39345948	2024
SM1M23 / FGF18			rs33863	nonsyndromic	coronal synostosis	39345948	2024

Table S3. Structural variants reported in human craniosynostosis.

Cytogenetic band or Genomic coordinates	Genes in suggestive loci	Zygosity	Structual variant	Syndromic vs Nonsyndromic	Type of craniosynostosis	PMID	Published Year
1p31.1-1p31.3 deletion		Het	1p31.1-1p31.3 microdeletion, 8.04Mb	Congenital Hypopituitarism	metipic synostosis (trigonocephaly)	29264484	2017
1p31 deletion	*LEPR, JAK1,* and *NFIA*	Het	8.04Mb deletion of 1p31.1-1p31.3	syndromic with coongenital hypopituitarism	metopic ridge (trigonocephaly)	29264484	2017
1p36.3 trisomy and 1p36.3 deletion		choromosomal compound HT	t(Y;1)(q12;p36.3) and 1p36.3-pter [46,X,der(Y)t(Y:1)(q12;p36.3)]	syndromic	metopic and sagittal synostosis	16835918	2006
translocation of 1p31 to 18q11			t(1;18)(p31;q11)	syndromic	coronal and lamboid synostosis	8320714	1993
partial monosomy 1p36.1 and partial trisomy 17q25.1		Het	46,XY,der(1)t(1;17)(p36.3;q25.1)	Pfeiffer-type cardiocranial syndrome	sagittal synostosis	16531733	2006
1q41deletion		Het	7.84 Mb deletion at 1q41 (215,199,578–223,035,427	Loeys-Dietz syndrome (LDS)	brachycephaly	35426477	2022
1q41deletion			1.44 Mb deletion at 1q41 (217,589,671–219,026,274)	Loeys-Dietz syndrome (LDS)	dolichocephaly	35426477	2022
1q41deletion			3.99 Mb deletion at 1q41 (216,243,817–220,231,236)	Loeys-Dietz syndrome (LDS)	turricephaly	35426477	2022
1q41deletion			785 kb deletion at 1q41 (218,238,773–219,024,035)	Loeys-Dietz syndrome (LDS)	dolichocephaly	35426477	2022
1q trisomy and 6q monosomy		compound Het	der(6)t(1;6)(q42.13;q27)pat; 1q42.13-->qter, 20.7Mb duplication and 6q27-->qter, 3.6Mb deletion	syndromic	brachycephaly	23431743	2012
1q43 duplication	*RGS7*	Het	1q43 duplication (1.65Mb)	nonsyndromic	unilateral coronal synostosis	20683987	2010
chromosomal rearrangement (1; 4; 7) with a microdeletion of 7p21.3-7p15.3	*TWIS1*	Het		compound Saethre–Chotzen syndrome	metopic and pertial lamboid synostosis	25118508	2014
1q22-q23.1 duplication	*LMNA* and *BGLAP*	Het	1.26 Mb duplication at 1q22-q23.1	syndromic	metipic and unilateral lamboid synostosis (trigonocephaly)	29845577	2018
translocation of 2p14 to 7p12 and deletion 7p21.3	*TWIST1*	Het	t(2;7)(p24;p21),ins(7)(p21.3q21.3q22) dn	Saethre-Chotzen syndrome (SCS)	turricephaly	18255367	2008
2p14-16.3 deletion		homo	rs1073981 to rs719293 (microsatellite: D2S1352, D2S378, D2S337 and D2S1342)	syndromic		15561999	2004
2p21 deletion	*SIX2*	Het	2p21, 108.3 Kb deletion	Frontonasal dysplasia	sagittal synostosis	26581443	2016
2p21 deletion	*PRKCE*	Het	2p21 duplication (0.4Mb)	nonsyndromic	sagittal synostosis	20683987	2010
translocation of 2q221 to 7p21.2	*TWIS1*	Het	46,XY,t(2,7)(q21.1;p21.2)	Saethre-Chotzen syndrome (SCS)		9259286	1997
translocation of 2p23 to 7p22			t(2;7)(p23;p22)	Saethre-Chotzen syndrome (SCS)		8266989	1993
translocation of 2p21 and 7p15	*HDAC9* and *MSCC1*	Het	t(2;7)(p21;p15)	syndromic		Embase only:Erciyes. Med.	2019

						J. Volume 41	
translocation of2q21.1 to 7p21.2		Het	t(2;7)(q21.1;p21.2)	Saethre-Chotzen syndrome-like	coronal synostosis	7783164	1995
2q3 trisomy and distal 7p22 monosomy			2q3 trisomy and distal 7p22 monosomy	syndromic	trigonocephaly	3874588	1985
2q22 deletion	*ZEB2*	Het	0.6Mb 2q22 deletion	Mowat–Wilson syndrome	bicoroanl synostosis (brachycephaly)	18445050	2008
2q31.1 deletion		Het	2q24.3q32.1, 19.7 Mb microdeletion	syndromic	metopic and partial coronal synostosis	20425826	2010
2q37.3 deletion 5q34 duplication	*MSX2*	choromosomal compound HT	2q37.3Del and 5q34qter	syndromic	metopic and coronal synostosis	19533795	2009
2q14 duplication	*DPP10*	Het	2q14 duplication (0.73Mb)	nonsyndromic	sagittal synostosis	20683987	2010
duplication of 2q35	*IHH*	Het	31 kb duplication at 2q35 (219,658,383–~219,689,640): upstream regulatory region of IHH	syndromic	sagittal or sagittal and right coronal synostosys	25692887	2015
balanced translocation t(2;15)(q21;q21.3)	*TCF12*		balanced translocation t(2;15)(q21;q21.3)	syndromic	right coronal synostosis (Plagiocephaly)	24648389	2014
translocation of 2q32.1 to 7p21.3		Het	chromozomal translocation of 2q32.1 and 7p21.3	nonsyndromic	metopic and bicoronal synostosis (turricephaly)	27774767	2017
deletion-translocation of 2p to 11q or 12q			t(2p-; Cq +)	syndromic	sclerosis on bicoronal and sagittal sutures and metopic synostosis	5493837	1970
2q deletion, der(2) and translocation of 2q37 to 15q26			46,XY,-2,+der(2),t(2;15)(q37;q26)pat	syndromic	metopic and sagittal suture ridge and lambdoid synostosis (scaphocephaly)	1621758	1992
Chr 2 duplication	*IHH*	Het	48Kb	craniosynostosis Philadelphia type	sagittal synostosis	211647467	2011
Chr 2 duplication	*IHH*	Het	52Kb	craniosynostosis Philadelphia type	sagittal synostosis	211647467	2011
Chr 2 duplication	*IHH*	Het	52Kb	craniosynostosis Philadelphia type	pansynostosis (cloverleaf skull)	211647467	2011
2q deletion		Het	deletion of 2q24.3 and 2q31 and 2q32(possible)	syndromic	left coronal and sagittal synostosis	9188674	1997
rearrangment of 2p, 2q and 9p, and 9p22p23 deletion		Het	der(2)(9pter → 9p23::2q13 → 2q23::2p25.1 → 2q13::9p22.2 → 9p21.3::2q36 → 2q23::2p25.1 → 2pter),der(9)(2qter → 2q36:: 9p21.3 → 9qter)	9p deletion syndrome	metopic synostosis (trigonocephaly)	16419130	2006
3p deletion		Het	3p26.3p25.2, 11.8 Mb deletion	3p deletion syndrome	trigonocephaly	36374187	2022
der(3)(3;7)(p25;q36), with partial monosomy of 3pter and partial trisomy of 7qter	*SMN1*	choromosomal compound HT	der(3)(3;7)(p25;q36), with partial monosomy of 3pter and partial trisomy of 7qter	syndromic	left coronal and bilateral lamboid synostosis (turricephaly)	19215052	2009
3p25 duplication	*FBLN2* and *TMRM43*	Het	3p25 duplication (3.34Mb)	nonsyndromic	sagittal synostosis	20683987	2010
3p;9p translocation	*TYRP*	Het	3p11 and 9p23 translocation	9p syndrome	metopic synostosis	7825591	1995

3q trisomy and translocation of 3q21 to 10p15			46, XX,-10,t(3;10)(q21;p15)	syndromic	turricephaly	7468658	1980
deletion in 4p, 4q, and 11q		complex	4p16.1-p15.32 (9.76 Mb), 4q31.1 (881 Kb), and 11q22.1 (771 Kb)	syndromic	sagittal & coronal synostosis (brachycephaly)	35932041	2022
4p15 deletion		Het	4p15.32p15.31, 4Mb microdeletion	nonsyndromic	metopic synostosis	21910230	2011
4p trisomy		Het	46,XY,−2,+der(2)t(2;4)(q37.3;p15.1)	syndromic	coronal and lambdoidal and temporoparietal synostosis (cloverleaf skull)	10353793	1999
4q13 and 7p15 deletion	*TWIST1*	compound Het	4q13.2q13.3 and 7p21.1p15.3 deletion	Saethre-Chotzen like phenotype		23825006	2013
partial trisomy 4 and translocation of 2q33 to 16q22		Het	47,XY,+der(4)del(4)(q13.2?).ish t(2;16)(q33;q22)(wcp16+;wcp2+),add(4) (?;q13.2?)(wcp4+)	syndromic		Embase only:Erciyes. Med. J. - Volume 39 (2)	2017
4q27q28.3 deletion		Het	4q27q28.3, 11 Mb deletion	syndromic	partial sagittal synostosis	24980605	2014
5p15 duplication	*SEMA5A* and *FASTKD3*	Het	5p15 duplication (2.47Mb)	nonsyndromic	sagittal synostosis	20683987	2010
inversion of 5p and insertion of 5q and duplication of 5q	*MSX2*	Het	inversion of 5p13p14, a direct insertion of 5q11q13, and inverted insertion of 5q23q31 and duplicated 5q35	syndromic	metopic and sagittal and lambdoid synostosis	18000908	2007
translocation of 5p15.3 to 7p21.2		Het	t(5;7)(p15.3;p21.2)	Saethre-Chotzen syndrome-like	bicoronal synostosis (brachycephaly)	7783164	1995
5p monosomy			t(5;13) (13pter leads to 13q13 : : 5p14 leads to 5qter)	syndromic		752064	1978
5q35.2 duplication	*NSD1*	Het	5q35.2 duplication	syndromic (Sotos syndrome like)	metopic synostosis	23599694	2013
5q trisomy and 9p monosomy		choromosomal compound HT	der(9)t(5;9)(q34;p23)mat.ish der(9)t(5;9)(q34;p23)(9pter-,5qter+).arr 5q34q35(163,328,000-180,629,000)×3, 9p24p23(194,000-12,664,000)×1	Hunter-McAlpine syndrome-like phenotype	multiple synostosis	21063078	2011
distal 5q trisomy	*MSX2*	Het	der(10)t(5;10)(q33;q26.3)	syndromic	sagittal and metopic and lambdoid synostosis	17955513	2007
distal 5q trisomy		Het	der(17)t(5;17)(q35.1;p13.3)	syndromic	metopic synostosis	17955513	2007
5q34 trysomy	*MSX2*	Het	der(13)t(5;13)(q33.3;q34)mat	syndromic	sagittal and bilamboid synostosis (oxycephaly)	15214020	2004
5q duplication		Het	dulication of 5q11.2-5q14	syndromic	sagittal and bicoronal and lambdoid synostosis (scaphocephaly)	8244339	1993

multiple rearrangement including 5q pentaplication	*Msx2*	Het	46,XX.arr 4q22.1(88,282,368-88,336,595)x1, 5q35.2(173,984,926-174,145,340)(174,718,444-174,907,513)x5, 8q24.3(142,215,092-142,412,727)x1, 9q34.2q34.3(136,982,786-137,166,802)x1	syndromic	coornal synostosis	22948472	2012
pertial trisomy 6p			6p21.1 to 6cen, 16Mb trisomy	syndromic	sagittal synostosis (brachycephaly with mild plagiocephaly)	17431916	2007
6p partial monosomy and 10q partial trysomy		choromosomal compound HT	unbalanced rearrangement, der(6)t(6:10)(p23;q24)mat	syndromic	coronal and sagittal synostosis	23074675	2011
6p21 duplication	*RUNX2*	Het	6p21 duplication (1.1Mb)	with hypodontia	metopic synostosis	20683987	2010
translocation of 6q16.2 to 7p15.3			t(6;7)(q16.2;p15.3)	7p deletion syndrome	sagittal and bicoronal synostosis	7909651	1994
6q trisomy and translocation of 6q26 and 22p12			t(6;22)(q26;p12)	6q trisomy syndrome	brachycephaly	7273465	1981
6q21q22.31 deletion		Het	8.9Mb 6q21-q22.31 deletion	syndromic	metopic synostosis	27038333	2016
6q22.2q23.1 deletion		Het	6q22.2q23.1 deletion	syndromic		3359667	1988
translocation of 6q22, 12q24 and 15q21	*FBN1*			Marfan syndrome		22260333	2012
7p microdeletion		Het	7p22.3p22.2 deletion	syndromic	metopic synostosis	28440577	2017
7p21 deletion	*HDAC9* and *TWIST1*	Het	7p21 0.9 Mb microdelation	Saethre-Chotzen syndrome (SCS)	right coronal synostosis (brachycephaly)	28220539	2018
7p21.1p15.3 deletion	*TWIST1*	Het	148 Mb 46,XY.ish del(7p21.1)(RP11-5G13-),7q11.23(RP11-805G2x2).arr 7p21.1p15.3(15,957,375-20,331,837)x17p21.1p15.3 deletion	Saethre-Chotzen syndrome (SCS)	left coronal and metopic synostosis	23958897	2013
pertial trisomy of 7p and 15q		compound Het	interstitial duplications of 7p22.1p21.1 and 15q24.1	syndromic	may be CS	29610688	2018
7p21[46,XX,add(7)(p21)], 7p21.1, 3p21.31 rearrangement	*TWIST1, SNX13, PRPS1L1, HD9C9, FERD3L, CACNA2D2, C3orf18, HEMK1, CISH, MAPKAPK3,* and *DOCK3*	Het	7p21[46,XX,add(7)(p21)] (deletion of TWIST1), 7p21.1 (deeletion of SNX13, PRPS1L1, HD9C9, and FERD3L) , 3p21.31 (deletion of CACNA2D2, C3orf18, HEMK1, CISH, MAPKAPK3, and DOCK3)	Saethre-Chotzen like phenotype	bicoronal synostosis (brachycephaly)	22628249	2012
7p15.3p21.3 deletion			del(7)(p15.3p21.3)	syndromic	coronal synostosis and marrowing of sagittal and metopic sutures (brachycephaly)	4043965	1985
7p15 deletion, and 1p22 and 7p15 translocation			t(1;7)(p22;p15),del(7)(pter--*p15:)	syndromic	coronal and metopic synostosis (turricephaly)	489013	1979
7p15p21 deletion	*TWIST1*	Het	7p15-p21 deletion	Saethre-Chotzen syndrome	coronal synostosis	22569119	2012

7p deletion			7p pertial deletion	syndromic		112608	1975
7p13 deletion		Het	7p13-->7p14	7p deletion syndrome		7521123	1994
7p21 deletion		Het	7p21-->7p22	7p deletion syndrome		7521123	1994
7p microdeletion	*TWIST, DFNA5, CYCS, HOXA11, HOXA13,* and *GARS*	Het	7p21-p14.3, 13M microdeletion (including TWIST, DFNA5, CYCS, HOXA11, HOXA13, and GARS)	syndromic	left coornal synostosis (left metopic plagiocephaly)	22570644	2011
7p21 deletion and 7p/18q translocation	*TWIST1*	Het	t(7;18)(p15.3;q11.2) and 7p21.2 and 7p21.3, 7.6-10.6Mb deletion	syndromic	multiple synostosis (brachycephaly)	17786117	2007
7p21 deletion	*TWIST1*	Het	del(7)(p15.3p21.3)	Saethre-Chotzen syndrome (SCS)	bicoronal synostosis	12116251	2002
7p21 deletion	*TWIST1*	Het		Saethre-Chotzen syndrome (SCS)	bicoronal synostosis	11280946	2001
7p21.2p15.2 deletion			del(7)(pter----p21.2::p15.2----qter)	7p21 monosomy syndrome		3487273	1986
Chr7 deletiom	*HDAC9, TWIST1, SP8,* and *DNAH11*	Het	between 18498435-21940887	Crouzon syndrome		Embase only: Gazi Med. J. - Volume 33, Issue 1, pp. P55	2022
trnslocation of 7p21.2 to 18q23	*TWIST1*	Het	46,XX,t(7,18)(p21.2;q23)	Saethre-Chotzen syndrome (SCS)		9259286	1997
translocation of 7p21.2 to 10q21.2	*TWIST1*	Het	46,XY,t(7,10)(p21.2;q21.2)	Saethre-Chotzen syndrome (SCS)		9259286	1997
translocation of 7p21.2 to 18q23		Het	t(7;18)(p21.2;q23)	Saethre-Chotzen syndrome-like	coronal synostosis	7783164	1995
7p21.2 translocation		Het	between D7S488 and D7S493	Saethre-Chotzen syndrome		7987323	1994
7p22		Het	between D7S664 and D7S507	Saethre-Chotzen syndrome		7977380	1995
translocation of 7p21.2 to 10q21.2			t(7;10)(p21.2;q21.2)	Saethre-Chotzen syndrome (SCS)		8266988	1993
monosomy 7p15		Het	monosomy 7p15 --> pter	Saethre-Chotzen syndrome (SCS)	sagittal and coronal and lambdoid synostosis	18019370	2007
7q deletion	*BRAF*	Het	7q32.3-q35, 12.782Mb deletion	syndromic (Noonan-like facial dysmorphism)	coronal and sagittal and squamous synostosis	32529787	2020
7q36 deletion	*ESYT2*	Het	7q36 deletion (0.16Mb)	nonsyndromic	metopic synostosis	20683987	2010
8p and 16q microduplication		compound Het	8p11.22 q12.1 and 16q11.2 q21,	syndromic	dolichocephaly	29245229	2017
8q13.3 deletion		Het	del(8)(q13.3q22.1)	syndromic	lamboid synostosis	7681252	1993
9p deletion			9p24.3 (see detail on MS)	9p deletion syndrome	trigonocephaly	34609792	2021
9p deletion		Het	9p, terminal 9.17Mb deletion	9p monosomy syndrome	metopic synostosis (trigonocephaly)	23480358	2013
9p deletion	*FREM1*	Het		nonsyndromic	metopic synostosis (trigonocephaly)	21931569	2011
9p13.2-p13.13 deletion	*KLF1*	Het		syndromic		27701781	2017
9p13 deletion i	*GALT* and *IL11RA*	homo	8.5Kb deletion on 9p13	syndromic (Cypriot with galactosemia)		24002815	2014
9p deletion		Het	18.74 Mb deletion of 9p24.3-p22.1 arr	9p deletion syndrome	trigonocephaly	28040136	2016

			9p24.3p22.1 (1–18,743,296 bp) × 1				
9p deletion		Het	11.78 Mb deletion of 9p24.3-p23 arr 9p24.3p23 (271,257–12,048,612 bp) × 1	9p deletion syndrome	trigonocephaly	28040136	2016
9p deletion				9p deletion syndrome	trigonocephaly	26597681	2016
9p deletion		Het	terminal deletion of 9p-del(9)(p22)	9p syndrome	metopic synostosis	7825591	1995
9p deletion		Het	9p delation	9p deletion syndrome	trigonocephaly	18452192	2008
9p deletion		Het		9p deletion syndrome	trigonocephaly	6846405	1983
9p deletion				syndromic	trigonocephaly	4541805	1973
9p22 deletion		Het	del(9)(p22)	partial 9p deletion syndrome	metopic synostosis (trigonocephaly)	21939171	2010
9p22 deletion		Het	del(9)(p22)	partial 9p deletion syndrome	metopic and partial coronal synostosis (trigonocephaly)	21939171	2010
9p22 deletion			46,XX,del(9)(p22)	syndromic	trigonocephaly	730163	1978
9pterp22 deletion			46,del(9) (pter → p22)	syndromic	trigonocephaly	BIRTH DEFECTS ORIG. ARTIC. SER. - Volume 12, Issue 5, pp. 151-155	1976
9p22 deletion		Het	del(9)(p22)	9p deletion syndrome	trigonocephaly	903150	1977
9q22 deletion	*ROR2* and *ECM2*	Het	9q22 deletion (3.92MB)	nonsyndromic	metopic synostosis	20683987	2010
9p22-p24 deletion		Het		syndromic	trigonocephaly	15857417	2005
9pterp23 deletion		Het	11.1-Mb 9pter-p23 deletion	syndromic	metopic synostosis (trigonocephaly)	19396833	2009
9p24 deletion		Het	9p24.3p24.1 (204,090–7, 149, 842)	syndromic with autism spectrum disorder (ASD)	trigonocephaly	34504731	2021
9p24.3 deletion	*DOCK8* and *KANK1*	Het	9p24.3 deletion	syndromic	metopic synostosis (trigonocephaly)	26656975	2016
9p monosomy			9p terminal deletion	syndromic		1084115	1976
9p monosomy			del(9)(qter leads to p22:)	syndromic		308344	1978
monosomy 9pter-->p22, trisomy 10q26--> qter			monosomy 9pter-->p22, trisomy 10q26--> qter	syndromic	trigonocephaly and closed fontanels	22303803	2011
9q21 deletion	*TMC1*	Het	9q21 deletion (0.04Mb)	nonsyndromic	sagittal synostosis	20683987	2010
9q22.3 deletion			9q22.3 microdeletion	9q22.3 microdeletion nevoid basal cell carcinoma syndrome (NBCCS)	brachycephaly	32028043	2020
translocation of 9q31.2 to 13q22.1	*ZNF462* and *KLF12*	Het	t(9; 13)(q31.2; q22.1)	syndromic	metopic synostosis	31361404	2019
translocation of 9q31.2 to 13q22.1		compound Het	t(9; 13)(q31.2; q22.1)	syndromic	metopic synostosis (trigonocephaly)	29427787	2018
9q22.3 deletion	*PTCH1*	Het	9q22.3 microdeletion	syndromic	metopic synostosis	221920277	2012
9q22 deletion	*ROR2* and *ECM2*	Het	9q22 deletion (3.92MB)	nonsyndromic	metopic synostosis	20683987	2010
translocation of 9q33 to 11p15	*SOX6*	Het	t(9;11)(q33.1;p15.3)	syndromic	lamboid and distal sagittal synostosis (brachycephaly)	16258006	2006
10p13 deletion			46,XX,del(10)p13	syndromic	trigonocephaly and brachycephaly	1150232	1975
10p15.3 deletion		Het	2.7 Mb deletion of 10p15.3	10p15.3 microdeletion syndrome	partial bicoronal synostosis (brachycephaly)	24357427	2014

10p15.3 deletion		Het	10p15.3 (171,237–2,880,776) 2.7Mb deletion	syndromic	partial bicoronal synostosis (brachycephaly)	24357427	2014
11p11.2 deletion	*EXT2, ALX4,* and *PHA21F*	Het	8.6 Mb deletion at 11p11.2 [arr 11p12p11.2 (Chr11:39,204,770-47,791,278)×1]	Potocki-Shaffer syndrome (PSS)	coornal synostosis	25653495	2015
11q11-q13.3 duplication	*FGF3* and *FGF4*	Het	dup(11)(q11-->q13.3)(29)/46,XY(6)	syndromic	sagittal and metopic and bilambdoid and temporal and squamosal synostosis (turri-, scapho-, and trigonocephaly)	17632770	2007
11q11-q13.3 duplication	*FGF3* and *FGF4*	Het	dup(11)(q13.5→q21).ish dup(11)(q13.5→q21)(wcp11+)mat	syndromic	trigonocephaly	17632770	2007
11q13 dupulication	*FGF3* and *FGF4*	Het	11q13.3, 290Kb microduplication	syndromic	prominent metopioc suture (plagiocephaly at 25month and pansynostosis at 8y.	24120895	2014
11q14.1-q22.3 deletion		Het	11q14.1-q22.3 deletion	11q14.1-q22.3 syndrome with neuroblastoma	prominence of right temporo-parietal suture and trigonocephaly	24035971	2013
11q23.3q25 deletion		Het	11q23.3q25, 14.2Mb delation	Jacobsen syndrome (JBS)	sagittal synostosis (dolicocephaly)	26997943	2016
11q25 duplication	*SNX19*	Het	11q25 duplication (0.45Mb)	nonsyndromic	coronal synostosis	20683987	2010
11q23-q24 deletion		Het		syndromic	trigonocephaly	15857417	2005
11q23 deletion			del(11)(q23->qter).	syndromic	sagittal synostosis	7205433	1980
11q23 deletion		Het	11q23 deletion	syndromic	trigonocephaly	24827952	2014
11q23.3 deletion		Het	del(11)(q23.3----qter)	syndromic	trigonocephaly	2301470	1990
11q23q25 deletion			46XX del(11) (q23-q25)	11q syndrome	trigonocephaly	6499268	1984
11q24.2q24.3 deletion		Het	46,XX,del(11)(q24.2–q24.3)	Jacobsen syndrome (JS)	sagittal synostosis	22965935	2012
11q deletion		Het		syndromic	trigonocephaly	6971620	1981
-11 and, der(11), translocation of 7p11.1 to 11p15.5			-11,+der(11),t(7;11)(p11.1;p15.5)mat	syndromic	lamboid synostosis	3631140	1987
12p12 deletion	*RERGL*	Het	12p12 deletion (0.08Mb)	nonsyndromic	sagittal synostosis	20683987	2010
fragile site 12q13			Fra(12)(q13)	Craniofrontonasal dysplasia	coornal synostosis (brachycephaly)	3742859	1986
trisomy 13		Het	pertial trisomy13; der(8)t(8;13)(p23;q14)	pertial trisomy 13q syndrome	metopic synostosis (trigonocephaly)	30936921	2019
trisomy 13		Het	free trisomy 47, XX+13	syndromic (Atelencephalic aprosencephaly)		Embase only: Pteridines - Volume 26, Issue 1, pp. 37-40	2015
trisomy 13		Het	der(7)t(7;13)(p22;q21)	Opitz Trigonocephaly C syndrome -like	trigonocephaly	Embase only: Int. J. Hum. Gen. - Volume 14, Issue 2, pp. 67-71	2014
trisomy 13				syndromic	trigonocephaly	933114	1976
trisomy 13q22			partial trisomy 13q22----qter	syndromic		2575360	1989

13q monosomy and translocation of 13q34 to 15q26.1	*IGF1R*	Het	der(13),t(13;15)(q34;q26.1)	syndromic with postnatal overgrowth	sagittal synostosis	12407708	2002
13q14 duplication and translocation of 10q to 13q14		Het	t(10;13)(qtr;q14)	syndromic	metopic synosotsis (trigonocephaly)	7471511	1981
13q distal trisomy and translocation of 9p12 to 13q21			t(9;13)(p21;q21)	syndromic	trigonocephaly	964983	1976
trisomy 13 and translocation of 3q24 to 13q14		Het	47,XY, +der(13),t(3,13)(q24;q14)	syndromic with Kleeblattschadel anomaly	right coronal synostosis (dolichocephaly)	7398112	1980
14q22 deletion		Het	del(14)(q22.1q23.2)	syndromic with anophthalmia and pituitary hypoplasia	lamboid synostosis (plagiocephaly)	9605291	1998
14q11.2q13.1 deletion		Het	14q11.2q13.1	syndromic	coornal synostosis	15216407	2004
14q32.3 mosaic deletion		Het	mos46,XX/46,XX,del[14][q32.3]=36%:64%	syndromic	coronal synostosis (brachycephaly)	1497515	1992
14q trisomy and translocation of 14p to 14q and 16p, to 16q			46,XX,t(14; 16)(14pter →14q11 :: 16q24→ 16qter; 16pter-→16q24 :: 14q11 →14qter).	syndromic	sagittal synostosis (scaphocephaly)	964994	1976
15q25 tetrasomy		Het	germline mosaicism of 15q25-qter	Shprintzen-Goldberg syndrome (SGS)		22639450	2012
15q25 tetrasomy		Het	mosaic tetrasomy 15q25-->qter	syndromic	metopic synostosis	8737657	1996
15q25.3 tetrasomy		Het	der(15)(qter!q25.3::q25.3→[neocen]→qter) (wcp15þ, D15Z-, SNRPN-, PML-,FES++)	Shprintzen-Goldberg syndrome (SGS) with Wilms tumor	CS	12400070	2002
15q25qter trisomy and 13qter monosomy		Het	der(13),t(13;15)(q34;q25.1)	15q25-qter trisomy syndrome	sagittal synostosis	10594876	1999
15q25-qter trisomy and 13qter monosomy		Het	der(13),t(13;15)(q34;q25.1)	15q25-qter trisomy syndrome	sagittal and coronal synostosis	10594876	1999
15q26 tetrasomy		Het	15q25.3→q26.3 (16.1 Mb	Shprintzen-Goldberg syndrome (SGS)-like	bicoronal synostosis (turricephaly)	22653535	2012
15q15.2-q22.2 deletion		Het	15q15.2-q22.2, 17.7Mb deletion	syndromic	bicoronal and metopic and sagittal synostosis	18449934	2008
duplication of 15q26.2→q26.3		Het	dup(15)(q26.2q26.3)	15q overgrowth syndrome	metopic and coronal synostosis	22030053	2011
15q deletion			15q [46,XY,del(15)(q15q22.1)]			2368808	1990
15q22.1 deletion	*FBN1*		15q22.1 deletion	Shprintzen-Goldberg syndrome with heart disease	coronal synostosis and thining sagittal suture	15840802	2005
16p deletion		Het	16p13.11-p12.3 2.6Mb microdeletion	syndromic	sagittal synostosis (dolicocephaly)	31205052	2019
16p13.11 and 19p13.3 deletion		Het	16p13.11 and 19p13.3 microdeletion	syndromic	lamboid synostosis	27751966	2016
16p13.11 duplication		Het		syndromic	sagittal and metopic synostosis or metopic synostosis	21150890	2011
16q23.1-16q23.3 deletion		Het	6.27Mb 16q23.1-16q23.3 deletion	syndromic	metipic synostosis (trigonocephaly)	24326958	2013
16q trisomy			46,XY,der(13)t(13qter→13p12:16q13→16qter)	syndromic		ACTA MED. AUXOL. - Volume 11, Issue	1979

						3, pp. 171-174	
17q21.31 deletion		Het	17q21.31 deletion	17q21.31 deletion syndrome		26424144	2015
trisomy 17q and monosomy 20q		compound Het	2.1 Mb duplication of 17q and 1.4 Mb deletion of 20q. 46,XY,der(20)t(17;20)(q25.3;q13.33)	syndromic	sagittal synostosis	25424318	2015
17q24q25.1 duplication		Het	dup(17)(q24q25.1)	syndromic		9689989	1998
17q25 deletion	*WDR45L* and *TBCD*	Het	17q25 deletion (0.6Mb)	nonsyndromic	metopic synostosis	20683987	2010
17q deletion		Het	del(17)(q23.1q24.2)	Hunter-McAlpine syndrome	coronal and metopic and lambdoid synosotsis	8723067	1996
trisomy 18		Het	trysomy 18	syndromic	trigonocephaly	33769585	2022
trisomy 18p and 2q13 deletion	*RGPD5, RGPD6, LIMS3,* and *LIMS3-LOC440895*		mos 47,XY+mar[34]/46, XY[31] and teisomy 18p11.21-18pter			25617521	2015
ring chromosom 18			mosaic chomosom	syndromic		32972420	2020
19q13.2 deletion	*ERF* and *CIC*	Het	deletions (63.7-583.2 kb)	syndromic		34117072	2021
19q deletion	*ERF*	Het		syndromic	saggital and metopic and lilamboid synostosis	33993607	2021
19q deletion	*ERF*	Het		syndromic	sagittal synostosis	33993607	2021
19q13.2q13.31 deletion		Het	1.6Mb 19q13.2q13.31 deletion	syndromic with Diamond-Blackfan anemia		27486481	2016
19p13.2–p13.13 deletion		Het	19p13.2–p13.13, 3Mb deletion	syndromic	left coronal and lambdoid and parieto-temporal synstosis	19842200	2009
19q32.2 deletion	*ERF*		19q32.2 microdeletion	syndromic (craniosynostosis-4)	coronal and metopic and lambdoid and sagittal synostosis	10.4038/sljch.v51i3.10260	2022
20q11.2 duplication	*ASXL1*	Het	multiple 20q11.2 microduplication	syndromic	metopic ridge (trigonocephaly)	23704076	2013
22q11.2 deletion		Het	22q11.2 deletion	22q11.2 deletion syndrome	bicoronal synostosis (turribrachycephaly)	23813949	2013
22q11.2 deletion		Het	2.68 Mb deletion at 22q11.21	22q11.2 deletion syndrome	metopic and bicoronal and bilamboid synostosis (cloverleaf skull)	23239640	2013
22q11.2 deletion		Het	22q11.2, 3Mb deletion	22q11.2 deletion syndrome	metipic synostosis (trigonocephaly)	16691595	2006
22q11.2 deletion	*FGFGR1,2, 3* and *TWIST1*	Het	22q11.2 deletion	22q11.2 deletion syndrome	bicoronal synostosis	16001439	2005
22q11.2 deletion		Het	22q11.2 deletion	22q11.2 deletion syndrome	unicoronal synostosis	16001439	2005
22q11 deletion		Het	22q11 deletion	syndromic		15658627	2004
22q11 deletion		Het		22q11deletion syndrome		8574419	1995
Xp11-q13.1 mosaic	*EFNB1*	Het	mosaicism 47,XY,+r(X)(p11.1q13)[7]/46,XY[23] (13.33 Mb gain at Xp11.21-q13.1 (55,260,049–68,590,017 bp))	Craniofrontonasal syndrome (CFNS)	brachycephaly	23614707	2014

Xp22.11 deletion	*ZFX, PDK3,* and *PCYT1B*	Het	Xp22.11, 300Kb deletion	syndromic		22052819	2011
Xp22.31 duplication		Het	Xp22.31(6,552,712 -8,097,511)×2.	syndromic	metopic ridge (trigonocephaly)	30733660	2019
Xp22			DXS7107–DXS989	Craniofrontonasal syndrome (CFNS)		10450866	1999
Xp22			?	Craniofrontonasal syndrome (CFNS)	coronal synostosis	10450866	1999
Xq12-q22.1 inactivation		homo		FG syndrome	turricephaly/ sagittal synostosis (dolichocephaly)	9805132	1998
Xq13.1-q21.1 duplication		Het	Xq13.1-q21.1 duplication	syndromic	sagittal synostosis	23974867	2014
Xq28 and 15qll.2ql3.l1 duplication		Het	Xq28 and 15qll.2ql3.l1	MECP2 duplication syndrome	compund synostosis (plagiochephaly)	30226965	2016
45X/46,XX/47,XX with mosaicism at chr18			45X/46,XX/47,XX with mosaicism at Chr18	syndromic with holoprosencephaly	pansynostosis	30024536	2018
47XXY			47,XXY	Prader-Willi syndrome with Kleinfelter syndrome	coronal synostosis	16791374	2006

Table S4. KEGG analysis.

ALL Genes					
Enrichment FDR	Number of Genes	Pathway Genes	Fold Enrichment	Pathway	Genes
1.91E-09	21	529	6.548123718	Path:hsa05200 Pathways in cancer	*CTNNB1, JAG1, FGF9, FGFR1, FGFR3, FGFR2, FGFR4, GLI3, GNAS, IGF1R, KRAS, LRP5, NOTCH1, NOTCH2, PTCH1, SHH, BMP2, TGFBR1, TGFBR2, AXIN2, CBL*
1.77E-07	11	148	12.25982365	Path:hsa05224 Breast cancer	*CTNNB1, JAG1, FGF9, FGFR1, IGF1R, KRAS, SHC4, LRP5, NOTCH1, NOTCH2, AXIN2*
2.87E-07	12	203	9.750759878	Path:hsa05205 Proteoglycans in cancer	*CTNNB1, FGFR1, FLNA, GPC3, IGF1R, KRAS, PPP1CB, PTCH1, ACTB, SHH, TWIST1, CBL*
9.58E-07	10	144	11.45488574	Path:hsa04550 Signaling pathways regulating pluripotency of stem cells	*CTNNB1, FGFR1, FGFR3, FGFR2, FGFR4, IGF1R, KRAS, TBX3, KAT6A, AXIN2*
1.06E-06	10	149	11.0704936	Path:hsa05226 Gastric cancer	*CTNNB1, FGF9, FGFR2, KRAS, SHC4, LRP5, SHH, TGFBR1, TGFBR2, AXIN2*
1.46E-06	10	157	10.50639201	Path:hsa04390 Hippo signaling pathway	*CTNNB1, DLG1, PPP1CB, PPP2R1A, ACTB, BMP2, BMP7, TGFBR1, TGFBR2, AXIN2*
2.03E-06	11	211	8.599307586	Path:hsa04015 Rap1 signaling pathway	*CTNNB1, EFNA4, FGF9, FGFR1, FGFR3, FGFR2, FGFR4, GNAS, IGF1R, KRAS, ACTB*
7.53E-06	8	107	12.33273679	Path:hsa04350 TGF-beta signaling pathway	*FBN1, LTBP1, PPP2R1A, SKI, BMP2, BMP7, TGFBR1, TGFBR2*
2.04E-05	9	168	8.83662614	Path:hsa05225 Hepatocellular carcinoma	*CTNNB1, IGF1R, KRAS, SHC4, LRP5, ACTB, TGFBR1, TGFBR2, AXIN2*
3.60E-05	7	96	12.02763002	Path:hsa01522 Endocrine resistance	*JAG1, GNAS, IGF1R, KRAS, SHC4, NOTCH1, NOTCH2*
3.60E-05	11	300	6.048179669	Path:hsa04010 MAPK signaling pathway	*EFNA4, FGF9, FGFR1, FGFR3, FGFR2, FGFR4, FLNA, IGF1R, KRAS, TGFBR1, TGFBR2*
3.60E-05	10	236	6.989421806	Path:hsa04014 Ras signaling pathway	*EFNA4, FGF9, FGFR1, FGFR3, FGFR2, FGFR4, IGF1R, KRAS, SHC4, SHOC2*
3.60E-05	6	63	15.70955758	Path:hsa05217 Basal cell carcinoma	*CTNNB1, GLI3, PTCH1, SHH, BMP2, AXIN2*
0.000177065	9	231	6.426637193	Path:hsa04810 Regulation of actin cytoskeleton	*DIAPH1, FGF9, FGFR1, FGFR3, FGFR2, FGFR4, KRAS, PPP1CB, ACTB*
0.000301419	6	93	10.64195836	Path:hsa04520 Adherens junction	*CTNNB1, FGFR1, IGF1R, ACTB, TGFBR1, TGFBR2*
0.000959385	10	362	4.55663963	Path:hsa04151 PI3K-Akt signaling pathway	*EFNA4, FGF9, FGFR1, FGFR3, FGFR2, FGFR4, IGF1R, KRAS, PCK1, PPP2R1A*
0.00111497	6	120	8.24751773	Path:hsa04919 Thyroid hormone signaling pathway	*CTNNB1, MED13L, KRAS, NOTCH1, NOTCH2, ACTB*
0.001135688	5	76	10.85199701	Path:hsa05220 Chronic myeloid leukemia	*KRAS, SHC4, TGFBR1, TGFBR2, CBL*
0.001248203	5	79	10.43989586	Path:hsa01521 EGFR tyrosine kinase inhibitor resistance	*FGFR3, FGFR2, IGF1R, KRAS, SHC4*
0.001248203	9	311	4.773482931	Path:hsa05206 MicroRNAs in cancer	*EFNA4, FGFR3, FOXP1, HNRNPK, KRAS, SHC4, NOTCH1, NOTCH2, ZEB2*
0.001845857	5	87	9.479905437	Path:hsa05210 Colorectal cancer	*CTNNB1, KRAS, TGFBR1, TGFBR2, AXIN2*
0.001849758	9	332	4.471545757	Path:hsa05165 Human papillomavirus infection	*CTNNB1, DLG1, JAG1, GNAS, KRAS, NOTCH1, NOTCH2, PPP2R1A, AXIN2*
0.002120737	7	202	5.716101397	Path:hsa04510 Focal adhesion	*CTNNB1, DIAPH1, FLNA, IGF1R, SHC4, PPP1CB, ACTB*
0.002683943	5	97	8.502595598	Path:hsa05215 Prostate cancer	*CTNNB1, FGFR1, FGFR2, IGF1R, KRAS*
0.00305244	4	56	11.78216819	Path:hsa04340 Hedgehog signaling pathway	*MEGF8, GLI3, PTCH1, SHH*
0.003586212	4	59	11.18307489	Path:hsa04730 Long-term depression	*GNAS, IGF1R, KRAS, PPP2R1A*
0.005006	5	114	7.234664676	Path:hsa04928 Parathyroid hormone synthesis secretion and action	*FGFR1, GNAS, LRP5, CASR, RUNX2*
0.006120179	7	250	4.618609929	Path:hsa04144 Endocytosis	*FGFR3, FGFR2, FGFR4, IGF1R, TGFBR1, TGFBR2, CBL*

0.006120179	6	182	5.437923778	Path:hsa04360 Axon guidance	*EFNA4, EFNB1, KRAS, PTCH1, SHH, BMP7*
0.006273219	4	71	9.292977725	Path:hsa05230 Central carbon metabolism in cancer	*FGFR1, FGFR3, FGFR2, KRAS*
0.006737378	4	73	9.038375595	Path:hsa05218 Melanoma	*FGF9, FGFR1, IGF1R, KRAS*
0.007366489	5	129	6.393424597	Path:hsa04926 Relaxin signaling pathway	*GNAS, KRAS, SHC4, TGFBR1, TGFBR2*
0.007864795	5	132	6.248119493	Path:hsa04068 FoxO signaling pathway	*IGF1R, KRAS, PCK1, TGFBR1, TGFBR2*
0.007864795	4	78	8.458992544	Path:hsa05100 Bacterial invasion of epithelial cells	*CTNNB1, SHC4, ACTB, CBL*
0.009082752	5	138	5.976462124	Path:hsa04910 Insulin signaling pathway	*KRAS, SHC4, PCK1, PPP1CB, CBL*
0.014260309	5	154	5.355530994	Path:hsa04934 Cushing syndrome	*CTNNB1, CYP17A1, GNAS, KMT2D, AXIN2*
0.01729594	4	99	6.664660792	Path:hsa04933 AGE-RAGE signaling pathway in diabetic complications	*DIAPH1, KRAS, TGFBR1, TGFBR2*
0.018748304	4	102	6.468641357	Path:hsa05142 Chagas disease	*GNAS, PPP2R1A, TGFBR1, TGFBR2*
0.020392644	3	52	9.516366612	Path:hsa04913 Ovarian steroidogenesis	*CYP17A1, GNAS, IGF1R*
0.022702723	6	252	3.927389395	Path:hsa04020 Calcium signaling pathway	*FGF9, FGFR1, FGFR3, FGFR2, FGFR4, GNAS*
0.027669273	3	59	8.387306167	Path:hsa05213 Endometrial cancer	*CTNNB1, KRAS, AXIN2*
0.028498243	5	188	4.386977516	Path:hsa05034 Alcoholism	*GNAS, KRAS, SHC4, PPP1CB, HDAC9*
0.028951891	3	61	8.112312522	Path:hsa04330 Notch signaling pathway	*JAG1, NOTCH1, NOTCH2*
0.030503215	4	122	5.408208348	Path:hsa04152 AMPK signaling pathway	*CPT1A, IGF1R, PCK1, PPP2R1A*
0.03797808	5	205	4.023179381	Path:hsa05203 Viral carcinogenesis	*DDX3X, DLG1, HNRNPK, KRAS, HDAC9*
0.041135876	3	71	6.969733293	Path:hsa04917 Prolactin signaling pathway	*CYP17A1, KRAS, SHC4*
0.043175043	4	138	4.781169699	Path:hsa04114 Oocyte meiosis	*IGF1R, PPP1CB, PPP2R1A, SMC1A*
0.043175043	5	215	3.836054758	Path:hsa05207 Chemical carcinogenesis-receptor activation	*JAG1, FGF9, GNAS, KRAS, NOTCH2*
0.044832479	3	75	6.598014184	Path:hsa05214 Glioma	*IGF1R, KRAS, SHC4*
0.047036689	5	223	3.698438444	Path:hsa05166 Human T-cell leukemia virus 1 infection	*DLG1, KRAS, MSX2, TGFBR1, TGFBR2*
0.047036689	3	77	6.426637193	Path:hsa05212 Pancreatic cancer	*KRAS, TGFBR1, TGFBR2*
0.048650606	5	226	3.649344129	Path:hsa04024 cAMP signaling pathway	*GLI3, GNAS, PPP1CB, PTCH1, TSHR*
0.052852658	3	83	5.96206101	Path:hsa03083 Polycomb repressive complex	*ASXL1, BCOR, ASXL3*
0.052852658	5	234	3.524580227	Path:hsa04714 Thermogenesis	*CPT1A, FGFR1, GNAS, KRAS, ACTB*
0.052852658	4	153	4.312427572	Path:hsa04921 Oxytocin signaling pathway	*GNAS, KRAS, PPP1CB, ACTB*
0.053221701	3	84	5.891084093	Path:hsa04012 ErbB signaling pathway	*KRAS, SHC4, CBL*
0.055570405	4	157	4.202556805	Path:hsa04218 Cellular senescence	*KRAS, PPP1CB, TGFBR1, TGFBR2*
0.055773985	4	158	4.175958345	Path:hsa04110 Cell cycle	*ESCO2, PPP2R1A, SMC1A, CDC45*
0.060767824	4	163	4.047861463	Path:hsa05161 Hepatitis B	*DDX3X, KRAS, TGFBR1, TGFBR2*
0.063225669	3	92	5.378815911	Path:hsa04658 Th1 and Th2 cell differentiation	*JAG1, NOTCH1, NOTCH2*
0.071108201	2	37	8.916235384	Path:hsa05216 Thyroid cancer	*CTNNB1, KRAS*
0.076093316	3	100	4.948510638	Path:hsa04916 Melanogenesis	*CTNNB1, GNAS, KRAS*
0.083451383	2	41	8.046358761	Path:hsa05219 Bladder cancer	*FGFR3, KRAS*
0.085689507	3	106	4.668406263	Path:hsa04922 Glucagon signaling pathway	*CPT1A, GNAS, PCK1*
0.090627232	3	109	4.539918017	Path:hsa04931 Insulin resistance	*CPT1A, PCK1, PPP1CB*
0.108551776	5	297	2.776941997	Path:hsa04060 Cytokine-cytokine receptor interaction	*IL11RA, BMP2, BMP7, TGFBR1, TGFBR2*
0.109069461	2	49	6.732667535	Path:hsa05110 Vibrio cholerae infection	*GNAS, ACTB*
0.110509705	3	120	4.123758865	Path:hsa04935 Growth hormone synthesis secretion and action	*GNAS, KRAS, SHC4*
0.111196435	3	121	4.089678213	Path:hsa04660 T cell receptor signaling pathway	*DLG1, KRAS, PPP2R1A*

0.116519953	3	124	3.990734386	Path:hsa04611 Platelet activation	*GNAS, PPP1CB, ACTB*
0.131606588	3	131	3.777489037	Path:hsa04728 Dopaminergic synapse	*GNAS, PPP1CB, PPP2R1A*
0.134698945	3	133	3.72068469	Path:hsa04380 Osteoclast differentiation	*CTSK, TGFBR1, TGFBR2*
0.139019396	3	137	3.612051561	Path:hsa04210 Apoptosis	*CTSK, KRAS, ACTB*
0.139019396	3	137	3.612051561	Path:hsa04371 Apelin signaling pathway	*JAG1, KRAS, TGFBR1*
0.139019396	2	59	5.591537444	Path:hsa04923 Regulation of lipolysis in adipocytes	*GNAS, TSHR*
0.139638697	3	138	3.585877274	Path:hsa04915 Estrogen signaling pathway	*GNAS, KRAS, SHC4*
0.14507899	2	62	5.320979181	Path:hsa04213 Longevity regulating pathway-multiple species	*IGF1R, KRAS*
0.145806655	3	142	3.484866647	Path:hsa04120 Ubiquitin mediated proteolysis	*HUWE1, TRIM37, CBL*
0.149589284	2	64	5.154698582	Path:hsa04927 Cortisol synthesis and secretion	*CYP17A1, GNAS*
0.154033822	3	147	3.366333768	Path:hsa04072 Phospholipase D signaling pathway	*GNAS, KRAS, SHC4*
0.154033822	2	66	4.998495594	Path:hsa04720 Long-term potentiation	*KRAS, PPP1CB*
0.163775296	3	153	3.234320679	Path:hsa04261 Adrenergic signaling in cardiomyocytes	*GNAS, PPP1CB, PPP2R1A*
0.163775296	2	69	4.781169699	Path:hsa05031 Amphetamine addiction	*GNAS, PPP1CB*
0.164662824	2	70	4.712867275	Path:hsa04920 Adipocytokine signaling pathway	*CPT1A, PCK1*
0.16742327	3	156	3.172122204	Path:hsa04150 mTOR signaling pathway	*IGF1R, KRAS, LRP5*
0.173046126	3	159	3.112270842	Path:hsa05160 Hepatitis C	*CTNNB1, KRAS, PPP2R1A*
0.175089776	2	74	4.458117692	Path:hsa04918 Thyroid hormone synthesis	*GNAS, TSHR*
0.177158656	2	75	4.398676123	Path:hsa04971 Gastric acid secretion	*GNAS, ACTB*
0.179210887	2	76	4.340798806	Path:hsa03320 PPAR signaling pathway	*CPT1A, PCK1*
0.190507641	3	169	2.928112804	Path:hsa04530 Tight junction	*DLG1, PPP2R1A, ACTB*
0.196444262	1	15	10.99669031	Path:hsa00730 Thiamine metabolism	*ALPL*
0.196633311	3	173	2.860410774	Path:hsa04310 Wnt signaling pathway	*CTNNB1, LRP5, AXIN2*

Syndromic Genes

Enrichment FDR	Number of Genes	Pathway Genes	Fold Enrichment	Pathway	Genes
0.000526277	4	96	33.41666667	Path:hsa01522 Endocrine resistance	*JAG1, IGF1R, SHC4, NOTCH1*
0.001220595	4	157	20.43312102	Path:hsa04390 Hippo signaling pathway	*DLG1, BMP2, BMP7, TGFBR2*
0.001220595	4	148	21.67567568	Path:hsa05224 Breast cancer	*JAG1, IGF1R, SHC4, NOTCH1*
0.0069753	3	107	22.48598131	Path:hsa04350 TGF-beta signaling pathway	*BMP2, BMP7, TGFBR2*
0.007945969	5	529	7.580340265	Path:hsa05200 Pathways in cancer	*JAG1, IGF1R, NOTCH1, BMP2, TGFBR2*
0.008596014	3	132	18.22727273	Path:hsa04068 FoxO signaling pathway	*IGF1R, PCK1, TGFBR2*
0.01481235	3	168	14.32142857	Path:hsa05225 Hepatocellular carcinoma	*IGF1R, SHC4, TGFBR2*
0.019865325	3	195	12.33846154	Path:hsa05202 Transcriptional misregulation in cancer	*IGF1R, TGFBR2, RUNX2*
0.025663666	2	61	26.29508197	Path:hsa04330 Notch signaling pathway	*JAG1, NOTCH1*
0.031959094	2	79	20.30379747	Path:hsa01521 EGFR tyrosine kinase inhibitor resistance	*IGF1R, SHC4*
0.031959094	2	75	21.38666667	Path:hsa05214 Glioma	*IGF1R, SHC4*
0.031959094	2	76	21.10526316	Path:hsa05220 Chronic myeloid leukemia	*SHC4, TGFBR2*
0.035119763	3	297	8.101010101	Path:hsa04060 Cytokine-cytokine receptor interaction	*BMP2, BMP7, TGFBR2*
0.035119763	2	93	17.24731183	Path:hsa04520 Adherens junction	*IGF1R, TGFBR2*

0.035119763	2	92	17.43478261	Path:hsa04658 Th1 and Th2 cell differentiation	*JAG1, NOTCH1*
0.044009943	3	332	7.246987952	Path:hsa05165 Human papillomavirus infection	*DLG1, JAG1, NOTCH1*
0.052285843	2	122	13.14754098	Path:hsa04152 AMPK signaling pathway	*IGF1R, PCK1*
0.054938944	2	129	12.43410853	Path:hsa04926 Relaxin signaling pathway	*SHC4, TGFBR2*
0.059183286	2	138	11.62318841	Path:hsa04910 Insulin signaling pathway	*SHC4, PCK1*
0.065028949	2	149	10.76510067	Path:hsa05226 Gastric cancer	*SHC4, TGFBR2*
0.077718287	1	15	53.46666667	Path:hsa00730 Thiamine metabolism	*ALPL*
0.104519603	2	202	7.940594059	Path:hsa04510 Focal adhesion	*IGF1R, SHC4*
0.108284297	1	23	34.86956522	Path:hsa04964 Proximal tubule bicarbonate reclamation	*PCK1*
0.114962984	2	223	7.192825112	Path:hsa05166 Human T-cell leukemia virus 1 infection	*DLG1, TGFBR2*
0.11771008	1	28	28.64285714	Path:hsa00790 Folate biosynthesis	*ALPL*
0.11771008	2	236	6.796610169	Path:hsa04014 Ras signaling pathway	*IGF1R, SHC4*
0.119811992	1	30	26.73333333	Path:hsa00020 Citrate cycle (TCA cycle)	*PCK1*
0.121382922	2	250	6.416	Path:hsa04144 Endocytosis	*IGF1R, TGFBR2*
0.162111782	1	47	17.06382979	Path:hsa00620 Pyruvate metabolism	*PCK1*
0.162111782	2	300	5.346666667	Path:hsa04010 MAPK signaling pathway	*IGF1R, TGFBR2*
0.162111782	2	311	5.15755627	Path:hsa05206 MicroRNAs in cancer	*SHC4, NOTCH1*
0.172934707	1	52	15.42307692	Path:hsa04913 Ovarian steroidogenesis	*IGF1R*
0.185019742	2	362	4.430939227	Path:hsa04151 PI3K-Akt signaling pathway	*IGF1R, PCK1*
0.185019742	1	62	12.93548387	Path:hsa04213 Longevity regulating pathway-multiple species	*IGF1R*
0.185019742	1	59	13.59322034	Path:hsa04730 Long-term depression	*IGF1R*
0.185019742	1	63	12.73015873	Path:hsa05217 Basal cell carcinoma	*BMP2*
0.190077332	1	67	11.97014925	Path:hsa00010 Glycolysis/Gluconeogenesis	*PCK1*
0.190077332	1	76	10.55263158	Path:hsa03320 PPAR signaling pathway	*PCK1*
0.190077332	1	71	11.29577465	Path:hsa04917 Prolactin signaling pathway	*SHC4*
0.190077332	1	70	11.45714286	Path:hsa04920 Adipocytokine signaling pathway	*PCK1*
0.190077332	1	78	10.28205128	Path:hsa05100 Bacterial invasion of epithelial cells	*SHC4*
0.190077332	1	77	10.41558442	Path:hsa05212 Pancreatic cancer	*TGFBR2*
0.190077332	1	73	10.98630137	Path:hsa05218 Melanoma	*IGF1R*
0.194819994	1	82	9.780487805	Path:hsa04623 Cytosolic DNA-sensing pathway	*ZBP1*
0.194905224	1	84	9.547619048	Path:hsa04012 ErbB signaling pathway	*SHC4*
0.19507973	1	90	8.911111111	Path:hsa04211 Longevity regulating pathway	*IGF1R*
0.19507973	1	89	9.011235955	Path:hsa04512 ECM-receptor interaction	*FREM1*
0.19507973	1	87	9.218390805	Path:hsa05210 Colorectal cancer	*TGFBR2*
0.199362695	1	109	7.357798165	Path:hsa04066 HIF-1 signaling pathway	*IGF1R*
0.199362695	1	107	7.495327103	Path:hsa04659 Th17 cell differentiation	*TGFBR2*
0.199362695	1	121	6.628099174	Path:hsa04660 T cell receptor signaling pathway	*DLG1*
0.199362695	1	119	6.739495798	Path:hsa04668 TNF signaling pathway	*JAG1*
0.199362695	1	119	6.739495798	Path:hsa04722 Neurotrophin signaling pathway	*SHC4*
0.199362695	1	110	7.290909091	Path:hsa04914 Progesterone-mediated oocyte maturation	*IGF1R*

0.199362695	1	120	6.683333333	Path:hsa04919 Thyroid hormone signaling pathway	*NOTCH1*
0.199362695	1	106	7.566037736	Path:hsa04922 Glucagon signaling pathway	*PCK1*
0.199362695	1	114	7.035087719	Path:hsa04928 Parathyroid hormone synthesis secretion and action	*RUNX2*
0.199362695	1	109	7.357798165	Path:hsa04931 Insulin resistance	*PCK1*
0.199362695	1	99	8.101010101	Path:hsa04933 AGE-RAGE signaling pathway in diabetic complications	*TGFBR2*
0.199362695	1	120	6.683333333	Path:hsa04935 Growth hormone synthesis secretion and action	*SHC4*
0.199362695	1	102	7.862745098	Path:hsa05142 Chagas disease	*TGFBR2*
0.199362695	1	97	8.268041237	Path:hsa05215 Prostate cancer	*IGF1R*

Nonsyndromic Genes

Enrichment FDR	Number of Genes	Pathway Genes	Fold Enrichment	Pathway	Genes
0.000526277	4	96	33.41666667	Path:hsa01522 Endocrine resistance	*JAG1, IGF1R, SHC4, NOTCH1*
0.001220595	4	157	20.43312102	Path:hsa04390 Hippo signaling pathway	*DLG1, BMP2, BMP7, TGFBR2*
0.001220595	4	148	21.67567568	Path:hsa05224 Breast cancer	*JAG1, IGF1R, SHC4, NOTCH1*
0.0069753	3	107	22.48598131	Path:hsa04350 TGF-beta signaling pathway	*BMP2, BMP7, TGFBR2*
0.007945969	5	529	7.580340265	Path:hsa05200 Pathways in cancer	*JAG1, IGF1R, NOTCH1, BMP2, TGFBR2*
0.008596014	3	132	18.22727273	Path:hsa04068 FoxO signaling pathway	*IGF1R, PCK1, TGFBR2*
0.01481235	3	168	14.32142857	Path:hsa05225 Hepatocellular carcinoma	*IGF1R, SHC4, TGFBR2*
0.019865325	3	195	12.33846154	Path:hsa05202 Transcriptional misregulation in cancer	*IGF1R, TGFBR2, RUNX2*
0.025663666	2	61	26.29508197	Path:hsa04330 Notch signaling pathway	*JAG1, NOTCH1*
0.031959094	2	79	20.30379747	Path:hsa01521 EGFR tyrosine kinase inhibitor resistance	*IGF1R, SHC4*
0.031959094	2	75	21.38666667	Path:hsa05214 Glioma	*IGF1R, SHC4*
0.031959094	2	76	21.10526316	Path:hsa05220 Chronic myeloid leukemia	*SHC4, TGFBR2*
0.035119763	3	297	8.101010101	Path:hsa04060 Cytokine-cytokine receptor interaction	*BMP2, BMP7, TGFBR2*
0.035119763	2	93	17.24731183	Path:hsa04520 Adherens junction	*IGF1R, TGFBR2*
0.035119763	2	92	17.43478261	Path:hsa04658 Th1 and Th2 cell differentiation	*JAG1, NOTCH1*
0.044009943	3	332	7.246987952	Path:hsa05165 Human papillomavirus infection	*DLG1, JAG1, NOTCH1*
0.052285843	2	122	13.14754098	Path:hsa04152 AMPK signaling pathway	*IGF1R, PCK1*
0.054938944	2	129	12.43410853	Path:hsa04926 Relaxin signaling pathway	*SHC4, TGFBR2*
0.059183286	2	138	11.62318841	Path:hsa04910 Insulin signaling pathway	*SHC4, PCK1*
0.065028949	2	149	10.76510067	Path:hsa05226 Gastric cancer	*SHC4, TGFBR2*
0.077718287	1	15	53.46666667	Path:hsa00730 Thiamine metabolism	*ALPL*
0.104519603	2	202	7.940594059	Path:hsa04510 Focal adhesion	*IGF1R, SHC4*
0.108284297	1	23	34.86956522	Path:hsa04964 Proximal tubule bicarbonate reclamation	*PCK1*
0.114962984	2	223	7.192825112	Path:hsa05166 Human T-cell leukemia virus 1 infection	*DLG1, TGFBR2*
0.11771008	1	28	28.64285714	Path:hsa00790 Folate biosynthesis	*ALPL*
0.11771008	2	236	6.796610169	Path:hsa04014 Ras signaling pathway	*IGF1R, SHC4*
0.119811992	1	30	26.73333333	Path:hsa00020 Citrate cycle (TCA cycle)	*PCK1*
0.121382922	2	250	6.416	Path:hsa04144 Endocytosis	*IGF1R, TGFBR2*
0.162111782	1	47	17.06382979	Path:hsa00620 Pyruvate metabolism	*PCK1*

0.162111782	2	300	5.346666667	Path:hsa04010 MAPK signaling pathway	*IGF1R, TGFBR2*
0.162111782	2	311	5.15755627	Path:hsa05206 MicroRNAs in cancer	*SHC4, NOTCH1*
0.172934707	1	52	15.42307692	Path:hsa04913 Ovarian steroidogenesis	*IGF1R*
0.185019742	2	362	4.430939227	Path:hsa04151 PI3K-Akt signaling pathway	*IGF1R, PCK1*
0.185019742	1	62	12.93548387	Path:hsa04213 Longevity regulating pathway-multiple species	*IGF1R*
0.185019742	1	59	13.59322034	Path:hsa04730 Long-term depression	*IGF1R*
0.185019742	1	63	12.73015873	Path:hsa05217 Basal cell carcinoma	*BMP2*
0.190077332	1	67	11.97014925	Path:hsa00010 Glycolysis/Gluconeogenesis	*PCK1*
0.190077332	1	76	10.55263158	Path:hsa03320 PPAR signaling pathway	*PCK1*
0.190077332	1	71	11.29577465	Path:hsa04917 Prolactin signaling pathway	*SHC4*
0.190077332	1	70	11.45714286	Path:hsa04920 Adipocytokine signaling pathway	*PCK1*
0.190077332	1	78	10.28205128	Path:hsa05100 Bacterial invasion of epithelial cells	*SHC4*
0.190077332	1	77	10.41558442	Path:hsa05212 Pancreatic cancer	*TGFBR2*
0.190077332	1	73	10.98630137	Path:hsa05218 Melanoma	*IGF1R*
0.194819994	1	82	9.780487805	Path:hsa04623 Cytosolic DNA-sensing pathway	*ZBP1*
0.194905224	1	84	9.547619048	Path:hsa04012 ErbB signaling pathway	*SHC4*
0.19507973	1	90	8.911111111	Path:hsa04211 Longevity regulating pathway	*IGF1R*
0.19507973	1	89	9.011235955	Path:hsa04512 ECM-receptor interaction	*FREM1*
0.19507973	1	87	9.218390805	Path:hsa05210 Colorectal cancer	*TGFBR2*
0.199362695	1	109	7.357798165	Path:hsa04066 HIF-1 signaling pathway	*IGF1R*
0.199362695	1	107	7.495327103	Path:hsa04659 Th17 cell differentiation	*TGFBR2*
0.199362695	1	121	6.628099174	Path:hsa04660 T cell receptor signaling pathway	*DLG1*
0.199362695	1	119	6.739495798	Path:hsa04668 TNF signaling pathway	*JAG1*
0.199362695	1	119	6.739495798	Path:hsa04722 Neurotrophin signaling pathway	*SHC4*
0.199362695	1	110	7.290909091	Path:hsa04914 Progesterone-mediated oocyte maturation	*IGF1R*
0.199362695	1	120	6.683333333	Path:hsa04919 Thyroid hormone signaling pathway	*NOTCH1*
0.199362695	1	106	7.566037736	Path:hsa04922 Glucagon signaling pathway	*PCK1*
0.199362695	1	114	7.035087719	Path:hsa04928 Parathyroid hormone synthesis secretion and action	*RUNX2*
0.199362695	1	109	7.357798165	Path:hsa04931 Insulin resistance	*PCK1*
0.199362695	1	99	8.101010101	Path:hsa04933 AGE-RAGE signaling pathway in diabetic complications	*TGFBR2*
0.199362695	1	120	6.683333333	Path:hsa04935 Growth hormone synthesis secretion and action	*SHC4*
0.199362695	1	102	7.862745098	Path:hsa05142 Chagas disease	*TGFBR2*
0.199362695	1	97	8.268041237	Path:hsa05215 Prostate cancer	*IGF1R*

Table S5. GO Biological Process.

All Genes					
Enrichment FDR	Number of Genes	Pathway Genes	Fold Enrichment	Pathway	Genes
3.65E-28	40	556	11.86693199	GO:0001501 Skeletal system development	*RUNX2, GNAS, FOXP1, BMP2, FBN1, ALX4, FGFR2, DLG1, FGFR1, BMP7, FGF9, MEGF8, GLI3, TGFBR1, MSX2, TWIST1, FGFRL1, CTSK, MCPH1, WDR19, LRP5, ALPL, NFIA, TGFBR2, SHH, CTNNB1, AXIN2, ASXL1, FAM20C, IFT140, CYP26B1, RAB23, NOTCH2, TBX3, FREM1, CHD7, SH3PXD2B, FGFR3, SKI, TCOF1*
1.64E-25	48	1086	7.290623408	GO:0009887 Animal organ morphogenesis	*FGF9, ASXL3, CHD7, ASXL1, FAM20C, BMP2, AHDC1, TBX3, CYP26B1, ALX4, ABCC9, DLG1, FGFR1, BMP7, JAG1, MEGF8, GLI3, TGFBR1, MSX2, TWIST1, RUNX2, FGFRL1, KRAS, NOTCH2, GPC3, NOTCH1, WDR19, LRP5, ALPL, TGFBR2, SPRY1, SHH, TSHR, CTNNB1, AXIN2, PTCH1, IFT140, FLNA, FBN1, FGFR2, RAB23, FREM1, BCOR, FGFR3, ZIC1, SKI, IFT122, LHX3*
1.64E-25	25	158	26.09973965	GO:0035107 Appendage morphogenesis	*TBX3, CYP26B1, ALX4, FGFR1, BMP7, FGF9, MEGF8, GLI3, MSX2, TWIST1, RUNX2, NOTCH2, GPC3, NOTCH1, WDR19, LRP5, NFIA, SHH, CTNNB1, CHD7, PTCH1, IFT140, FGFR2, SKI, IFT122*
1.64E-25	25	158	26.09973965	GO:0035108 Limb morphogenesis	*TBX3, CYP26B1, ALX4, FGFR1, BMP7, FGF9, MEGF8, GLI3, MSX2, TWIST1, RUNX2, NOTCH2, GPC3, NOTCH1, WDR19, LRP5, NFIA, SHH, CTNNB1, CHD7, PTCH1, IFT140, FGFR2, SKI, IFT122*
6.89E-25	23	127	29.87289887	GO:0030326 Embryonic limb morphogenesis	*CYP26B1, ALX4, FGFR1, BMP7, FGF9, MEGF8, GLI3, MSX2, TWIST1, RUNX2, NOTCH2, TBX3, GPC3, NOTCH1, WDR19, LRP5, SHH, CTNNB1, CHD7, PTCH1, IFT140, SKI, IFT122*
6.89E-25	23	127	29.87289887	GO:0035113 Embryonic appendage morphogenesis	*CYP26B1, ALX4, FGFR1, BMP7, FGF9, MEGF8, GLI3, MSX2, TWIST1, RUNX2, NOTCH2, TBX3, GPC3, NOTCH1, WDR19, LRP5, SHH, CTNNB1, CHD7, PTCH1, IFT140, SKI, IFT122*
3.32E-23	25	199	20.72240636	GO:0048736 Appendage development	*TBX3, CYP26B1, ALX4, FGFR1, BMP7, FGF9, MEGF8, GLI3, MSX2, TWIST1, RUNX2, NOTCH2, GPC3, NOTCH1, WDR19, LRP5, NFIA, SHH, CTNNB1, CHD7, PTCH1, IFT140, FGFR2, SKI, IFT122*
3.32E-23	25	199	20.72240636	GO:0060173 Limb development	*TBX3, CYP26B1, ALX4, FGFR1, BMP7, FGF9, MEGF8, GLI3, MSX2, TWIST1, RUNX2, NOTCH2, GPC3, NOTCH1, WDR19, LRP5, NFIA, SHH, CTNNB1, CHD7, PTCH1, IFT140, FGFR2, SKI, IFT122*
1.43E-22	36	623	9.531641679	GO:0007507 Heart development	*TGFBR1, TGFBR2, CHD7, BMP2, TBX3, ABCC9, FGFR1, BMP7, JAG1, FGF9, CTCF, MEGF8, GLI3, FOXP1, MSX2, TWIST1, FGFRL1, NOTCH2, IGF1R, GPC3, NOTCH1, SKI, SPRY1, SHH, CTNNB1, AXIN2, ASXL1, PTCH1, IFT140, FLNA, FBN1, SH3PXD2B, BCOR, HDAC9, FGFR2, IFT122*
2.06E-22	71	3229	3.626966608	GO:0048513 Animal organ development	*ACTB, FGF9, TGFBR1, PCK1, RUNX2, ASXL3, TGFBR2, SPRY1, CHD7, ASXL1, FAM20C, GNAS, BMP7, FOXP1, BMP2, AHDC1, TBX3, ZIC1, SHH, NELL1, FBN1, CYP26B1, CASR, ALX4, SPO11, ASPM, FGFR2, ABCC9, DLG1, FGFR1, EFNB1, JAG1, CTCF, ERCC2, MEGF8, GLI3, LHX3, CPT1A, CBL, MSX2, TWIST1, FGFRL1, KRAS, NOTCH2, IGF1R, FTO, CTSK, GPC3, MCPH1, NOTCH1, WDR19, SKI, LRP5, ALPL, NFIA, BMPER, TSHR, CTNNB1, AXIN2, PTCH1, IFT140, FLNA, TCOF1, RAB23, FREM1, SH3PXD2B, BCOR, HDAC9, FGFR3, IFT122, TCF12*
3.45E-22	59	2165	4.495182874	GO:0009888 Tissue development	*PCK1, RUNX2, FAM20C, GNAS, BMP7, TGFBR1, BMP2, AHDC1, TBX3, TGFBR2, SHH, NELL1, FLNA, CYP26B1, CASR, FGFR2, ABCC9, DLG1, FGFR1, EFNB1, JAG1, FGF9, CTCF, ERCC2, MEGF8, GLI3, LHX3, FOXP1, MSX2, TWIST1, KRAS, NOTCH2, FTO, ADAMTSL4, CTSK, GPC3, NOTCH1, WDR19, SKI, LRP5, ALPL, NFIA, SPRY1, BMPER, CTNNB1, AXIN2, CHD7, ASXL1, PTCH1, IFT140, CPT1A, ALX4, TCOF1, ACTB, BCOR, HDAC9, FGFR3, IFT122, SH3PXD2B*
1.15E-20	65	2868	3.738414592	GO:0009653 Anatomical structure morphogenesis	*ACTB, EFNB1, FGF9, MSX2, NOTCH2, ASXL3, NOTCH1, BMPER, FREM1, FBN1, CHD7, ASXL1, FAM20C, EFNA4, GNAS, BMP7, BMP2, AHDC1, TBX3, FLNA, CYP26B1, CASR, ALX4, FGFR2, ABCC9, DLG1, FGFR1, JAG1, ERCC2, MEGF8, GLI3, TGFBR1, LHX3, CNOT2, TWIST1, RUNX2, FGFRL1, KRAS, IGF1R, GPC3, PTPRD, WDR19, LRP5, ALPL, NFIA, TGFBR2, SPRY1, SHH, TSHR, CTNNB1, AXIN2, PTCH1, IFT140, HDAC9, ADCK1, TCOF1, HUWE1, RAB23, FOXP1, DIAPH1, BCOR, FGFR3, ZIC1, SKI, IFT122*
1.58E-20	24	228	17.36319522	GO:0060348 Bone development	*GNAS, FOXP1, BMP2, FBN1, FGFR2, GLI3, MSX2, TWIST1, RUNX2, MCPH1, LRP5, ALPL, TGFBR2, AXIN2, ASXL1, FAM20C,*

					CYP26B1, MEGF8, RAB23, NOTCH2, FREM1, SH3PXD2B, FGFR3, SKI
1.03E-18	80	4782	2.75952078	GO:0030154 Cell differentiation	*ALPL, ACTB, FGFR1, OSTM1, EFNB1, FGF9, LHX3, PCK1, RUNX2, NOTCH2, TBX3, NOTCH1, SHH, NELL1, AXIN2, DDX3X, EFNA4, CETP, KAT6A, BMP7, CTCF, TGFBR1, FOXP1, BMP2, SKI, TGFBR2, FBN1, CYP26B1, HDAC9, SPO11, ASPM, FGFR2, HUWE1, JAG1, ERCC2, DMPK, MEGF8, PPP2R1A, GLI3, SHOC2, CNOT2, MSX2, BBS9, TWIST1, IFT81, CTCFL, FAM209A, AHDC1, RBM38, KRAS, TCF12, IGF1R, ADAMTSL4, CTSK, GPC3, ZIC1, PTPRD, SPAG17, LRP5, NFIA, SPRY1, TSHR, CTNNB1, PDILT, ZEB2, CHD7, ESCO2, ASXL1, SH3PXD2B, FAM20C, SHC4, PTCH1, IFT140, FLNA, CPT1A, TCOF1, FTO, KMT2D, KAT6B, FGFR3*
1.03E-18	23	240	15.80774232	GO:0048705 Skeletal system morphogenesis	*ALX4, DLG1, BMP7, MEGF8, GLI3, TGFBR1, MSX2, TWIST1, RUNX2, WDR19, ALPL, TGFBR2, CTNNB1, AXIN2, IFT140, CYP26B1, FGFR2, RAB23, LRP5, FREM1, FGFR3, SKI, FGFR1*
1.03E-18	80	4783	2.758943836	GO:0048869 Cellular developmental process	*ALPL, ACTB, FGFR1, OSTM1, EFNB1, FGF9, LHX3, PCK1, RUNX2, NOTCH2, TBX3, NOTCH1, SHH, NELL1, AXIN2, DDX3X, EFNA4, CETP, KAT6A, BMP7, CTCF, TGFBR1, FOXP1, BMP2, SKI, TGFBR2, FBN1, CYP26B1, HDAC9, SPO11, ASPM, FGFR2, HUWE1, JAG1, ERCC2, DMPK, MEGF8, PPP2R1A, GLI3, SHOC2, CNOT2, MSX2, BBS9, TWIST1, IFT81, CTCFL, FAM209A, AHDC1, RBM38, KRAS, TCF12, IGF1R, ADAMTSL4, CTSK, GPC3, ZIC1, PTPRD, SPAG17, LRP5, NFIA, SPRY1, TSHR, CTNNB1, PDILT, ZEB2, CHD7, ESCO2, ASXL1, SH3PXD2B, FAM20C, SHC4, PTCH1, IFT140, FLNA, CPT1A, TCOF1, FTO, KMT2D, KAT6B, FGFR3*
1.38E-18	47	1566	4.950617284	GO:0009719 Response to endogenous stimulus	*BMP7, FGF9, TGFBR1, PCK1, BMP2, POR, SKI, TGFBR2, SPRY1, TSHR, HDAC9, LTBP1, FGFR2, FGFR3, SMC1A, FGFR1, GNAS, FOXP1, NOTCH1, FGFR4, KMT2D, CYP26B1, CASR, ABCC9, MEGF8, SHOC2, CBL, MSX2, TWIST1, RUNX2, FGFRL1, NOTCH2, IGF1R, CTSK, GPC3, LRP5, ALPL, NFIA, BMPER, FBN1, CTNNB1, AXIN2, ASXL1, FAM20C, CNOT2, TCF12, ZEB2*
3.09E-18	29	491	9.742485303	GO:0007389 Pattern specification process	*BMP2, CYP26B1, ALX4, FGFR1, EFNB1, BMP7, MEGF8, GLI3, TGFBR1, LHX3, MSX2, NOTCH2, TBX3, GPC3, NOTCH1, ZIC1, WDR19, LRP5, TGFBR2, SPRY1, SHH, CTNNB1, AXIN2, PTCH1, IFT140, BCOR, FGFR2, SKI, IFT122*
7.26E-18	59	2666	3.650439206	GO:0050793 Regulation of developmental process	*FGFR1, CNOT2, RUNX2, BMPER, NELL1, CETP, BMP7, FOXP1, BMP2, NOTCH2, TBX3, NOTCH1, SKI, TGFBR2, SHH, FBN1, CYP26B1, ASPM, DLG1, JAG1, FGF9, DMPK, MEGF8, GLI3, TGFBR1, SHOC2, MSX2, TWIST1, PCK1, RBM38, KRAS, TCF12, IGF1R, FTO, CTSK, PTPRD, LRP5, SPRY1, TSHR, CTNNB1, AXIN2, CHD7, ASXL1, FAM20C, PTCH1, FLNA, ADCK1, HUWE1, DIAPH1, BCOR, HDAC9, FGFR2, FGFR3, GNAS, ZEB2, ACTB, KAT6A, PPP2R1A, KAT6B*
1.42E-17	42	1293	5.358016159	GO:0071495 Cellular response to endogenous stimulus	*BMP7, FGF9, TGFBR1, PCK1, BMP2, SKI, TGFBR2, SPRY1, TSHR, HDAC9, LTBP1, FGFR2, FGFR3, SMC1A, FGFR1, FOXP1, NOTCH1, FGFR4, CYP26B1, CASR, MEGF8, SHOC2, CBL, MSX2, TWIST1, RUNX2, FGFRL1, NOTCH2, IGF1R, CTSK, GPC3, NFIA, BMPER, FBN1, CTNNB1, AXIN2, ASXL1, FAM20C, CNOT2, KMT2D, ZEB2, GNAS*
2.45E-17	42	1314	5.272385764	GO:0060429 Epithelium development	*PCK1, GNAS, SHH, FLNA, CYP26B1, CASR, DLG1, FGFR1, BMP7, JAG1, ERCC2, MEGF8, GLI3, LHX3, MSX2, TWIST1, BMP2, KRAS, NOTCH2, TBX3, ADAMTSL4, GPC3, NOTCH1, WDR19, LRP5, TGFBR2, SPRY1, BMPER, CTNNB1, AXIN2, CHD7, ASXL1, FAM20C, PTCH1, IFT140, CPT1A, ALX4, ACTB, FOXP1, FGFR2, SKI, IFT122*
4.03E-17	31	645	7.927846501	GO:0007423 Sensory organ development	*CHD7, CYP26B1, DLG1, FGFR1, BMP7, JAG1, FGF9, GLI3, TGFBR1, BMP2, NOTCH2, TBX3, NOTCH1, WDR19, LRP5, NFIA, TGFBR2, SPRY1, BMPER, SHH, TSHR, CTNNB1, IFT140, LHX3, FBN1, TWIST1, SH3PXD2B, FGFR2, ZIC1, SKI, IFT122*
4.22E-17	40	1197	5.512125467	GO:0009790 Embryo development	*MSX2, CHD7, AHDC1, SHH, CYP26B1, ALX4, FGFR2, DLG1, FGFR1, EFNB1, BMP7, JAG1, FGF9, CTCF, ERCC2, MEGF8, GLI3, TGFBR1, CNOT2, TWIST1, RUNX2, BMP2, NOTCH2, TBX3, GPC3, NOTCH1, WDR19, LRP5, TGFBR2, TSHR, CTNNB1, AXIN2, BCOR, PTCH1, IFT140, FBN1, TCOF1, ZIC1, SKI, IFT122*
4.22E-17	31	648	7.891143508	GO:0048598 Embryonic morphogenesis	*MSX2, CHD7, AHDC1, CYP26B1, ALX4, FGFR2, DLG1, FGFR1, BMP7, FGF9, MEGF8, GLI3, TGFBR1, TWIST1, RUNX2, NOTCH2, TBX3, GPC3, NOTCH1, WDR19, LRP5, TGFBR2, SHH, TSHR, CTNNB1, PTCH1, IFT140, FBN1, ZIC1, SKI, IFT122*
1.84E-16	46	1703	4.45549989	GO:0045595 Regulation of cell differentiation	*FGFR1, RUNX2, NELL1, CETP, BMP7, FOXP1, BMP2, NOTCH2, TBX3, NOTCH1, SKI, TGFBR2, FBN1, CYP26B1, ASPM, JAG1, FGF9, DMPK, MEGF8, GLI3, TGFBR1, SHOC2, MSX2, TWIST1, PCK1, RBM38, KRAS, TCF12, PTPRD, LRP5, SPRY1, SHH,*

					CTNNB1, AXIN2, CHD7, ASXL1, FAM20C, PTCH1, FTO, HDAC9, FGFR2, ZEB2, ACTB, KAT6A, PPP2R1A, KAT6B
2.28E-16	22	275	13.19602837	GO:0003007 Heart morphogenesis	CHD7, BMP2, ABCC9, BMP7, JAG1, MEGF8, TGFBR1, MSX2, TWIST1, FGFRL1, NOTCH2, TBX3, NOTCH1, TGFBR2, SPRY1, SHH, CTNNB1, AXIN2, ASXL1, PTCH1, FLNA, FGFR2
2.38E-16	39	1192	5.396865629	GO:0072359 Circulatory system development	TGFBR1, TGFBR2, BMPER, CHD7, BMP7, BMP2, TBX3, NOTCH1, ABCC9, FGFR1, JAG1, FGF9, CTCF, MEGF8, GLI3, FOXP1, MSX2, TWIST1, FGFRL1, NOTCH2, IGF1R, GPC3, SKI, LRP5, SPRY1, SHH, CTNNB1, AXIN2, ANTXR1, ASXL1, PTCH1, IFT140, FLNA, HDAC9, FBN1, SH3PXD2B, BCOR, FGFR2, IFT122
2.52E-16	72	4287	2.770334857	GO:0048731 System development	ACTB, FGF9, EFNB1, TGFBR1, LHX3, PCK1, RUNX2, NOTCH2, NOTCH1, ZIC1, LRP5, TGFBR2, BMPER, SHH, CHD7, EFNA4, GNAS, BMP7, FOXP1, BMP2, TBX3, PTPRD, FBN1, CYP26B1, HDAC9, ALX4, SPO11, ASPM, FGFR2, ABCC9, DLG1, FGFR1, JAG1, CTCF, ERCC2, MEGF8, GLI3, SHOC2, CPT1A, CBL, MSX2, TWIST1, FGFRL1, KRAS, TCF12, IGF1R, CTSK, GPC3, MCPH1, WDR19, SKI, ALPL, NFIA, SPRY1, TSHR, CTNNB1, AXIN2, ZEB2, ANTXR1, ASXL1, FAM20C, PTCH1, IFT140, FLNA, RAB23, FREM1, SH3PXD2B, BCOR, FGFR3, IFT122, TCOF1, NELL1
2.83E-16	23	317	11.9680068	GO:0048562 Embryonic organ morphogenesis	CHD7, ALX4, DLG1, FGFR1, BMP7, FGF9, MEGF8, GLI3, TGFBR1, TWIST1, RUNX2, NOTCH2, TBX3, NOTCH1, WDR19, TGFBR2, SHH, TSHR, CTNNB1, IFT140, FBN1, FGFR2, ZIC1
3.92E-16	30	651	7.60139883	GO:0048729 Tissue morphogenesis	AHDC1, FLNA, CASR, FGFR2, DLG1, FGFR1, BMP7, JAG1, MEGF8, GLI3, TGFBR1, MSX2, TWIST1, BMP2, KRAS, NOTCH2, TBX3, GPC3, NOTCH1, LRP5, TGFBR2, SPRY1, SHH, CTNNB1, CHD7, PTCH1, ACTB, FOXP1, SKI, IFT122
5.06E-16	51	2185	3.85009981	GO:0008283 Cell population proliferation	FGF9, IL11RA, FGFR2, DLG1, FGFR1, BMP7, BMP2, RBM38, NOTCH2, TBX3, NOTCH1, SKI, FGFR4, LRP5, TGFBR2, BMPER, SHH, NELL1, CTNNB1, CASR, ASPM, FGFR3, ABCC9, EFNB1, JAG1, ERCC2, GLI3, TGFBR1, SHOC2, DPH1, MSX2, TWIST1, RUNX2, FGFRL1, KRAS, IGF1R, FTO, GPC3, NFIA, SPRY1, AXIN2, ZEB2, SHC4, PTCH1, FLNA, CTCF, FOXP1, DIS3L2, KMT2D, ACTB, TSHR
5.06E-16	38	1155	5.426938074	GO:0035295 Tube development	BMPER, NOTCH1, CASR, ALX4, DLG1, FGFR1, BMP7, JAG1, FGF9, MEGF8, GLI3, TGFBR1, LHX3, MSX2, TWIST1, BMP2, KRAS, NOTCH2, TBX3, GPC3, WDR19, LRP5, NFIA, TGFBR2, SPRY1, SHH, FBN1, CTNNB1, CHD7, ASXL1, PTCH1, IFT140, FLNA, HDAC9, FOXP1, FGFR2, SKI, IFT122
5.06E-16	31	715	7.151693696	GO:0071363 Cellular response to growth factor stimulus	BMP7, FGF9, TGFBR1, BMP2, SKI, TGFBR2, SPRY1, LTBP1, FGFR2, FGFR3, FGFR1, NOTCH1, FGFR4, CASR, MEGF8, SHOC2, CBL, MSX2, TWIST1, RUNX2, FGFRL1, NOTCH2, IGF1R, CTSK, GPC3, NFIA, BMPER, FBN1, CTNNB1, FAM20C, ZEB2
1.19E-15	60	3099	3.193617708	GO:0007166 Cell surface receptor signaling pathway	CASS4, EFNB1, BMP7, FGF9, GLI3, TGFBR1, TCTN3, ZBP1, BMP2, TRAF7, NOTCH2, IL11RA, IGF1R, GPC3, NOTCH1, SKI, LRP5, TGFBR2, SPRY1, SHH, CTNNB1, AXIN2, SHC4, PTCH1, EFNA4, LTBP1, FGFR2, FGFR3, FGFR1, FGFR4, BMPER, ASPM, JAG1, ERCC2, DMPK, MEGF8, PPP2R1A, SHOC2, CBL, MSX2, RUNX2, FGFRL1, PTH2R, ZIC1, PTPRD, WDR19, NFIA, FBN1, FAM20C, IFT140, FLNA, FOXP1, IFT81, TSHR, ZEB2, DDX3X, IFT122, COLEC11, COLEC10, P4HB
1.36E-15	30	685	7.224103122	GO:0043009 Chordate embryonic development	CHD7, ALX4, DLG1, FGFR1, BMP7, FGF9, CTCF, ERCC2, MEGF8, GLI3, TGFBR1, CNOT2, TWIST1, RUNX2, BMP2, NOTCH2, TBX3, NOTCH1, WDR19, TGFBR2, SHH, CTNNB1, AXIN2, BCOR, PTCH1, IFT140, FGFR2, TCOF1, SKI, IFT122
1.60E-15	31	747	6.845329308	GO:0070848 Response to growth factor	BMP7, FGF9, TGFBR1, BMP2, SKI, TGFBR2, SPRY1, LTBP1, FGFR2, FGFR3, FGFR1, NOTCH1, FGFR4, CASR, MEGF8, SHOC2, CBL, MSX2, TWIST1, RUNX2, FGFRL1, NOTCH2, IGF1R, CTSK, GPC3, NFIA, BMPER, FBN1, CTNNB1, FAM20C, ZEB2
1.81E-15	27	533	8.355834098	GO:0002009 Morphogenesis of an epithelium	FLNA, CASR, DLG1, FGFR1, BMP7, JAG1, MEGF8, GLI3, MSX2, TWIST1, BMP2, KRAS, NOTCH2, TBX3, GPC3, NOTCH1, LRP5, TGFBR2, SPRY1, SHH, CTNNB1, PTCH1, ACTB, FOXP1, FGFR2, SKI, IFT122
2.02E-15	14	75	30.79073286	GO:1904888 Cranial skeletal system development	GLI3, TGFBR1, MSX2, TWIST1, RUNX2, WDR19, TGFBR2, CTNNB1, IFT140, FGFR2, MEGF8, RAB23, FREM1, COLEC10
2.04E-15	26	487	8.806384435	GO:0048568 Embryonic organ development	CHD7, ALX4, DLG1, FGFR1, BMP7, FGF9, ERCC2, MEGF8, GLI3, TGFBR1, TWIST1, RUNX2, NOTCH2, TBX3, NOTCH1, WDR19, TGFBR2, SHH, TSHR, CTNNB1, PTCH1, IFT140, FBN1, FGFR2, ZIC1, IFT122
2.47E-15	59	3054	3.186663694	GO:0048468 Cell development	ACTB, OSTM1, EFNB1, RUNX2, NOTCH2, NOTCH1, AXIN2, EFNA4, KAT6A, BMP7, CTCF, FOXP1, TBX3, SHH, FBN1, CYP26B1, SPO11, ASPM, FGFR1, JAG1, ERCC2, MEGF8,

					PPP2R1A, GLI3, LHX3, SHOC2, MSX2, TWIST1, IFT81, CTCFL, PCK1, BMP2, KRAS, IGF1R, ADAMTSL4, CTSK, GPC3, PTPRD, SPAG17, LRP5, TGFBR2, SPRY1, TSHR, CTNNB1, PDILT, ZEB2, CHD7, ESCO2, ASXL1, FAM20C, PTCH1, IFT140, FLNA, SKI, TCOF1, HDAC9, FGFR2, KMT2D, KAT6B
2.59E-15	30	706	7.009221867	GO:0009792 Embryo development ending in birth or egg hatching	*CHD7, ALX4, DLG1, FGFR1, BMP7, FGF9, CTCF, ERCC2, MEGF8, GLI3, TGFBR1, CNOT2, TWIST1, RUNX2, BMP2, NOTCH2, TBX3, NOTCH1, WDR19, TGFBR2, SHH, CTNNB1, AXIN2, BCOR, PTCH1, IFT140, FGFR2, TCOF1, SKI, IFT122*
2.59E-15	23	356	10.65690493	GO:0060562 Epithelial tube morphogenesis	*CASR, DLG1, BMP7, MEGF8, GLI3, MSX2, TWIST1, BMP2, KRAS, NOTCH2, TBX3, GPC3, NOTCH1, LRP5, TGFBR2, SPRY1, SHH, CTNNB1, PTCH1, FOXP1, FGFR2, SKI, IFT122*
2.75E-15	25	447	9.225411332	GO:0003002 Regionalization	*BMP2, CYP26B1, ALX4, FGFR1, MEGF8, GLI3, TGFBR1, LHX3, MSX2, NOTCH2, TBX3, GPC3, NOTCH1, WDR19, LRP5, TGFBR2, SPRY1, SHH, CTNNB1, AXIN2, PTCH1, IFT140, FGFR2, SKI, IFT122*
3.87E-15	25	454	9.083169307	GO:0001503 Ossification	*ALPL, RUNX2, NELL1, BMP7, BMP2, SKI, CASR, JAG1, FGF9, ERCC2, GLI3, MSX2, TWIST1, CTSK, GPC3, NOTCH1, LRP5, SHH, CTNNB1, AXIN2, FAM20C, BCOR, PTCH1, FGFR2, FGFR3*
5.91E-15	15	103	24.0218963	GO:0060349 Bone morphogenesis	*GLI3, MSX2, TWIST1, RUNX2, ALPL, TGFBR2, AXIN2, CYP26B1, MEGF8, RAB23, LRP5, FREM1, FGFR2, FGFR3, SKI*
1.46E-14	45	1851	4.010138281	GO:0042127 Regulation of cell population proliferation	*FGF9, IL11RA, FGFR2, FGFR1, BMP7, BMP2, RBM38, NOTCH2, TBX3, NOTCH1, SKI, FGFR4, LRP5, TGFBR2, SHH, NELL1, CTNNB1, CASR, ASPM, FGFR3, DLG1, EFNB1, JAG1, GLI3, TGFBR1, SHOC2, MSX2, TWIST1, RUNX2, FGFRL1, KRAS, IGF1R, FTO, GPC3, SPRY1, AXIN2, SHC4, PTCH1, FLNA, CTCF, FOXP1, DIS3L2, KMT2D, ACTB, TSHR*
1.88E-14	16	137	19.26427499	GO:0045667 Regulation of osteoblast differentiation	*NELL1, BMP7, SKI, JAG1, GLI3, MSX2, TWIST1, RUNX2, BMP2, NOTCH1, LRP5, CTNNB1, AXIN2, FAM20C, PTCH1, FGFR2*
2.15E-14	17	167	16.79135346	GO:0048754 Branching morphogenesis of an epithelial tube	*CASR, DLG1, BMP7, GLI3, MSX2, BMP2, KRAS, TBX3, GPC3, NOTCH1, LRP5, TGFBR2, SPRY1, SHH, CTNNB1, PTCH1, FGFR2*
2.36E-14	34	1028	5.455556475	GO:0007167 Enzyme-linked receptor protein signaling pathway	*CASS4, EFNB1, BMP7, FGF9, TGFBR1, BMP2, IGF1R, SKI, TGFBR2, SPRY1, SHC4, EFNA4, LTBP1, FGFR2, FGFR3, FGFR1, FGFR4, BMPER, ERCC2, MEGF8, CBL, MSX2, RUNX2, FGFRL1, NOTCH2, GPC3, NOTCH1, NFIA, FBN1, CTNNB1, FAM20C, ZEB2, SHOC2, PTPRD*
**Syndromic Genes**					
**Enrichment FDR**	**Number of Genes**	**Pathway Genes**	**Fold Enrichment**	**Pathway**	**Genes**
5.45E-25	35	556	12.30321625	GO:0001501 Skeletal system development	*RUNX2, GNAS, FOXP1, FBN1, ALX4, FGFR2, FGFR1, FGF9, MEGF8, GLI3, TGFBR1, MSX2, TWIST1, FGFRL1, CTSK, MCPH1, WDR19, LRP5, NFIA, TGFBR2, SHH, CTNNB1, AXIN2, ASXL1, FAM20C, IFT140, CYP26B1, RAB23, NOTCH2, TBX3, CHD7, SH3PXD2B, FGFR3, SKI, TCOF1*
3.84E-24	23	158	28.45090948	GO:0035107 Appendage morphogenesis	*TBX3, CYP26B1, ALX4, FGFR1, FGF9, MEGF8, GLI3, MSX2, TWIST1, RUNX2, NOTCH2, GPC3, WDR19, LRP5, NFIA, SHH, CTNNB1, CHD7, PTCH1, IFT140, FGFR2, SKI, IFT122*
3.84E-24	23	158	28.45090948	GO:0035108 Limb morphogenesis	*TBX3, CYP26B1, ALX4, FGFR1, FGF9, MEGF8, GLI3, MSX2, TWIST1, RUNX2, NOTCH2, GPC3, WDR19, LRP5, NFIA, SHH, CTNNB1, CHD7, PTCH1, IFT140, FGFR2, SKI, IFT122*
2.82E-23	21	127	32.31773969	GO:0030326 Embryonic limb morphogenesis	*CYP26B1, ALX4, FGFR1, FGF9, MEGF8, GLI3, MSX2, TWIST1, RUNX2, NOTCH2, TBX3, GPC3, WDR19, LRP5, SHH, CTNNB1, CHD7, PTCH1, IFT140, SKI, IFT122*
2.82E-23	21	127	32.31773969	GO:0035113 Embryonic appendage morphogenesis	*CYP26B1, ALX4, FGFR1, FGF9, MEGF8, GLI3, MSX2, TWIST1, RUNX2, NOTCH2, TBX3, GPC3, WDR19, LRP5, SHH, CTNNB1, CHD7, PTCH1, IFT140, SKI, IFT122*
3.00E-23	42	1086	7.558661033	GO:0009887 Animal organ morphogenesis	*FGF9, ASXL3, CHD7, ASXL1, FAM20C, AHDC1, TBX3, CYP26B1, ALX4, ABCC9, FGFR1, JAG1, MEGF8, GLI3, TGFBR1, MSX2, TWIST1, RUNX2, FGFRL1, KRAS, NOTCH2, GPC3, WDR19, LRP5, TGFBR2, SPRY1, SHH, TSHR, CTNNB1, AXIN2, PTCH1, IFT140, FLNA, FBN1, FGFR2, RAB23, BCOR, FGFR3, ZIC1, SKI, IFT122, LHX3*
3.54E-22	23	199	22.58916431	GO:0048736 Appendage development	*TBX3, CYP26B1, ALX4, FGFR1, FGF9, MEGF8, GLI3, MSX2, TWIST1, RUNX2, NOTCH2, GPC3, WDR19, LRP5, NFIA, SHH, CTNNB1, CHD7, PTCH1, IFT140, FGFR2, SKI, IFT122*

3.54E-22	23	199	22.58916431	GO:0060173 Limb development	*TBX3, CYP26B1, ALX4, FGFR1, FGF9, MEGF8, GLI3, MSX2, TWIST1, RUNX2, NOTCH2, GPC3, WDR19, LRP5, NFIA, SHH, CTNNB1, CHD7, PTCH1, IFT140, FGFR2, SKI, IFT122*
1.00E-20	32	623	10.03892793	GO:0007507 Heart development	*TGFBR1, TGFBR2, CHD7, TBX3, ABCC9, FGFR1, JAG1, FGF9, CTCF, MEGF8, GLI3, FOXP1, MSX2, TWIST1, FGFRL1, NOTCH2, GPC3, SKI, SPRY1, SHH, CTNNB1, AXIN2, ASXL1, PTCH1, IFT140, FLNA, FBN1, SH3PXD2B, BCOR, HDAC9, FGFR2, IFT122*
1.32E-19	51	2165	4.604025074	GO:0009888 Tissue development	*RUNX2, FAM20C, GNAS, TGFBR1, AHDC1, TBX3, TGFBR2, SHH, FLNA, CYP26B1, CASR, FGFR2, ABCC9, FGFR1, EFNB1, JAG1, FGF9, CTCF, ERCC2, MEGF8, GLI3, LHX3, FOXP1, MSX2, TWIST1, KRAS, NOTCH2, FTO, ADAMTSL4, CTSK, GPC3, WDR19, SKI, LRP5, NFIA, SPRY1, CTNNB1, AXIN2, CHD7, ASXL1, PTCH1, IFT140, CPT1A, ALX4, TCOF1, ACTB, BCOR, HDAC9, FGFR3, IFT122, SH3PXD2B*
6.84E-19	60	3229	3.631688662	GO:0048513 Animal organ development	*ACTB, FGF9, TGFBR1, RUNX2, ASXL3, TGFBR2, SPRY1, CHD7, ASXL1, FAM20C, GNAS, FOXP1, AHDC1, TBX3, ZIC1, SHH, FBN1, CYP26B1, CASR, ALX4, ASPM, FGFR2, ABCC9, FGFR1, EFNB1, JAG1, CTCF, ERCC2, MEGF8, GLI3, LHX3, CPT1A, CBL, MSX2, TWIST1, FGFRL1, KRAS, NOTCH2, FTO, CTSK, GPC3, MCPH1, WDR19, SKI, LRP5, NFIA, TSHR, CTNNB1, AXIN2, PTCH1, IFT140, FLNA, TCOF1, RAB23, SH3PXD2B, BCOR, HDAC9, FGFR3, IFT122, TCF12*
3.06E-18	56	2868	3.816227746	GO:0009653 Anatomical structure morphogenesis	*ACTB, EFNB1, FGF9, MSX2, NOTCH2, ASXL3, FBN1, CHD7, ASXL1, FAM20C, EFNA4, GNAS, AHDC1, TBX3, FLNA, CYP26B1, CASR, ALX4, FGFR2, ABCC9, FGFR1, JAG1, ERCC2, MEGF8, GLI3, TGFBR1, LHX3, CNOT2, TWIST1, RUNX2, FGFRL1, KRAS, GPC3, PTPRD, WDR19, LRP5, NFIA, TGFBR2, SPRY1, SHH, TSHR, CTNNB1, AXIN2, PTCH1, IFT140, HDAC9, TCOF1, HUWE1, RAB23, FOXP1, DIAPH1, BCOR, FGFR3, ZIC1, SKI, IFT122*
3.48E-18	21	228	18.00154799	GO:0060348 Bone development	*GNAS, FOXP1, FBN1, FGFR2, GLI3, MSX2, TWIST1, RUNX2, MCPH1, LRP5, TGFBR2, AXIN2, ASXL1, FAM20C, CYP26B1, MEGF8, RAB23, NOTCH2, SH3PXD2B, FGFR3, SKI*
6.94E-17	26	491	10.34944976	GO:0007389 Pattern specification process	*CYP26B1, ALX4, FGFR1, EFNB1, MEGF8, GLI3, TGFBR1, LHX3, MSX2, NOTCH2, TBX3, GPC3, ZIC1, WDR19, LRP5, TGFBR2, SPRY1, SHH, CTNNB1, AXIN2, PTCH1, IFT140, BCOR, FGFR2, SKI, IFT122*
3.53E-16	36	1197	5.878056486	GO:0009790 Embryo development	*MSX2, CHD7, AHDC1, SHH, CYP26B1, ALX4, FGFR2, FGFR1, EFNB1, JAG1, FGF9, CTCF, ERCC2, MEGF8, GLI3, TGFBR1, CNOT2, TWIST1, RUNX2, NOTCH2, TBX3, GPC3, WDR19, LRP5, TGFBR2, TSHR, CTNNB1, AXIN2, BCOR, PTCH1, IFT140, FBN1, TCOF1, ZIC1, SKI, IFT122*
4.47E-16	28	648	8.445170661	GO:0048598 Embryonic morphogenesis	*MSX2, CHD7, AHDC1, CYP26B1, ALX4, FGFR2, FGFR1, FGF9, MEGF8, GLI3, TGFBR1, TWIST1, RUNX2, NOTCH2, TBX3, GPC3, WDR19, LRP5, TGFBR2, SHH, TSHR, CTNNB1, PTCH1, IFT140, FBN1, ZIC1, SKI, IFT122*
7.08E-16	40	1566	4.99221911	GO:0009719 Response to endogenous stimulus	*FGF9, TGFBR1, POR, SKI, TGFBR2, SPRY1, TSHR, HDAC9, LTBP1, FGFR2, FGFR3, SMC1A, FGFR1, GNAS, FOXP1, FGFR4, KMT2D, CYP26B1, CASR, ABCC9, MEGF8, SHOC2, CBL, MSX2, TWIST1, RUNX2, FGFRL1, NOTCH2, CTSK, GPC3, LRP5, NFIA, FBN1, CTNNB1, AXIN2, ASXL1, FAM20C, CNOT2, TCF12, ZEB2*
2.08E-15	67	4782	2.738360589	GO:0030154 Cell differentiation	*ACTB, FGFR1, OSTM1, EFNB1, FGF9, LHX3, RUNX2, NOTCH2, TBX3, SHH, AXIN2, DDX3X, EFNA4, CETP, KAT6A, CTCF, TGFBR1, FOXP1, SKI, TGFBR2, FBN1, CYP26B1, HDAC9, ASPM, FGFR2, HUWE1, JAG1, ERCC2, DMPK, MEGF8, PPP2R1A, GLI3, SHOC2, CNOT2, MSX2, TWIST1, IFT81, AHDC1, KRAS, TCF12, ADAMTSL4, CTSK, GPC3, ZIC1, PTPRD, SPAG17, LRP5, NFIA, SPRY1, TSHR, CTNNB1, ZEB2, CHD7, ESCO2, ASXL1, SH3PXD2B, FAM20C, SHC4, PTCH1, IFT140, FLNA, CPT1A, TCOF1, FTO, KMT2D, KAT6B, FGFR3*
2.08E-15	67	4783	2.737788069	GO:0048869 Cellular developmental process	*ACTB, FGFR1, OSTM1, EFNB1, FGF9, LHX3, RUNX2, NOTCH2, TBX3, SHH, AXIN2, DDX3X, EFNA4, CETP, KAT6A, CTCF, TGFBR1, FOXP1, SKI, TGFBR2, FBN1, CYP26B1, HDAC9, ASPM, FGFR2, HUWE1, JAG1, ERCC2, DMPK, MEGF8, PPP2R1A, GLI3, SHOC2, CNOT2, MSX2, TWIST1, IFT81, AHDC1, KRAS, TCF12, ADAMTSL4, CTSK, GPC3, ZIC1, PTPRD, SPAG17, LRP5, NFIA, SPRY1, TSHR, CTNNB1, ZEB2, CHD7, ESCO2, ASXL1, SH3PXD2B, FAM20C, SHC4, PTCH1, IFT140, FLNA, CPT1A, TCOF1, FTO, KMT2D, KAT6B, FGFR3*
2.97E-15	19	240	15.4727591	GO:0048705 Skeletal system morphogenesis	*ALX4, MEGF8, GLI3, TGFBR1, MSX2, TWIST1, RUNX2, WDR19, TGFBR2, CTNNB1, AXIN2, IFT140, CYP26B1, FGFR2, RAB23, LRP5, FGFR3, SKI, FGFR1*
2.97E-15	36	1293	5.441634659	GO:0071495 Cellular response to endogenous stimulus	*FGF9, TGFBR1, SKI, TGFBR2, SPRY1, TSHR, HDAC9, LTBP1, FGFR2, FGFR3, SMC1A, FGFR1, FOXP1, FGFR4, CYP26B1, CASR, MEGF8, SHOC2, CBL, MSX2, TWIST1, RUNX2, FGFRL1,*

					NOTCH2, CTSK, GPC3, NFIA, FBN1, CTNNB1, AXIN2, ASXL1, FAM20C, CNOT2, KMT2D, ZEB2, GNAS
4.72E-15	36	1314	5.354667895	GO:0060429 Epithelium development	*GNAS, SHH, FLNA, CYP26B1, CASR, FGFR1, JAG1, ERCC2, MEGF8, GLI3, LHX3, MSX2, TWIST1, KRAS, NOTCH2, TBX3, ADAMTSL4, GPC3, WDR19, LRP5, TGFBR2, SPRY1, CTNNB1, AXIN2, CHD7, ASXL1, FAM20C, PTCH1, IFT140, CPT1A, ALX4, ACTB, FOXP1, FGFR2, SKI, IFT122*
9.60E-15	40	1703	4.590613697	GO:0045595 Regulation of cell differentiation	*FGFR1, RUNX2, CETP, FOXP1, NOTCH2, TBX3, SKI, TGFBR2, FBN1, CYP26B1, ASPM, JAG1, FGF9, DMPK, MEGF8, GLI3, TGFBR1, SHOC2, MSX2, TWIST1, KRAS, TCF12, PTPRD, LRP5, SPRY1, SHH, CTNNB1, AXIN2, CHD7, ASXL1, FAM20C, PTCH1, FTO, HDAC9, FGFR2, ZEB2, ACTB, KAT6A, PPP2R1A, KAT6B*
1.12E-14	23	447	10.0564736	GO:0003002 Regionalization	*CYP26B1, ALX4, FGFR1, MEGF8, GLI3, TGFBR1, LHX3, MSX2, NOTCH2, TBX3, GPC3, WDR19, LRP5, TGFBR2, SPRY1, SHH, CTNNB1, AXIN2, PTCH1, IFT140, FGFR2, SKI, IFT122*
1.14E-14	34	1192	5.574784276	GO:0072359 Circulatory system development	*TGFBR1, TGFBR2, CHD7, TBX3, ABCC9, FGFR1, JAG1, FGF9, CTCF, MEGF8, GLI3, FOXP1, MSX2, TWIST1, FGFRL1, NOTCH2, GPC3, SKI, LRP5, SPRY1, SHH, CTNNB1, AXIN2, ANTXR1, ASXL1, PTCH1, IFT140, FLNA, HDAC9, FBN1, SH3PXD2B, BCOR, FGFR2, IFT122*
1.14E-14	13	75	33.87719888	GO:1904888 Cranial skeletal system development	*GLI3, TGFBR1, MSX2, TWIST1, RUNX2, WDR19, TGFBR2, CTNNB1, IFT140, FGFR2, MEGF8, RAB23, COLEC10*
1.49E-14	49	2666	3.592206875	GO:0050793 Regulation of developmental process	*FGFR1, CNOT2, RUNX2, CETP, FOXP1, NOTCH2, TBX3, SKI, TGFBR2, SHH, FBN1, CYP26B1, ASPM, JAG1, FGF9, DMPK, MEGF8, GLI3, TGFBR1, SHOC2, MSX2, TWIST1, KRAS, TCF12, FTO, CTSK, PTPRD, LRP5, SPRY1, TSHR, CTNNB1, AXIN2, CHD7, ASXL1, FAM20C, PTCH1, FLNA, HUWE1, DIAPH1, BCOR, HDAC9, FGFR2, FGFR3, GNAS, ZEB2, ACTB, KAT6A, PPP2R1A, KAT6B*
2.45E-14	20	317	12.33093868	GO:0048562 Embryonic organ morphogenesis	*CHD7, ALX4, FGFR1, FGF9, MEGF8, GLI3, TGFBR1, TWIST1, RUNX2, NOTCH2, TBX3, WDR19, TGFBR2, SHH, TSHR, CTNNB1, IFT140, FBN1, FGFR2, ZIC1*
2.58E-14	19	275	13.50349885	GO:0003007 Heart morphogenesis	*CHD7, ABCC9, JAG1, MEGF8, TGFBR1, MSX2, TWIST1, FGFRL1, NOTCH2, TBX3, TGFBR2, SPRY1, SHH, CTNNB1, AXIN2, ASXL1, PTCH1, FLNA, FGFR2*
2.58E-14	26	645	7.878418344	GO:0007423 Sensory organ development	*CHD7, CYP26B1, FGFR1, JAG1, FGF9, GLI3, TGFBR1, NOTCH2, TBX3, WDR19, LRP5, NFIA, TGFBR2, SPRY1, SHH, TSHR, CTNNB1, IFT140, LHX3, FBN1, TWIST1, SH3PXD2B, FGFR2, ZIC1, SKI, IFT122*
2.90E-14	33	1155	5.584153661	GO:0035295 Tube development	*CASR, ALX4, FGFR1, JAG1, FGF9, MEGF8, GLI3, TGFBR1, LHX3, MSX2, TWIST1, KRAS, NOTCH2, TBX3, GPC3, WDR19, LRP5, NFIA, TGFBR2, SPRY1, SHH, FBN1, CTNNB1, CHD7, ASXL1, PTCH1, IFT140, FLNA, HDAC9, FOXP1, FGFR2, SKI, IFT122*
2.93E-14	26	651	7.805806193	GO:0048729 Tissue morphogenesis	*AHDC1, FLNA, CASR, FGFR2, FGFR1, JAG1, MEGF8, GLI3, TGFBR1, MSX2, TWIST1, KRAS, NOTCH2, TBX3, GPC3, LRP5, TGFBR2, SPRY1, SHH, CTNNB1, CHD7, PTCH1, ACTB, FOXP1, SKI, IFT122*
4.11E-14	52	3099	3.279496503	GO:0007166 Cell surface receptor signaling pathway	*EFNB1, FGF9, GLI3, TGFBR1, TCTN3, TRAF7, NOTCH2, IL11RA, GPC3, SKI, LRP5, TGFBR2, SPRY1, SHH, CTNNB1, AXIN2, SHC4, PTCH1, EFNA4, LTBP1, FGFR2, FGFR3, FGFR1, FGFR4, ASPM, JAG1, ERCC2, DMPK, MEGF8, PPP2R1A, SHOC2, CBL, MSX2, RUNX2, FGFRL1, ZIC1, PTPRD, WDR19, NFIA, FBN1, FAM20C, IFT140, FLNA, FOXP1, IFT81, TSHR, ZEB2, DDX3X, IFT122, COLEC11, COLEC10, P4HB*
5.05E-14	23	487	9.230479872	GO:0048568 Embryonic organ development	*CHD7, ALX4, FGFR1, FGF9, ERCC2, MEGF8, GLI3, TGFBR1, TWIST1, RUNX2, NOTCH2, TBX3, WDR19, TGFBR2, SHH, TSHR, CTNNB1, PTCH1, IFT140, FBN1, FGFR2, ZIC1, IFT122*
5.59E-14	61	4287	2.781004914	GO:0048731 System development	*ACTB, FGF9, EFNB1, TGFBR1, LHX3, RUNX2, NOTCH2, ZIC1, LRP5, TGFBR2, SHH, CHD7, EFNA4, GNAS, FOXP1, TBX3, PTPRD, FBN1, CYP26B1, HDAC9, ALX4, ASPM, FGFR2, ABCC9, FGFR1, JAG1, CTCF, ERCC2, MEGF8, GLI3, SHOC2, CPT1A, CBL, MSX2, TWIST1, FGFRL1, KRAS, TCF12, CTSK, GPC3, MCPH1, WDR19, SKI, NFIA, SPRY1, TSHR, CTNNB1, AXIN2, ZEB2, ANTXR1, ASXL1, FAM20C, PTCH1, IFT140, FLNA, RAB23, SH3PXD2B, BCOR, FGFR3, IFT122, TCOF1*
8.76E-14	26	685	7.418364718	GO:0043009 Chordate embryonic development	*CHD7, ALX4, FGFR1, FGF9, CTCF, ERCC2, MEGF8, GLI3, TGFBR1, CNOT2, TWIST1, RUNX2, NOTCH2, TBX3, WDR19, TGFBR2, SHH, CTNNB1, AXIN2, BCOR, PTCH1, IFT140, FGFR2, TCOF1, SKI, IFT122*
1.02E-13	51	3054	3.263822621	GO:0048468 Cell development	*ACTB, OSTM1, EFNB1, RUNX2, NOTCH2, AXIN2, EFNA4, KAT6A, CTCF, FOXP1, TBX3, SHH, FBN1, CYP26B1, ASPM, FGFR1, JAG1, ERCC2, MEGF8, PPP2R1A, GLI3, LHX3, SHOC2, MSX2, TWIST1, IFT81, KRAS, ADAMTSL4, CTSK, GPC3, PTPRD, SPAG17, LRP5, TGFBR2, SPRY1, TSHR, CTNNB1,*

					ZEB2, CHD7, ESCO2, ASXL1, FAM20C, PTCH1, IFT140, FLNA, SKI, TCOF1, HDAC9, FGFR2, KMT2D, KAT6B
1.62E-13	43	2185	3.846293483	GO:0008283 Cell population proliferation	*FGF9, IL11RA, FGFR2, FGFR1, NOTCH2, TBX3, SKI, FGFR4, LRP5, TGFBR2, SHH, CTNNB1, CASR, ASPM, FGFR3, ABCC9, EFNB1, JAG1, ERCC2, GLI3, TGFBR1, SHOC2, DPH1, MSX2, TWIST1, RUNX2, FGFRL1, KRAS, FTO, GPC3, NFIA, SPRY1, AXIN2, ZEB2, SHC4, PTCH1, FLNA, CTCF, FOXP1, DIS3L2, KMT2D, ACTB, TSHR*
1.66E-13	26	706	7.197705144	GO:0009792 Embryo development ending in birth or egg hatching	*CHD7, ALX4, FGFR1, FGF9, CTCF, ERCC2, MEGF8, GLI3, TGFBR1, CNOT2, TWIST1, RUNX2, NOTCH2, TBX3, WDR19, TGFBR2, SHH, CTNNB1, AXIN2, BCOR, PTCH1, IFT140, FGFR2, TCOF1, SKI, IFT122*
2.07E-13	15	154	19.03688748	GO:0007224 Smoothened signaling pathway	*GLI3, TCTN3, SHH, PTCH1, FGF9, MEGF8, RUNX2, GPC3, ZIC1, WDR19, TGFBR2, IFT140, IFT81, FGFR2, IFT122*
2.13E-13	26	715	7.10710466	GO:0071363 Cellular response to growth factor stimulus	*FGF9, TGFBR1, SKI, TGFBR2, SPRY1, LTBP1, FGFR2, FGFR3, FGFR1, FGFR4, CASR, MEGF8, SHOC2, CBL, MSX2, TWIST1, RUNX2, FGFRL1, NOTCH2, CTSK, GPC3, NFIA, FBN1, CTNNB1, FAM20C, ZEB2*
2.84E-13	23	533	8.433853091	GO:0002009 Morphogenesis of an epithelium	*FLNA, CASR, FGFR1, JAG1, MEGF8, GLI3, MSX2, TWIST1, KRAS, NOTCH2, TBX3, GPC3, LRP5, TGFBR2, SPRY1, SHH, CTNNB1, PTCH1, ACTB, FOXP1, FGFR2, SKI, IFT122*
5.00E-13	13	103	24.66786326	GO:0060349 Bone morphogenesis	*GLI3, MSX2, TWIST1, RUNX2, TGFBR2, AXIN2, CYP26B1, MEGF8, RAB23, LRP5, FGFR2, FGFR3, SKI*
5.56E-13	26	747	6.802650377	GO:0070848 Response to growth factor	*FGF9, TGFBR1, SKI, TGFBR2, SPRY1, LTBP1, FGFR2, FGFR3, FGFR1, FGFR4, CASR, MEGF8, SHOC2, CBL, MSX2, TWIST1, RUNX2, FGFRL1, NOTCH2, CTSK, GPC3, NFIA, FBN1, CTNNB1, FAM20C, ZEB2*
1.31E-12	18	300	11.72672269	GO:0090596 Sensory organ morphogenesis	*CHD7, CYP26B1, FGFR1, JAG1, FGF9, GLI3, NOTCH2, TBX3, WDR19, TSHR, CTNNB1, FBN1, TWIST1, LRP5, FGFR2, ZIC1, SKI, IFT122*
1.88E-12	19	356	10.43107355	GO:0060562 Epithelial tube morphogenesis	*CASR, MEGF8, GLI3, MSX2, TWIST1, KRAS, NOTCH2, TBX3, GPC3, LRP5, TGFBR2, SPRY1, SHH, CTNNB1, PTCH1, FOXP1, FGFR2, SKI, IFT122*
2.73E-12	38	1851	4.012384857	GO:0042127 Regulation of cell population proliferation	*FGF9, IL11RA, FGFR2, FGFR1, NOTCH2, TBX3, SKI, FGFR4, LRP5, TGFBR2, SHH, CTNNB1, CASR, ASPM, FGFR3, EFNB1, JAG1, GLI3, TGFBR1, SHOC2, MSX2, TWIST1, RUNX2, FGFRL1, KRAS, FTO, GPC3, SPRY1, AXIN2, SHC4, PTCH1, FLNA, CTCF, FOXP1, DIS3L2, KMT2D, ACTB, TSHR*
3.00E-12	12	91	25.7730169	GO:0008589 Regulation of smoothened signaling pathway	*PTCH1, SHH, FGF9, MEGF8, GLI3, RUNX2, GPC3, ZIC1, IFT140, IFT81, FGFR2, IFT122*
7.05E-12	16	239	13.08420942	GO:0060541 Respiratory system development	*FGFR1, FGF9, GLI3, LHX3, FGFRL1, KRAS, GPC3, TGFBR2, SPRY1, SHH, FBN1, CTNNB1, CHD7, ASXL1, FGFR2, SKI*

Nonsyndromic Genes

Enrichment FDR	Number of Genes	Pathway Genes	Fold Enrichment	Pathway	Genes
7.61E-07	10	556	14.42446043	GO:0001501 Skeletal system development	*RUNX2, BMP2, ALX4, DLG1, BMP7, ALPL, TGFBR2, RAB23, FREM1, SH3PXD2B*
7.61E-07	8	240	26.73333333	GO:0048705 Skeletal system morphogenesis	*ALX4, DLG1, BMP7, RUNX2, ALPL, TGFBR2, RAB23, FREM1*
3.91E-06	5	53	75.66037736	GO:0003197 Endocardial cushion development	*BMP7, JAG1, BMP2, NOTCH1, TGFBR2*
4.19E-06	5	59	67.96610169	GO:0010718 Positive regulation of epithelial to mesenchymal transition	*BMP2, TGFBR2, BMP7, NOTCH1, JAG1*
4.19E-06	7	228	24.62280702	GO:0060348 Bone development	*BMP2, RUNX2, ALPL, TGFBR2, RAB23, FREM1, SH3PXD2B*
5.20E-06	7	247	22.72874494	GO:0001649 Osteoblast differentiation	*ALPL, NELL1, BMP7, BMP2, JAG1, RUNX2, NOTCH1*

5.20E-06	6	137	35.12408759	GO:0045667 Regulation of osteoblast differentiation	*NELL1, BMP7, JAG1, RUNX2, BMP2, NOTCH1*
6.25E-06	5	72	55.69444444	GO:0045669 Positive regulation of osteoblast differentiation	*BMP7, NELL1, JAG1, RUNX2, BMP2*
6.25E-06	17	3229	4.222359864	GO:0048513 Animal organ development	*PCK1, RUNX2, TGFBR2, BMP7, BMP2, NELL1, ALX4, SPO11, DLG1, JAG1, IGF1R, NOTCH1, ALPL, BMPER, RAB23, FREM1, SH3PXD2B*
6.44E-06	11	1086	8.123388582	GO:0009887 Animal organ morphogenesis	*BMP2, ALX4, DLG1, BMP7, JAG1, RUNX2, NOTCH1, ALPL, TGFBR2, RAB23, FREM1*
6.44E-06	6	156	30.84615385	GO:0072073 Kidney epithelium development	*DLG1, BMP7, JAG1, BMP2, NOTCH1, BMPER*
6.62E-06	4	28	114.5714286	GO:0003272 Endocardial cushion formation	*BMP7, BMP2, NOTCH1, TGFBR2*
9.67E-06	6	172	27.97674419	GO:0003205 Cardiac chamber development	*BMP7, JAG1, BMP2, IGF1R, NOTCH1, TGFBR2*
1.10E-05	4	33	97.21212121	GO:0060317 Cardiac epithelial to mesenchymal transition	*BMP2, NOTCH1, TGFBR2, JAG1*
1.10E-05	5	89	45.05617978	GO:0060395 Smad protein signal transduction	*BMP7, BMP2, TGFBR2, BMPER, RUNX2*
1.43E-05	5	95	42.21052632	GO:0048704 Embryonic skeletal system morphogenesis	*ALX4, DLG1, BMP7, RUNX2, TGFBR2*
1.65E-05	6	197	24.42639594	GO:0031214 Biomineral tissue development	*BMP7, BMP2, NELL1, RUNX2, NOTCH1, ALPL*
1.65E-05	3	9	267.3333333	GO:0062043 Positive regulation of cardiac epithelial to mesenchymal transition	*NOTCH1, TGFBR2, JAG1*
1.80E-05	5	103	38.93203883	GO:0060349 Bone morphogenesis	*RUNX2, ALPL, TGFBR2, RAB23, FREM1*
1.94E-05	4	41	78.24390244	GO:0003203 Endocardial cushion morphogenesis	*BMP7, BMP2, NOTCH1, TGFBR2*
2.01E-05	3	10	240.6	GO:0062042 Regulation of cardiac epithelial to mesenchymal transition	*NOTCH1, TGFBR2, JAG1*
2.16E-05	5	110	36.45454545	GO:0090100 Positive regulation of transmembrane receptor protein serine/threonine kinase signaling pathway	*BMP7, BMP2, TGFBR2, BMPER, NOTCH1*
2.37E-05	4	45	71.28888889	GO:0003176 Aortic valve development	*BMP2, NOTCH1, TGFBR2, JAG1*
2.37E-05	5	114	35.1754386	GO:0010717 Regulation of epithelial to mesenchymal transition	*BMP2, TGFBR2, BMP7, NOTCH1, JAG1*
2.49E-05	4	46	69.73913043	GO:0060391 Positive regulation of smad protein signal transduction	*BMP7, BMP2, TGFBR2, BMPER*
3.34E-05	5	125	32.08	GO:0003206 Cardiac chamber morphogenesis	*BMP7, JAG1, BMP2, NOTCH1, TGFBR2*
3.34E-05	4	50	64.16	GO:1905314 Semi-lunar valve development	*BMP2, NOTCH1, JAG1, TGFBR2*

4.22E-05	5	132	30.37878788	GO:0048706 Embryonic skeletal system development	*ALX4, DLG1, BMP7, RUNX2, TGFBR2*
4.26E-05	13	2165	4.815704388	GO:0009888 Tissue development	*PCK1, RUNX2, BMP7, BMP2, TGFBR2, NELL1, DLG1, JAG1, NOTCH1, ALPL, BMPER, ALX4, SH3PXD2B*
4.26E-05	5	135	29.7037037	GO:0030278 Regulation of ossification	*NELL1, BMP7, BMP2, RUNX2, NOTCH1*
4.26E-05	6	256	18.796875	GO:0048863 Stem cell differentiation	*BMP7, JAG1, RUNX2, NOTCH1, SHC4, TGFBR2*
4.94E-05	5	140	28.64285714	GO:0030282 Bone mineralization	*BMP7, BMP2, NELL1, RUNX2, ALPL*
4.99E-05	8	645	9.947286822	GO:0007423 Sensory organ development	*DLG1, BMP7, JAG1, BMP2, NOTCH1, TGFBR2, BMPER, SH3PXD2B*
4.99E-05	9	890	8.11011236	GO:0022603 Regulation of anatomical structure morphogenesis	*BMPER, BMP2, BMP7, JAG1, RUNX2, NOTCH1, TGFBR2, ADCK1, DLG1*
4.99E-05	4	59	54.37288136	GO:0072132 Mesenchyme morphogenesis	*BMP7, BMP2, NOTCH1, TGFBR2*
5.45E-05	3	16	150.375	GO:0061101 Neuroendocrine cell differentiation	*JAG1, BMP2, NOTCH1*
6.10E-05	7	454	12.36563877	GO:0001503 Ossification	*ALPL, RUNX2, NELL1, BMP7, BMP2, JAG1, NOTCH1*
6.29E-05	4	64	50.125	GO:0003179 Heart valve morphogenesis	*BMP2, NOTCH1, TGFBR2, JAG1*
6.96E-05	18	4782	3.018820577	GO:0030154 Cell differentiation	*ALPL, PCK1, RUNX2, NOTCH1, NELL1, BMP7, BMP2, TGFBR2, SPO11, JAG1, BBS9, CTCFL, FAM209A, RBM38, IGF1R, PDILT, SH3PXD2B, SHC4*
6.96E-05	18	4783	3.018189421	GO:0048869 Cellular developmental process	*ALPL, PCK1, RUNX2, NOTCH1, NELL1, BMP7, BMP2, TGFBR2, SPO11, JAG1, BBS9, CTCFL, FAM209A, RBM38, IGF1R, PDILT, SH3PXD2B, SHC4*
6.99E-05	12	1931	4.983946142	GO:0051240 Positive regulation of multicellular organismal process	*NELL1, BMP7, BMP2, TGFBR2, PCK1, RUNX2, PTH2R, NOTCH1, ALPL, BMPER, JAG1, IGF1R*
7.34E-05	5	163	24.60122699	GO:0030509 Bmp signaling pathway	*BMP7, BMP2, RUNX2, NOTCH1, BMPER*
7.34E-05	17	4287	3.180312573	GO:0048731 System development	*PCK1, RUNX2, NOTCH1, TGFBR2, BMPER, BMP7, BMP2, ALX4, SPO11, DLG1, JAG1, IGF1R, ALPL, RAB23, FREM1, SH3PXD2B, NELL1*
7.34E-05	3	19	126.6315789	GO:0060039 Pericardium development	*BMP7, BMP2, NOTCH1*
7.34E-05	4	70	45.82857143	GO:0060411 Cardiac septum morphogenesis	*BMP7, JAG1, NOTCH1, TGFBR2*
7.34E-05	6	302	15.93377483	GO:0061448 Connective tissue development	*RUNX2, BMP7, BMP2, NOTCH1, TGFBR2, SH3PXD2B*
7.34E-05	5	163	24.60122699	GO:0072006 Nephron development	*BMP7, DLG1, JAG1, BMP2, NOTCH1*
7.84E-05	5	167	24.01197605	GO:0048754 Branching morphogenesis of an epithelial tube	*DLG1, BMP7, BMP2, NOTCH1, TGFBR2*
7.84E-05	4	72	44.55555556	GO:0060390 Regulation of smad protein signal transduction	*BMP7, BMP2, TGFBR2, BMPER*

Table S6. GO Cellular Component.

All Genes					
Enrichment FDR	Number of Genes	Pathway Genes	Fold Enrichment	Pathway	Genes
3.80E-05	4	8	82.4751773	GO:0030991 Intraciliary transport particle a	*WDR35, WDR19, IFT122, IFT140*
4.74E-05	5	25	32.99007092	GO:0030990 Intraciliary transport particle	*WDR35, IFT81, WDR19, IFT122, IFT140*
4.74E-05	14	433	5.333267816	GO:0043235 Receptor complex	*FGFR2, FGFR3, FGFR1, TGFBR1, NOTCH2, IL11RA, IGF1R, NOTCH1, FGFR4, TGFBR2, BMP2, LRP5, TSHR, FLNA*
4.74E-05	6	48	20.61879433	GO:0097542 Ciliary tip	*GLI3, WDR35, IFT81, WDR19, IFT122, IFT140*
0.001044688	50	4670	1.766063754	GO:0005654 Nucleoplasm	*CDC45, GINS2, KMT2D, ALX4, TCOF1, SMC1A, KAT6A, HUWE1, CTCF, ERCC2, ERF, GLI3, SHOC2, TRIM37, EFTUD2, DPH1, CNOT2, MSX2, CTCFL, RUNX2, MASP1, NOTCH2, TCF12, FTO, CTSK, ZNF462, ZIC1, SKI, RECQL4, NFIA, SPRY1, HNRNPK, ZEB2, CHD7, ESCO2, FAM20C, BCOR, PPP1CB, DDX3X, MCPH1, HDAC9, ACTB, FOXP1, TWIST1, MED13L, TBX3, NOTCH1, KAT6B, CTNNB1, ASXL1*
0.001044688	30	2160	2.290977147	GO:0005694 Chromosome	*ACTB, SPO11, SMC1A, KAT6A, CTCFL, GINS2, TBX3, TCF12, KAT6B, RECQL4, CHD7, ESCO2, RTF2, CTCF, PPP2R1A, HNRNPK, CTNNB1, ZEB2, PPP1CB, CDC45, LHX3, TRIM37, RUNX2, AHDC1, ALX4, ERF, FOXP1, MSX2, TWIST1, NFIA*
0.00545057	18	1085	2.736503579	GO:0009986 Cell surface	*ANTXR1, NOTCH2, IL11RA, GPC3, NOTCH1, P4HB, FGFR2, EFNB1, TGFBR1, BMP2, TGFBR2, CASR, FGFR3, CTSK, SHH, TSHR, COLEC11, COLEC10*
0.00545057	2	3	109.9669031	GO:0070021 Transforming growth factor beta ligand-receptor complex	*TGFBR1, TGFBR2*
0.00545057	17	996	2.815417699	GO:0070161 Anchoring junction	*ACTB, KRAS, HNRNPK, CTNNB1, P4HB, FLNA, PPP1CB, DLG1, CASS4, TGFBR1, FGFRL1, FGFR4, JAG1, CBL, NOTCH1, SH3PXD2B, FGFR3*
0.005694613	15	816	3.032175636	GO:0005929 Cilium	*WDR35, BBS9, IFT81, SPAG17, WDR19, IFT122, IFT140, CDC45, GLI3, CBL, CIMIP1, NOTCH2, TBX3, PTCH1, TCTN3*
0.006375909	9	331	4.485054959	GO:0005788 Endoplasmic reticulum lumen	*P4HB, LTBP1, MBTPS1, ADAMTSL4, GPC3, SHH, FBN1, CRTAP, FAM20C*
0.006375909	9	329	4.512319731	GO:0045121 Membrane raft	*CBL, TGFBR1, TGFBR2, PTCH1, DLG1, EFNB1, IGF1R, SHH, CTNNB1*
0.006375909	9	331	4.485054959	GO:0098857 Membrane microdomain	*CBL, TGFBR1, TGFBR2, PTCH1, DLG1, EFNB1, IGF1R, SHH, CTNNB1*
0.006625293	2	4	82.4751773	GO:0035517 Pr-dub complex	*ASXL3, ASXL1*
0.006992608	5	92	8.964693185	GO:0005901 Caveola	*TGFBR2, PTCH1, CBL, IGF1R, CTNNB1*
0.008259644	46	4675	1.623040922	GO:0005576 Extracellular region	*LTBP1, ACTB, DLG1, HUWE1, CETP, GNAS, EFNB1, MEGF8, PPP2R1A, FAM209A, PCK1, B3GAT3, PTPRD, ALPL, HNRNPK, FBN1, CTNNB1, FAM20C, P4HB, FLNA, PPP1CB, DDX3X, BMP7, FGF9, COLEC11, BMP2, MASP1, CTSK, BMPER, SHH, NELL1, COLEC10, CRTAP, FGFR2, FGFR3, IL11RA, ADAMTSL4, SPAG17, FGFR4, TGFBR2, FREM1, EFNA4, FGFR1, JAG1, NOTCH2, NOTCH1*
0.008956992	20	1417	2.328163086	GO:0098590 Plasma membrane region	*DLG1, EFNB1, BBS9, TGFBR2, TSHR, CTNNB1, ANTXR1, PTCH1, CASR, ASPM, GNAS, JAG1, CBL, DIAPH1, IGF1R, CTSK, NOTCH1, PTPRD, SHC4, TCTN3*
0.008979681	24	1884	2.101278403	GO:0005794 Golgi apparatus	*MBTPS1, HUWE1, GNPTAB, CHST3, B3GAT3, FGFR3, DLG1, GNAS, CBL, FGFRL1, FGFR4, SPRY1, SHH, ESCO2, FAM20C, PTCH1, FGFR2, NOTCH2, GPC3, NOTCH1, SPAG17, COLEC10, FLNA, KRAS*
0.008979681	14	802	2.879432624	GO:0005813 Centrosome	*IFT81, CEP135, CDC45, RAB23, BBS9, SKI, CTNNB1, AXIN2, IFT140, DDX3X, ASPM, WDR35, DIAPH1, MCPH1*

0.014260717	15	953	2.596280503	GO:0005815 Microtubule organizing center	*IFT81, CEP135, IFT140, CDC45, RAB23, BBS9, SKI, IFT122, CTNNB1, AXIN2, DDX3X, ASPM, WDR35, DIAPH1, MCPH1*
0.014260717	29	2572	1.859860141	GO:0042995 Cell projection	*ACTB, DLG1, WDR35, BBS9, IFT81, IGF1R, SPAG17, WDR19, IFT122, IFT140, CDC45, PPP2R1A, GLI3, CBL, CIMIP1, ANTXR1, P4HB, CASR, DIAPH1, NOTCH2, TBX3, CYP17A1, HNRNPK, CTNNB1, SH3PXD2B, PTCH1, FLNA, DDX3X, TCTN3*
0.014637801	10	490	3.366333768	GO:0005925 Focal adhesion	*ACTB, KRAS, HNRNPK, CTNNB1, P4HB, FLNA, PPP1CB, CASS4, CBL, FGFR3*
0.014637801	20	1532	2.153398885	GO:0015630 Microtubule cytoskeleton	*IFT81, CEP135, IFT140, ASPM, SMC1A, DLG1, CDC45, ERCC2, RAB23, BBS9, DIAPH1, SKI, IFT122, CTNNB1, AXIN2, DDX3X, WDR35, MCPH1, SPAG17, PPP2R1A*
0.014637801	29	2605	1.836299533	GO:0030054 Cell junction	*ACTB, KRAS, HNRNPK, CTNNB1, P4HB, FLNA, PPP1CB, DLG1, EFNB1, CASS4, TGFBR1, DPH1, RAB23, FGFRL1, PTPRD, FGFR4, NFIA, ESCO2, CASR, FGFR1, JAG1, PPP2R1A, CBL, NOTCH1, SH3PXD2B, SHC4, PTCH1, FGFR2, FGFR3*
0.014637801	10	500	3.299007092	GO:0030055 Cell-substrate junction	*ACTB, KRAS, HNRNPK, CTNNB1, P4HB, FLNA, PPP1CB, CASS4, CBL, FGFR3*
0.014637801	11	595	3.049502354	GO:0030312 External encapsulating structure	*LTBP1, ADAMTSL4, SHH, FBN1, BMPER, FREM1, FGFR2, DLG1, BMP7, GPC3, ALPL*
0.014637801	11	594	3.054636196	GO:0031012 Extracellular matrix	*LTBP1, ADAMTSL4, SHH, FBN1, BMPER, FREM1, FGFR2, DLG1, BMP7, GPC3, ALPL*
0.014637801	4	73	9.038375595	GO:0035869 Ciliary transition zone	*BBS9, WDR19, IFT140, IFT122*
0.014637801	2	8	41.23758865	GO:0070776 Moz/morf histone acetyltransferase complex	*KAT6A, KAT6B*
0.014637801	24	1990	1.98935101	GO:1902494 Catalytic complex	*ACTB, KAT6A, GNAS, PPP2R1A, TGFBR1, EFTUD2, IGF1R, KAT6B, KMT2D, AXIN2, ERCC2, SHOC2, TRIM37, TRAF7, HNRNPK, CTNNB1, BCOR, P4HB, PPP1CB, TGFBR2, HDAC9, DPH1, COLEC11, COLEC10*
0.015838821	5	129	6.393424597	GO:0044853 Plasma membrane raft	*TGFBR2, PTCH1, CBL, IGF1R, CTNNB1*
0.017048291	2	9	36.65563436	GO:0070775 H3 histone acetyltransferase complex	*KAT6A, KAT6B*
0.018711544	29	2693	1.776294201	GO:0005856 Cytoskeleton	*ACTB, WDR35, IFT81, DIAPH1, SPAG17, CEP135, IFT140, FLNA, ASPM, SMC1A, DLG1, CDC45, ERCC2, RAB23, BBS9, SKI, IFT122, CTNNB1, AXIN2, P4HB, DDX3X, CASS4, DMPK, GLI3, MCPH1, WDR19, HNRNPK, SH3PXD2B, PPP2R1A*
0.019366448	27	2449	1.818562505	GO:0120025 Plasma membrane bounded cell projection	*ACTB, DLG1, WDR35, BBS9, IFT81, IGF1R, SPAG17, WDR19, IFT122, IFT140, CDC45, PPP2R1A, GLI3, CBL, CIMIP1, ANTXR1, P4HB, CASR, DIAPH1, NOTCH2, TBX3, CYP17A1, CTNNB1, PTCH1, FLNA, DDX3X, TCTN3*
0.020511102	9	448	3.313734802	GO:0062023 Collagen-containing extracellular matrix	*LTBP1, ADAMTSL4, SHH, FBN1, FREM1, FGFR2, DLG1, BMP7, GPC3*
0.022137837	3	41	12.06953814	GO:0032391 Photoreceptor connecting cilium	*WDR19, IFT140, IFT122*
0.022137837	2	11	29.99097357	GO:0071162 Cmg complex	*CDC45, GINS2*
0.022137837	12	745	2.656918464	GO:0098797 Plasma membrane protein complex	*SCN4A, GNAS, TGFBR1, IGF1R, CTNNB1, ABCC9, DLG1, BMP2, CBL, TGFBR2, FLNA, LRP5*
0.023937847	2	12	27.49172577	GO:0001527 Microfibril	*LTBP1, FBN1*
0.023937847	10	564	2.924651677	GO:0005667 Transcription regulator complex	*SKI, HDAC9, ERCC2, TCF12, CTNNB1, ALX4, GLI3, LHX3, MSX2, RUNX2*
0.023937847	4	91	7.250565038	GO:0016328 Lateral plasma membrane	*PPP2R1A, CTNNB1, DLG1, TSHR*
0.023937847	2	12	27.49172577	GO:0030877 Beta-catenin destruction complex	*AXIN2, CTNNB1*
0.025511052	3	45	10.99669031	GO:0002102 Podosome	*HNRNPK, SH3PXD2B, FLNA*

0.026440043	2	13	25.37697763	GO:0031261 Dna replication preinitiation complex	*CDC45, GINS2*
0.032929249	9	501	2.963180023	GO:0045177 Apical part of cell	*PTCH1, CASR, ASPM, DLG1, GNAS, JAG1, CTSK, NOTCH1, CTNNB1*
0.032929249	13	907	2.364227795	GO:0098552 Side of membrane	*GNAS, IL11RA, P4HB, DLG1, KRAS, TGFBR2, ANTXR1, CTSK, GPC3, ALPL, EFNA4, COLEC11, COLEC10*
0.035371951	2	16	20.61879433	GO:0016600 Flotillin complex	*CBL, CTNNB1*
0.035371951	4	106	6.224541683	GO:0098685 Schaffer collateral-ca1 synapse	*ACTB, NOTCH1, PTPRD, CTNNB1*
0.035371951	10	608	2.712999253	GO:0098978 Glutamatergic synapse	*ACTB, EFNB1, PTPRD, CASR, DLG1, FGFR1, PPP2R1A, NOTCH1, CTNNB1, FLNA*
**Syndromic Genes**					
**Enrichment FDR**	**Number of Genes**	**Pathway Genes**	**Fold Enrichment**	**Pathway**	**Genes**
1.82E-05	4	8	97.72268908	GO:0030991 Intraciliary transport particle a	*WDR35, WDR19, IFT122, IFT140*
2.20E-05	5	25	39.08907563	GO:0030990 Intraciliary transport particle	*WDR35, IFT81, WDR19, IFT122, IFT140*
2.20E-05	6	48	24.43067227	GO:0097542 Ciliary tip	*GLI3, WDR35, IFT81, WDR19, IFT122, IFT140*
3.21E-05	48	4670	2.008860418	GO:0005654 Nucleoplasm	*CDC45, GINS2, KMT2D, ALX4, TCOF1, SMC1A, KAT6A, HUWE1, CTCF, ERCC2, ERF, GLI3, SHOC2, TRIM37, EFTUD2, DPH1, CNOT2, MSX2, RUNX2, MASP1, NOTCH2, TCF12, FTO, CTSK, ZNF462, ZIC1, SKI, RECQL4, NFIA, SPRY1, HNRNPK, ZEB2, CHD7, ESCO2, FAM20C, BCOR, PPP1CB, DDX3X, MCPH1, HDAC9, ACTB, FOXP1, TWIST1, MED13L, TBX3, KAT6B, CTNNB1, ASXL1*
0.000807669	27	2160	2.443067227	GO:0005694 Chromosome	*ACTB, SMC1A, KAT6A, GINS2, TBX3, TCF12, KAT6B, RECQL4, CHD7, ESCO2, CTCF, PPP2R1A, HNRNPK, CTNNB1, ZEB2, PPP1CB, CDC45, LHX3, TRIM37, RUNX2, AHDC1, ALX4, ERF, FOXP1, MSX2, TWIST1, NFIA*
0.000962701	11	433	4.96512508	GO:0043235 Receptor complex	*FGFR2, FGFR3, FGFR1, TGFBR1, NOTCH2, IL11RA, FGFR4, TGFBR2, LRP5, TSHR, FLNA*
0.003049494	9	331	5.314224784	GO:0005788 Endoplasmic reticulum lumen	*P4HB, LTBP1, MBTPS1, ADAMTSL4, GPC3, SHH, FBN1, CRTAP, FAM20C*
0.003929435	2	3	130.2969188	GO:0070021 Transforming growth factor beta ligand-receptor complex	*TGFBR1, TGFBR2*
0.005945509	16	1085	2.882143825	GO:0009986 Cell surface	*ANTXR1, NOTCH2, IL11RA, GPC3, P4HB, FGFR2, EFNB1, TGFBR1, TGFBR2, CASR, FGFR3, CTSK, SHH, TSHR, COLEC11, COLEC10*
0.006227657	2	4	97.72268908	GO:0035517 Pr-dub complex	*ASXL3, ASXL1*
0.006227657	23	1990	2.258916431	GO:1902494 Catalytic complex	*ACTB, KAT6A, GNAS, PPP2R1A, TGFBR1, EFTUD2, KAT6B, KMT2D, AXIN2, ERCC2, SHOC2, TRIM37, TRAF7, HNRNPK, CTNNB1, BCOR, P4HB, PPP1CB, TGFBR2, HDAC9, DPH1, COLEC11, COLEC10*
0.007006329	22	1884	2.28227087	GO:0005794 Golgi apparatus	*MBTPS1, HUWE1, GNPTAB, CHST3, B3GAT3, FGFR3, GNAS, CBL, FGFRL1, FGFR4, SPRY1, SHH, ESCO2, FAM20C, PTCH1, FGFR2, NOTCH2, GPC3, SPAG17, COLEC10, FLNA, KRAS*
0.007442104	13	802	3.168067227	GO:0005813 Centrosome	*IFT81, CEP135, CDC45, RAB23, SKI, CTNNB1, AXIN2, IFT140, DDX3X, ASPM, WDR35, DIAPH1, MCPH1*
0.008164011	13	816	3.113713132	GO:0005929 Cilium	*WDR35, IFT81, SPAG17, WDR19, IFT122, IFT140, CDC45, GLI3, CBL, NOTCH2, TBX3, PTCH1, TCTN3*
0.010030411	14	953	2.871180791	GO:0005815 Microtubule organizing center	*IFT81, CEP135, IFT140, CDC45, RAB23, SKI, IFT122, CTNNB1, AXIN2, DDX3X, ASPM, WDR35, DIAPH1, MCPH1*
0.014425808	18	1515	2.322123305	GO:0000785 Chromatin	*ACTB, KAT6A, TBX3, TCF12, KAT6B, CHD7, ESCO2, HNRNPK, CTNNB1, ZEB2, LHX3, RUNX2, ALX4, ERF, FOXP1, MSX2, TWIST1, NFIA*

0.014425808	10	564	3.465343584	GO:0005667 Transcription regulator complex	*SKI, HDAC9, ERCC2, TCF12, CTNNB1, ALX4, GLI3, LHX3, MSX2, RUNX2*
0.014425808	26	2605	1.950702431	GO:0030054 Cell junction	*ACTB, KRAS, HNRNPK, CTNNB1, P4HB, FLNA, PPP1CB, EFNB1, TGFBR1, DPH1, RAB23, FGFRL1, PTPRD, FGFR4, NFIA, ESCO2, CASR, FGFR1, JAG1, PPP2R1A, CBL, SH3PXD2B, SHC4, PTCH1, FGFR2, FGFR3*
0.014425808	14	996	2.747224191	GO:0070161 Anchoring junction	*ACTB, KRAS, HNRNPK, CTNNB1, P4HB, FLNA, PPP1CB, TGFBR1, FGFRL1, FGFR4, JAG1, CBL, SH3PXD2B, FGFR3*
0.014425808	2	8	48.86134454	GO:0070776 Moz/morf histone acetyltransferase complex	*KAT6A, KAT6B*
0.015107888	18	1532	2.296355618	GO:0015630 Microtubule cytoskeleton	*IFT81, CEP135, IFT140, ASPM, SMC1A, CDC45, ERCC2, RAB23, DIAPH1, SKI, IFT122, CTNNB1, AXIN2, DDX3X, WDR35, MCPH1, SPAG17, PPP2R1A*
0.01671858	9	490	3.589813068	GO:0005925 Focal adhesion	*ACTB, KRAS, HNRNPK, CTNNB1, P4HB, FLNA, PPP1CB, CBL, FGFR3*
0.01671858	2	9	43.43230626	GO:0070775 H3 histone acetyltransferase complex	*KAT6A, KAT6B*
0.016794406	26	2693	1.88695872	GO:0005856 Cytoskeleton	*ACTB, WDR35, IFT81, DIAPH1, SPAG17, CEP135, IFT140, FLNA, ASPM, SMC1A, CDC45, ERCC2, RAB23, SKI, IFT122, CTNNB1, AXIN2, P4HB, DDX3X, DMPK, GLI3, MCPH1, WDR19, HNRNPK, SH3PXD2B, PPP2R1A*
0.017708774	9	500	3.518016807	GO:0030055 Cell-substrate junction	*ACTB, KRAS, HNRNPK, CTNNB1, P4HB, FLNA, PPP1CB, CBL, FGFR3*
0.017967931	25	2572	1.899741234	GO:0042995 Cell projection	*ACTB, WDR35, IFT81, SPAG17, WDR19, IFT122, IFT140, CDC45, PPP2R1A, GLI3, CBL, ANTXR1, P4HB, CASR, DIAPH1, NOTCH2, TBX3, CYP17A1, HNRNPK, CTNNB1, SH3PXD2B, PTCH1, FLNA, DDX3X, TCTN3*
0.018121615	3	41	14.30088133	GO:0032391 Photoreceptor connecting cilium	*WDR19, IFT140, IFT122*
0.018613583	4	92	8.497625137	GO:0005901 Caveola	*TGFBR2, PTCH1, CBL, CTNNB1*
0.019346908	2	11	35.5355233	GO:0071162 Cmg complex	*CDC45, GINS2*
0.019735946	2	12	32.57422969	GO:0001527 Microfibril	*LTBP1, FBN1*
0.019735946	3	45	13.02969188	GO:0002102 Podosome	*HNRNPK, SH3PXD2B, FLNA*
0.019735946	2	12	32.57422969	GO:0030877 Beta-catenin destruction complex	*AXIN2, CTNNB1*
0.019735946	7	329	4.158412301	GO:0045121 Membrane raft	*CBL, TGFBR1, TGFBR2, PTCH1, EFNB1, SHH, CTNNB1*
0.019735946	7	331	4.133285943	GO:0098857 Membrane microdomain	*CBL, TGFBR1, TGFBR2, PTCH1, EFNB1, SHH, CTNNB1*
0.02121658	6	250	4.690689076	GO:0032993 Protein-dna complex	*CDC45, GINS2, ACTB, CTNNB1, KAT6A, KAT6B*
0.021954911	2	13	30.06851972	GO:0031261 Dna replication preinitiation complex	*CDC45, GINS2*
0.025109952	5	180	5.429038282	GO:0036064 Ciliary basal body	*IFT140, CDC45, IFT81, IFT122, WDR35*
0.025109952	16	1417	2.206863832	GO:0098590 Plasma membrane region	*EFNB1, TGFBR2, TSHR, CTNNB1, ANTXR1, PTCH1, CASR, ASPM, GNAS, JAG1, CBL, DIAPH1, CTSK, PTPRD, SHC4, TCTN3*
0.028662554	2	16	24.43067227	GO:0016600 Flotillin complex	*CBL, CTNNB1*
0.028662554	23	2449	1.835542547	GO:0120025 Plasma membrane bounded cell projection	*ACTB, WDR35, IFT81, SPAG17, WDR19, IFT122, IFT140, CDC45, PPP2R1A, GLI3, CBL, ANTXR1, P4HB, CASR, DIAPH1, NOTCH2, TBX3, CYP17A1, CTNNB1, PTCH1, FLNA, DDX3X, TCTN3*
0.028662554	16	1450	2.156638655	GO:0140513 Nuclear protein-containing complex	*CDC45, EFTUD2, GINS2, ASXL3, KMT2D, ASXL1, ERCC2, TRIM37, TCF12, HNRNPK, CTNNB1, BCOR, FLNA, MED13L, HDAC9, ACTB*
0.028662554	2	16	24.43067227	GO:1990909 Wnt signalosome	*CTNNB1, LRP5*

0.035457193	2	18	21.71615313	GO:1905370 Serine-type endopeptidase complex	*COLEC11, COLEC10*
0.038635118	3	64	9.161502101	GO:0008287 Protein serine/threonine phosphatase complex	*PPP2R1A, SHOC2, PPP1CB*
0.038635118	4	129	6.060321803	GO:0044853 Plasma membrane raft	*TGFBR2, PTCH1, CBL, CTNNB1*
0.038635118	3	64	9.161502101	GO:1903293 Phosphatase complex	*PPP2R1A, SHOC2, PPP1CB*
0.039025772	3	65	9.020555915	GO:0031519 Pcg protein complex	*ASXL3, ASXL1, TRIM37*
0.039182711	2	20	19.54453782	GO:1905286 Serine-type peptidase complex	*COLEC11, COLEC10*
0.039793182	12	990	2.369034887	GO:1990234 Transferase complex	*ACTB, KAT6A, TGFBR1, KAT6B, KMT2D, ERCC2, TRIM37, TRAF7, BCOR, TGFBR2, HDAC9, DPH1*
**Nonsyndromic Genes**					
**Enrichment FDR**	**Number of Genes**	**Pathway Genes**	**Fold Enrichment**	**Pathway**	**Genes**
0.049283322	3	199	12.09045226	GO:0005912 Adherens junction	*DLG1, JAG1, NOTCH1*
0.049283322	5	595	6.739495798	GO:0030312 External encapsulating structure	*BMPER, FREM1, DLG1, BMP7, ALPL*
0.049283322	5	594	6.750841751	GO:0031012 Extracellular matrix	*BMPER, FREM1, DLG1, BMP7, ALPL*
0.049283322	4	433	7.408775982	GO:0043235 Receptor complex	*IGF1R, NOTCH1, TGFBR2, BMP2*
0.049283322	7	1417	3.96189132	GO:0098590 Plasma membrane region	*DLG1, BBS9, TGFBR2, JAG1, IGF1R, NOTCH1, SHC4*
0.049283322	3	205	11.73658537	GO:0098802 Plasma membrane signaling receptor complex	*IGF1R, BMP2, TGFBR2*
0.058877173	1	4	200.5	GO:0002193 Maml1-rbp-jkappa- icn1 complex	*NOTCH1*
0.058877173	2	93	17.24731183	GO:0005604 Basement membrane	*DLG1, FREM1*
0.058877173	1	4	200.5	GO:0005899 Insulin receptor complex	*IGF1R*
0.058877173	2	92	17.43478261	GO:0005901 Caveola	*TGFBR2, IGF1R*
0.058877173	1	4	200.5	GO:0035867 Alphav-beta3 integrin-igf-1-igf1r complex	*IGF1R*
0.058877173	1	5	160.4	GO:0043219 Lateral loop	*DLG1*
0.058877173	1	3	267.3333333	GO:0070021 Transforming growth factor beta ligand-receptor complex	*TGFBR2*
0.058877173	1	4	200.5	GO:0070724 Bmp receptor complex	*BMP2*
0.058877173	1	5	160.4	GO:0097025 Mpp7-dlg1-lin7 complex	*DLG1*
0.059311932	12	4675	2.058609626	GO:0005576 Extracellular region	*DLG1, FAM209A, PCK1, ALPL, BMP7, BMP2, BMPER, NELL1, TGFBR2, FREM1, JAG1, NOTCH1*
0.059311932	1	6	133.6666667	GO:0035748 Myelin sheath abaxonal region	*DLG1*
0.059311932	3	329	7.313069909	GO:0045121 Membrane raft	*TGFBR2, DLG1, IGF1R*
0.059311932	3	331	7.268882175	GO:0098857 Membrane microdomain	*TGFBR2, DLG1, IGF1R*

0.060142752	3	339	7.097345133	GO:0045211 Postsynaptic membrane	*DLG1, NOTCH1, SHC4*
0.072533027	2	129	12.43410853	GO:0044853 Plasma membrane raft	*TGFBR2, IGF1R*
0.072533027	2	127	12.62992126	GO:0098839 Postsynaptic density membrane	*DLG1, NOTCH1*
0.08066448	1	11	72.90909091	GO:0034464 Bbsome	*BBS9*
0.08066448	4	745	4.306040268	GO:0098797 Plasma membrane protein complex	*IGF1R, DLG1, BMP2, TGFBR2*
0.080774511	2	146	10.98630137	GO:0042383 Sarcolemma	*DLG1, IGF1R*
0.088917304	3	434	5.543778802	GO:0016324 Apical plasma membrane	*DLG1, JAG1, NOTCH1*
0.088917304	2	160	10.025	GO:0099634 Postsynaptic specialization membrane	*DLG1, NOTCH1*
0.0907627	3	448	5.370535714	GO:0062023 Collagen-containing extracellular matrix	*FREM1, DLG1, BMP7*
0.101617309	3	474	5.075949367	GO:0097060 Synaptic membrane	*DLG1, NOTCH1, SHC4*
0.102940775	2	183	8.765027322	GO:1902911 Protein kinase complex	*IGF1R, TGFBR2*
0.106088677	2	195	8.225641026	GO:0000781 Chromosome telomeric region	*SPO11, RECQL4*
0.106088677	1	20	40.1	GO:0033268 Node of ranvier	*DLG1*
0.106088677	3	501	4.80239521	GO:0045177 Apical part of cell	*DLG1, JAG1, NOTCH1*
0.118200706	1	23	34.86956522	GO:0000242 Pericentriolar material	*BBS9*
0.12420086	3	551	4.366606171	GO:0005911 Cell-cell junction	*DLG1, JAG1, NOTCH1*
0.133635332	4	996	3.220883534	GO:0070161 Anchoring junction	*DLG1, JAG1, NOTCH1, SH3PXD2B*
0.180979233	6	2160	2.227777778	GO:0005694 Chromosome	*SPO11, CTCFL, RECQL4, RTF2, RUNX2, ALX4*

Table S7. GO Molecular Function.

All Genes					
Enrichment FDR	Number of Genes	Pathway Genes	Fold Enrichment	Pathway	Genes
4.40E-09	5	5	164.9503546	GO:0005007 Fibroblast growth factor receptor activity	*FGFR2, FGFR3, FGFR1, FGFRL1, FGFR4*
4.74E-08	10	89	18.53374771	GO:0019199 Transmembrane receptor protein kinase activity	*FGFR2, FGFR3, FGFR1, TGFBR1, FGFRL1, IGF1R, FGFR4, TGFBR2, LTBP1, EFNA4*
2.80E-06	19	648	4.836507311	GO:0008134 Transcription factor binding	*CTNNB1, HDAC9, CNOT2, FOXP1, DDX3X, LHX3, TWIST1, RUNX2, NOTCH2, TBX3, ASXL1, ACTB, KAT6A, CTCF, TCF12, SKI, NFIA, BCOR, FLNA*
3.08E-06	10	146	11.29796949	GO:0019838 Growth factor binding	*FGFR2, FGFR3, FGFR1, FGFRL1, IL11RA, FGFR4, TGFBR2, TGFBR1, IGF1R, LTBP1*
9.11E-06	40	2748	2.401024085	GO:0003677 Dna binding	*ACTB, ALX4, SPO11, SMC1A, CDC45, CTCF, ERCC2, GLI3, LHX3, GLIS3, FOXP1, MSX2, TWIST1, CTCFL, ZBP1, RUNX2, TBX3, ZIC1, SKI, NFIA, ZEB2, CHD7, TCF12, RTF2, KAT6A, ERF, COLEC11, AHDC1, NOTCH2, NOTCH1, RECQL4, KMT2D, DDX3X, HUWE1, ASXL3, ZNF462, KAT6B, HNRNPK, ASXL1, BCOR*
1.32E-05	16	527	5.007980406	GO:0140297 Dna-binding transcription factor binding	*CTNNB1, HDAC9, FOXP1, TWIST1, RUNX2, NOTCH2, TBX3, ASXL1, ACTB, KAT6A, LHX3, TCF12, SKI, NFIA, BCOR, FLNA*
1.86E-05	7	70	16.49503546	GO:0004714 Transmembrane receptor protein tyrosine kinase activity	*FGFR2, FGFR3, FGFR1, FGFRL1, IGF1R, FGFR4, EFNA4*
2.13E-05	5	24	34.36465721	GO:0017134 Fibroblast growth factor binding	*FGFR2, FGFR3, FGFR1, FGFRL1, FGFR4*
3.65E-05	7	80	14.43315603	GO:0046332 Smad binding	*TGFBR1, SKI, TGFBR2, AXIN2, FLNA, TCF12, CTNNB1*
5.07E-05	11	272	6.6707864	GO:0005539 Glycosaminoglycan binding	*NELL1, FGFR1, FGFRL1, FGFR4, TGFBR2, FBN1, FGFR2, BMP7, FGF9, SHH, PTCH1*
5.65E-05	17	693	4.046401195	GO:0003682 Chromatin binding	*ACTB, KAT6A, CDC45, CTCF, CTCFL, ASXL3, KAT6B, CHD7, ASXL1, SMC1A, AHDC1, GLI3, TRIM37, RUNX2, NOTCH1, NFIA, CTNNB1*
0.000114132	28	1772	2.606439012	GO:0005102 Signaling receptor binding	*DLG1, GNAS, EFNB1, BMP7, JAG1, FGF9, TRIM37, CBL, BMP2, ASXL3, TGFBR2, SHH, ASXL1, SHC4, PTCH1, EFNA4, FGFR1, TGFBR1, IGF1R, COLEC10, FLNA, CASR, NOTCH1, PTPRD, FBN1, P4HB, CTNNB1, DIAPH1*
0.000160938	9	199	7.460066289	GO:0008201 Heparin binding	*NELL1, FGFR1, FGFRL1, FGFR4, FBN1, FGFR2, BMP7, FGF9, PTCH1*
0.00018955	23	1326	2.861129831	GO:0060090 Molecular adaptor activity	*LTBP1, JAG1, TGFBR2, KAT6A, KAT6B, KMT2D, CTNNB1, AXIN2, BCOR, TCOF1, TRIM37, CPT1A, AHDC1, NOTCH1, DDX3X, DLG1, MED13L, KRAS, ASXL1, CTCF, ERCC2, HDAC9, CNOT2*
0.00018955	35	2624	2.200176224	GO:0140096 Catalytic activity acting on a protein	*MBTPS1, P4HB, FGFR2, FGFR3, FGFR1, KAT6A, HUWE1, DMPK, TGFBR1, TRIM37, CBL, MASP1, FGFRL1, IGF1R, CTSK, PTPRD, KAT6B, FGFR4, TGFBR2, KMT2D, ESCO2, FAM20C, PPP1CB, HDAC9, PCK1, TRAF7, BCOR, ADCK1, PPP2R1A, SHH, PDILT, MAGEL2, LTBP1, DLG1, EFNA4*
0.000247851	53	4951	1.765778387	GO:0043169 Cation binding	*SLC25A24, CASR, CETP, PCK1, FTO, SHH, KAT6A, COLEC11, MASP1, DIS3L2, SKI, ALPL, FBN1, FAM20C, CYP26B1, HDAC9, LTBP1, SPO11, GNAS, JAG1, CTCF, ERCC2, DMPK, MEGF8, GLI3, TGFBR1, LHX3, GLIS3, TRIM37, DPH1, CBL, GNPTAB, FOXP1, CTCFL, TRAF7, NOTCH2, ASXL3, ZNF462, NOTCH1, CYP17A1, B3GAT3, ZIC1, KAT6B, RECQL4, TGFBR2, FREM1, NELL1, KMT2D, ZEB2, ANTXR1, ESCO2, ASXL1, PPP1CB*
0.000269944	52	4848	1.76926948	GO:0046872 Metal ion binding	*SLC25A24, CASR, PCK1, FTO, SHH, KAT6A, COLEC11, MASP1, DIS3L2, SKI, ALPL, FBN1, FAM20C, CYP26B1, HDAC9, LTBP1, SPO11, GNAS, JAG1, CTCF, ERCC2, DMPK, MEGF8, GLI3, TGFBR1, LHX3, GLIS3, TRIM37, DPH1, CBL, GNPTAB, FOXP1, CTCFL, TRAF7, NOTCH2, ASXL3, ZNF462, NOTCH1, CYP17A1, B3GAT3, ZIC1, KAT6B, RECQL4, TGFBR2, FREM1, NELL1, KMT2D, ZEB2, ANTXR1, ESCO2, ASXL1, PPP1CB*

0.000269944	30	2114	2.340828117	GO:0140110 Transcription regulator activity	*ALX4, KAT6A, CTCF, ERF, GLI3, LHX3, GLIS3, FOXP1, MSX2, TWIST1, RUNX2, TBX3, TCF12, ZIC1, KAT6B, SKI, NFIA, KMT2D, CTNNB1, ZEB2, BCOR, TRIM37, CTCFL, AHDC1, NOTCH2, NOTCH1, MED13L, ASXL1, HDAC9, CNOT2*
0.000446544	33	2521	2.159207339	GO:0097367 Carbohydrate derivative binding	*POR, KRAS, NELL1, FGFR1, DMPK, TGFBR1, PCK1, FGFRL1, FGFR4, TGFBR2, FBN1, SPO11, ADCK1, FGFR2, FGFR3, ABCC9, SMC1A, ACTB, GNAS, BMP7, FGF9, ERCC2, EFTUD2, RAB23, RUNX2, IGF1R, RECQL4, SHH, CHD7, FAM20C, PTCH1, DDX3X, CTSK*
0.000971881	14	636	3.630982649	GO:0004672 Protein kinase activity	*FGFR2, FGFR3, FGFR1, DMPK, TGFBR1, FGFRL1, IGF1R, FGFR4, TGFBR2, FAM20C, PCK1, ADCK1, LTBP1, EFNA4*
0.001025706	16	817	3.230361902	GO:0005509 Calcium ion binding	*SLC25A24, CASR, SHH, COLEC11, MASP1, ALPL, FBN1, LTBP1, JAG1, MEGF8, CBL, GNPTAB, NOTCH2, NOTCH1, NELL1, FAM20C*
0.001251615	47	4465	1.736319522	GO:0003676 Nucleic acid binding	*TCOF1, SMC1A, ACTB, HUWE1, EFTUD2, DIAPH1, RBM38, HNRNPK, P4HB, FLNA, DDX3X, ALX4, SPO11, CDC45, CTCF, ERCC2, GLI3, LHX3, GLIS3, FOXP1, MSX2, TWIST1, CTCFL, ZBP1, RUNX2, TBX3, ZIC1, SKI, NFIA, ZEB2, CHD7, TCF12, RTF2, KAT6A, ERF, COLEC11, AHDC1, NOTCH2, DIS3L2, NOTCH1, RECQL4, KMT2D, ASXL3, ZNF462, KAT6B, ASXL1, BCOR*
0.001452788	7	162	7.127484458	GO:0004713 Protein tyrosine kinase activity	*FGFR2, FGFR3, FGFR1, FGFRL1, IGF1R, FGFR4, EFNA4*
0.001452788	3	13	38.06546645	GO:0005024 Transforming growth factor beta receptor activity	*TGFBR1, TGFBR2, LTBP1*
0.001494807	32	2592	2.036424131	GO:0016740 Transferase activity	*FGFR2, FGFR3, FGFR1, KAT6A, HUWE1, DMPK, TGFBR1, TRIM37, CPT1A, CBL, GNPTAB, CHST3, FGFRL1, IGF1R, B3GAT3, KAT6B, FGFR4, TGFBR2, KMT2D, ESCO2, FAM20C, PCK1, TRAF7, BCOR, ADCK1, DPH1, FTO, SHH, MAGEL2, LTBP1, DLG1, EFNA4*
0.00183604	9	297	4.998495594	GO:0001217 Dna-binding transcription repressor activity	*FOXP1, CTCF, ERF, TWIST1, TBX3, SKI, ZEB2, MSX2, GLIS3*
0.00183604	9	296	5.015382404	GO:1901681 Sulfur compound binding	*NELL1, FGFR1, FGFRL1, FGFR4, FBN1, FGFR2, BMP7, FGF9, PTCH1*
0.002375544	3	16	30.92819149	GO:0070411 I-smad binding	*AXIN2, TGFBR1, CTNNB1*
0.002858473	6	130	7.61309329	GO:0019208 Phosphatase regulator activity	*PPP2R1A, DMPK, BMP2, B3GAT3, ZEB2, SHOC2*
0.003378522	24	1786	2.216578113	GO:0003690 Double-stranded dna binding	*ALX4, CDC45, CTCF, GLI3, LHX3, GLIS3, FOXP1, MSX2, TWIST1, RUNX2, TBX3, ZIC1, SKI, NFIA, ZEB2, TCF12, CTCFL, AHDC1, NOTCH2, NOTCH1, KMT2D, CHD7, BCOR, ZBP1*
0.003378522	15	839	2.949052824	GO:0016301 Kinase activity	*FGFR2, FGFR3, FGFR1, DMPK, TGFBR1, FGFRL1, IGF1R, FGFR4, TGFBR2, FAM20C, PCK1, ADCK1, LTBP1, DLG1, EFNA4*
0.003441541	3	19	26.04479283	GO:0004675 Transmembrane receptor protein serine/threonine kinase activity	*TGFBR1, TGFBR2, LTBP1*
0.003441541	14	751	3.074973322	GO:0016773 Phosphotransferase activity alcohol group as acceptor	*FGFR2, FGFR3, FGFR1, DMPK, TGFBR1, FGFRL1, IGF1R, FGFR4, TGFBR2, FAM20C, PCK1, ADCK1, LTBP1, EFNA4*
0.00457812	11	508	3.571759647	GO:0001228 Dna-binding transcription activator activity rna polymerase ii-specific	*ALX4, LHX3, CTCFL, NOTCH2, TBX3, TCF12, NOTCH1, NFIA, RUNX2, ZIC1, GLIS3*
0.00457812	6	146	6.778781696	GO:0044325 Transmembrane transporter binding	*CASR, ABCC9, DLG1, DIAPH1, CTNNB1, FLNA*
0.004999137	11	515	3.523211458	GO:0001216 Dna-binding transcription activator activity	*ALX4, LHX3, CTCFL, NOTCH2, TBX3, TCF12, NOTCH1, NFIA, RUNX2, ZIC1, GLIS3*
0.005201606	2	5	65.98014184	GO:0036033 Mediator complex binding	*GLI3, SMC1A*
0.005201606	2	5	65.98014184	GO:0043035 Chromatin insulator sequence binding	*CTCF, CTCFL*
0.005201606	2	5	65.98014184	GO:0106260 Dna-dna tethering activity	*AHDC1, CTCF*

0.005201606	2	5	65.98014184	GO:0140587 Chromatin loop anchoring activity	*AHDC1, CTCF*
0.005273271	8	287	4.597919292	GO:0001227 Dna-binding transcription repressor activity rna polymerase ii-specific	*FOXP1, CTCF, ERF, TBX3, SKI, ZEB2, MSX2, GLIS3*
0.005524143	3	24	20.61879433	GO:0050431 Transforming growth factor beta binding	*TGFBR2, TGFBR1, LTBP1*
0.005526121	6	158	6.263937517	GO:0031490 Chromatin dna binding	*ACTB, CTCF, CTCFL, AHDC1, RUNX2, NOTCH1*
0.005859621	23	1785	2.125410732	GO:0043565 Sequence-specific dna binding	*ALX4, CDC45, CTCF, GLI3, LHX3, GLIS3, FOXP1, MSX2, TWIST1, RUNX2, TBX3, ZIC1, SKI, NFIA, ZEB2, TCF12, ERF, CTCFL, NOTCH2, NOTCH1, KMT2D, CHD7, BCOR*
0.005859621	22	1668	2.175604197	GO:1990837 Sequence-specific double-stranded dna binding	*ALX4, CDC45, CTCF, GLI3, LHX3, GLIS3, FOXP1, MSX2, TWIST1, RUNX2, TBX3, ZIC1, SKI, NFIA, ZEB2, TCF12, CTCFL, NOTCH2, NOTCH1, KMT2D, CHD7, BCOR*
0.006160633	16	1024	2.577349291	GO:0016772 Transferase activity transferring phosphorus-containing groups	*FGFR2, FGFR3, FGFR1, DMPK, TGFBR1, GNPTAB, FGFRL1, IGF1R, FGFR4, TGFBR2, FAM20C, PCK1, ADCK1, LTBP1, DLG1, EFNA4*
0.008149011	21	1606	2.156885085	GO:0000976 Transcription cis-regulatory region binding	*ALX4, CTCF, GLI3, LHX3, GLIS3, FOXP1, MSX2, TWIST1, RUNX2, TBX3, ZIC1, SKI, NFIA, ZEB2, TCF12, CTCFL, NOTCH2, NOTCH1, KMT2D, CHD7, BCOR*
0.008149011	21	1607	2.155542904	GO:0001067 Transcription regulatory region nucleic acid binding	*ALX4, CTCF, GLI3, LHX3, GLIS3, FOXP1, MSX2, TWIST1, RUNX2, TBX3, ZIC1, SKI, NFIA, ZEB2, TCF12, CTCFL, NOTCH2, NOTCH1, KMT2D, CHD7, BCOR*
0.008149011	11	565	3.211422833	GO:0003712 Transcription coregulator activity	*KAT6A, KAT6B, KMT2D, CTNNB1, BCOR, TRIM37, NOTCH1, MED13L, ASXL1, HDAC9, CNOT2*

Syndromic Genes

Enrichment FDR	Number of Genes	Pathway Genes	Fold Enrichment	Pathway	Genes
1.73E-09	5	5	195.4453782	GO:0005007 Fibroblast growth factor receptor activity	*FGFR2, FGFR3, FGFR1, FGFRL1, FGFR4*
1.43E-07	19	648	5.73065152	GO:0008134 Transcription factor binding	*CTNNB1, HDAC9, CNOT2, FOXP1, DDX3X, LHX3, TWIST1, RUNX2, NOTCH2, TBX3, ASXL1, ACTB, KAT6A, CTCF, TCF12, SKI, NFIA, BCOR, FLNA*
1.43E-07	9	89	19.76413936	GO:0019199 Transmembrane receptor protein kinase activity	*FGFR2, FGFR3, FGFR1, TGFBR1, FGFRL1, FGFR4, TGFBR2, LTBP1, EFNA4*
1.62E-06	16	527	5.933825523	GO:0140297 Dna-binding transcription factor binding	*CTNNB1, HDAC9, FOXP1, TWIST1, RUNX2, NOTCH2, TBX3, ASXL1, ACTB, KAT6A, LHX3, TCF12, SKI, NFIA, BCOR, FLNA*
6.81E-06	9	146	12.04800276	GO:0019838 Growth factor binding	*FGFR2, FGFR3, FGFR1, FGFRL1, IL11RA, FGFR4, TGFBR2, TGFBR1, LTBP1*
1.13E-05	5	24	40.71778711	GO:0017134 Fibroblast growth factor binding	*FGFR2, FGFR3, FGFR1, FGFRL1, FGFR4*
1.28E-05	35	2748	2.489297029	GO:0003677 Dna binding	*ACTB, ALX4, SMC1A, CDC45, CTCF, ERCC2, GLI3, LHX3, GLIS3, FOXP1, MSX2, TWIST1, RUNX2, TBX3, ZIC1, SKI, NFIA, ZEB2, CHD7, TCF12, KAT6A, ERF, COLEC11, AHDC1, NOTCH2, RECQL4, KMT2D, DDX3X, HUWE1, ASXL3, ZNF462, KAT6B, HNRNPK, ASXL1, BCOR*
1.28E-05	7	80	17.10147059	GO:0046332 Smad binding	*TGFBR1, SKI, TGFBR2, AXIN2, FLNA, TCF12, CTNNB1*
9.45E-05	6	70	16.75246098	GO:0004714 Transmembrane receptor protein tyrosine kinase activity	*FGFR2, FGFR3, FGFR1, FGFRL1, FGFR4, EFNA4*

0.000114899	28	2114	2.588680505	GO:0140110 Transcription regulator activity	*ALX4, KAT6A, CTCF, ERF, GLI3, LHX3, GLIS3, FOXP1, MSX2, TWIST1, RUNX2, TBX3, TCF12, ZIC1, KAT6B, SKI, NFIA, KMT2D, CTNNB1, ZEB2, BCOR, TRIM37, AHDC1, NOTCH2, MED13L, ASXL1, HDAC9, CNOT2*
0.000127597	15	693	4.230419441	GO:0003682 Chromatin binding	*ACTB, KAT6A, CDC45, CTCF, ASXL3, KAT6B, CHD7, ASXL1, SMC1A, AHDC1, GLI3, TRIM37, RUNX2, NFIA, CTNNB1*
0.00015487	21	1326	3.095288794	GO:0060090 Molecular adaptor activity	*LTBP1, JAG1, TGFBR2, KAT6A, KAT6B, KMT2D, CTNNB1, AXIN2, BCOR, TCOF1, TRIM37, CPT1A, AHDC1, DDX3X, MED13L, KRAS, ASXL1, CTCF, ERCC2, HDAC9, CNOT2*
0.000445653	9	272	6.466942659	GO:0005539 Glycosaminoglycan binding	*FGFR1, FGFRL1, FGFR4, TGFBR2, FBN1, FGFR2, FGF9, SHH, PTCH1*
0.000445653	46	4951	1.815893233	GO:0043169 Cation binding	*SLC25A24, CASR, CETP, FTO, SHH, KAT6A, COLEC11, MASP1, DIS3L2, SKI, FBN1, FAM20C, CYP26B1, HDAC9, LTBP1, GNAS, JAG1, CTCF, ERCC2, DMPK, MEGF8, GLI3, TGFBR1, LHX3, GLIS3, TRIM37, DPH1, CBL, GNPTAB, FOXP1, TRAF7, NOTCH2, ASXL3, ZNF462, CYP17A1, B3GAT3, ZIC1, KAT6B, RECQL4, TGFBR2, KMT2D, ZEB2, ANTXR1, ESCO2, ASXL1, PPP1CB*
0.00055113	45	4848	1.814158832	GO:0046872 Metal ion binding	*SLC25A24, CASR, FTO, SHH, KAT6A, COLEC11, MASP1, DIS3L2, SKI, FBN1, FAM20C, CYP26B1, HDAC9, LTBP1, GNAS, JAG1, CTCF, ERCC2, DMPK, MEGF8, GLI3, TGFBR1, LHX3, GLIS3, TRIM37, DPH1, CBL, GNPTAB, FOXP1, TRAF7, NOTCH2, ASXL3, ZNF462, CYP17A1, B3GAT3, ZIC1, KAT6B, RECQL4, TGFBR2, KMT2D, ZEB2, ANTXR1, ESCO2, ASXL1, PPP1CB*
0.00055113	30	2624	2.234512708	GO:0140096 Catalytic activity acting on a protein	*MBTPS1, P4HB, FGFR2, FGFR3, FGFR1, KAT6A, HUWE1, DMPK, TGFBR1, TRIM37, CBL, MASP1, FGFRL1, CTSK, PTPRD, KAT6B, FGFR4, TGFBR2, KMT2D, ESCO2, FAM20C, PPP1CB, HDAC9, TRAF7, BCOR, PPP2R1A, SHH, MAGEL2, LTBP1, EFNA4*
0.000714924	9	297	5.922587217	GO:0001217 Dna-binding transcription repressor activity	*FOXP1, CTCF, ERF, TWIST1, TBX3, SKI, ZEB2, MSX2, GLIS3*
0.000860605	23	1772	2.536819242	GO:0005102 Signaling receptor binding	*GNAS, EFNB1, JAG1, FGF9, TRIM37, CBL, ASXL3, TGFBR2, SHH, ASXL1, SHC4, PTCH1, EFNA4, FGFR1, TGFBR1, COLEC10, FLNA, CASR, PTPRD, FBN1, P4HB, CTNNB1, DIAPH1*
0.001015007	3	13	45.10277957	GO:0005024 Transforming growth factor beta receptor activity	*TGFBR1, TGFBR2, LTBP1*
0.001631425	41	4465	1.794683204	GO:0003676 Nucleic acid binding	*TCOF1, SMC1A, ACTB, HUWE1, EFTUD2, DIAPH1, HNRNPK, P4HB, FLNA, DDX3X, ALX4, CDC45, CTCF, ERCC2, GLI3, LHX3, GLIS3, FOXP1, MSX2, TWIST1, RUNX2, TBX3, ZIC1, SKI, NFIA, ZEB2, CHD7, TCF12, KAT6A, ERF, COLEC11, AHDC1, NOTCH2, DIS3L2, RECQL4, KMT2D, ASXL3, ZNF462, KAT6B, ASXL1, BCOR*
0.001778089	3	16	36.6460084	GO:0070411 I-smad binding	*AXIN2, TGFBR1, CTNNB1*
0.001836528	7	199	6.874963051	GO:0008201 Heparin binding	*FGFR1, FGFRL1, FGFR4, FBN1, FGFR2, FGF9, PTCH1*
0.002170122	28	2592	2.111292665	GO:0016740 Transferase activity	*FGFR2, FGFR3, FGFR1, KAT6A, HUWE1, DMPK, TGFBR1, TRIM37, CPT1A, CBL, GNPTAB, CHST3, FGFRL1, B3GAT3, KAT6B, FGFR4, TGFBR2, KMT2D, ESCO2, FAM20C, TRAF7, BCOR, DPH1, FTO, SHH, MAGEL2, LTBP1, EFNA4*
0.002555642	8	287	5.447954792	GO:0001227 Dna-binding transcription repressor activity rna polymerase ii-specific	*FOXP1, CTCF, ERF, TBX3, SKI, ZEB2, MSX2, GLIS3*
0.002555642	3	19	30.85979655	GO:0004675 Transmembrane receptor protein serine/threonine kinase activity	*TGFBR1, TGFBR2, LTBP1*
0.003052044	27	2521	2.093226977	GO:0097367 Carbohydrate derivative binding	*POR, KRAS, FGFR1, DMPK, TGFBR1, FGFRL1, FGFR4, TGFBR2, FBN1, FGFR2, FGFR3, ABCC9, SMC1A, ACTB, GNAS, FGF9, ERCC2, EFTUD2, RAB23, RUNX2, RECQL4, SHH, CHD7, FAM20C, PTCH1, DDX3X, CTSK*
0.003747867	6	162	7.238717709	GO:0004713 Protein tyrosine kinase activity	*FGFR2, FGFR3, FGFR1, FGFRL1, FGFR4, EFNA4*
0.004247122	21	1786	2.298069956	GO:0003690 Double-stranded dna binding	*ALX4, CDC45, CTCF, GLI3, LHX3, GLIS3, FOXP1, MSX2, TWIST1, RUNX2, TBX3, ZIC1, SKI, NFIA, ZEB2, TCF12, AHDC1, NOTCH2, KMT2D, CHD7, BCOR*

0.004247122	2	5	78.17815126	GO:0036033 Mediator complex binding	*GLI3, SMC1A*
0.004247122	21	1785	2.29935739	GO:0043565 Sequence-specific dna binding	*ALX4, CDC45, CTCF, GLI3, LHX3, GLIS3, FOXP1, MSX2, TWIST1, RUNX2, TBX3, ZIC1, SKI, NFIA, ZEB2, TCF12, ERF, NOTCH2, KMT2D, CHD7, BCOR*
0.004247122	19	1525	2.43505717	GO:0044877 Protein-containing complex binding	*ACTB, GNAS, TGFBR1, TGFBR2, CRTAP, GLI3, KRAS, CTSK, TSHR, ANTXR1, FLNA, DDX3X, CASR, ABCC9, SMC1A, KAT6B, P4HB, PTCH1, FBN1*
0.004247122	3	24	24.43067227	GO:0050431 Transforming growth factor beta binding	*TGFBR2, TGFBR1, LTBP1*
0.004247122	2	5	78.17815126	GO:0106260 Dna-dna tethering activity	*AHDC1, CTCF*
0.004247122	2	5	78.17815126	GO:0140587 Chromatin loop anchoring activity	*AHDC1, CTCF*
0.004372995	13	817	3.109901978	GO:0005509 Calcium ion binding	*SLC25A24, CASR, SHH, COLEC11, MASP1, FBN1, LTBP1, JAG1, MEGF8, CBL, GNPTAB, NOTCH2, FAM20C*
0.004443542	20	1668	2.343469762	GO:1990837 Sequence-specific double-stranded dna binding	*ALX4, CDC45, CTCF, GLI3, LHX3, GLIS3, FOXP1, MSX2, TWIST1, RUNX2, TBX3, ZIC1, SKI, NFIA, ZEB2, TCF12, NOTCH2, KMT2D, CHD7, BCOR*
0.006107961	11	636	3.380344591	GO:0004672 Protein kinase activity	*FGFR2, FGFR3, FGFR1, DMPK, TGFBR1, FGFRL1, FGFR4, TGFBR2, FAM20C, LTBP1, EFNA4*
0.006107961	3	29	20.21848739	GO:0046875 Ephrin receptor binding	*EFNB1, EFNA4, CBL*
0.006369795	5	125	7.817815126	GO:0001221 Transcription coregulator binding	*CNOT2, LHX3, TWIST1, CTNNB1, CTCF*
0.0066852	19	1606	2.312242954	GO:0000976 Transcription cis-regulatory region binding	*ALX4, CTCF, GLI3, LHX3, GLIS3, FOXP1, MSX2, TWIST1, RUNX2, TBX3, ZIC1, SKI, NFIA, ZEB2, TCF12, NOTCH2, KMT2D, CHD7, BCOR*
0.0066852	19	1607	2.310804098	GO:0001067 Transcription regulatory region nucleic acid binding	*ALX4, CTCF, GLI3, LHX3, GLIS3, FOXP1, MSX2, TWIST1, RUNX2, TBX3, ZIC1, SKI, NFIA, ZEB2, TCF12, NOTCH2, KMT2D, CHD7, BCOR*
0.007074269	5	130	7.517129929	GO:0019208 Phosphatase regulator activity	*PPP2R1A, DMPK, B3GAT3, ZEB2, SHOC2*
0.007245529	3	32	18.3230042	GO:0043425 Bhlh transcription factor binding	*TWIST1, RUNX2, TCF12*
0.008023817	10	565	3.459210233	GO:0003712 Transcription coregulator activity	*KAT6A, KAT6B, KMT2D, CTNNB1, BCOR, TRIM37, MED13L, ASXL1, HDAC9, CNOT2*
0.00832976	2	8	48.86134454	GO:0005113 Patched binding	*SHH, PTCH1*
0.00832976	8	376	4.158412301	GO:0061629 Rna polymerase ii-specific dna-binding transcription factor binding	*CTNNB1, HDAC9, FOXP1, NOTCH2, TBX3, ASXL1, ACTB, LHX3*
0.009040424	15	1151	2.547072695	GO:0030674 Protein-macromolecule adaptor activity	*KAT6A, KAT6B, KMT2D, CTNNB1, BCOR, TCOF1, TRIM37, CPT1A, DDX3X, MED13L, KRAS, ASXL1, ERCC2, HDAC9, CNOT2*
0.009358126	7	296	4.622019078	GO:1901681 Sulfur compound binding	*FGFR1, FGFRL1, FGFR4, FBN1, FGFR2, FGF9, PTCH1*
0.009672091	16	1289	2.426009349	GO:0000987 Cis-regulatory region sequence-specific dna binding	*CTCF, GLI3, GLIS3, FOXP1, RUNX2, TBX3, ZIC1, SKI, NFIA, ZEB2, TCF12, TWIST1, NOTCH2, LHX3, MSX2, CHD7*

Nonsyndromic Genes

Enrichment FDR	Number of Genes	Pathway Genes	Fold Enrichment	Pathway	Genes
0.024961188	2	13	123.3846154	GO:0070700 Bmp receptor binding	*BMP2, BMP7*
0.029350017	2	30	53.46666667	GO:0005112 Notch binding	*JAG1, NOTCH1*

0.029350017	2	31	51.74193548	GO:0033612 Receptor serine/threonine kinase binding	*BMP2, BMP7*
0.029350017	2	25	64.16	GO:0070696 Transmembrane receptor protein serine/threonine kinase binding	*BMP2, BMP7*
0.029350017	10	2521	3.181277271	GO:0097367 Carbohydrate derivative binding	*NELL1, PCK1, TGFBR2, SPO11, ADCK1, BMP7, RAB23, RUNX2, IGF1R, RECQL4*
0.030592555	8	1772	3.620767494	GO:0005102 Signaling receptor binding	*DLG1, BMP7, JAG1, BMP2, TGFBR2, SHC4, IGF1R, NOTCH1*
0.030592555	3	166	14.4939759	GO:0008083 Growth factor activity	*BMP2, BMP7, JAG1*
0.030592555	3	158	15.2278481	GO:0031490 Chromatin dna binding	*CTCFL, RUNX2, NOTCH1*
0.034924849	4	515	6.229126214	GO:0001216 Dna-binding transcription activator activity	*ALX4, CTCFL, NOTCH1, RUNX2*
0.034924849	4	508	6.31496063	GO:0001228 Dna-binding transcription activator activity rna polymerase ii-specific	*ALX4, CTCFL, NOTCH1, RUNX2*
0.034924849	1	3	267.3333333	GO:0003918 Dna topoisomerase type ii (double strand cut atp-hydrolyzing) activity	*SPO11*
0.034924849	1	3	267.3333333	GO:0004611 Phosphoenolpyruvate carboxykinase activity	*PCK1*
0.034924849	1	3	267.3333333	GO:0004613 Phosphoenolpyruvate carboxykinase (gtp) activity	*PCK1*
0.034924849	1	3	267.3333333	GO:0005009 Insulin receptor activity	*IGF1R*
0.034924849	1	3	267.3333333	GO:0005010 Insulin-like growth factor receptor activity	*IGF1R*
0.034924849	5	839	4.779499404	GO:0016301 Kinase activity	*IGF1R, TGFBR2, PCK1, ADCK1, DLG1*
0.034924849	3	203	11.85221675	GO:0016853 Isomerase activity	*SPO11, RECQL4, PDILT*
0.034924849	2	54	29.7037037	GO:0017046 Peptide hormone binding	*PTH2R, IGF1R*
0.034924849	5	706	5.679886686	GO:0019904 Protein domain specific binding	*ALX4, RUNX2, SHC4, DLG1, SH3PXD2B*
0.034924849	8	2119	3.027843322	GO:0032553 Ribonucleotide binding	*PCK1, SPO11, ADCK1, RAB23, RUNX2, IGF1R, RECQL4, TGFBR2*
0.034924849	8	2102	3.052331113	GO:0032555 Purine ribonucleotide binding	*PCK1, SPO11, ADCK1, RAB23, RUNX2, IGF1R, RECQL4, TGFBR2*
0.034924849	1	3	267.3333333	GO:0033883 Pyridoxal phosphatase activity	*ALPL*
0.034924849	8	2052	3.126705653	GO:0035639 Purine ribonucleoside triphosphate binding	*PCK1, SPO11, ADCK1, RAB23, RUNX2, IGF1R, RECQL4, TGFBR2*
0.035393277	1	4	200.5	GO:0004035 Alkaline phosphatase activity	*ALPL*
0.035393277	3	272	8.845588235	GO:0005539 Glycosaminoglycan binding	*NELL1, TGFBR2, BMP7*
0.035393277	8	2202	2.913714805	GO:0017076 Purine nucleotide binding	*PCK1, SPO11, ADCK1, RAB23, RUNX2, IGF1R, RECQL4, TGFBR2*
0.035393277	1	4	200.5	GO:0034714 Type iii transforming growth	*TGFBR2*

				factor beta receptor binding	
0.035393277	9	2671	2.702358667	GO:0043168 Anion binding	*PCK1, SPO11, ADCK1, RAB23, RUNX2, IGF1R, RECQL4, TGFBR2, SH3PXD2B*
0.035393277	1	4	200.5	GO:0061821 Telomeric d-loop binding	*RECQL4*
0.035393277	1	4	200.5	GO:0097016 L27 domain binding	*DLG1*
0.035393277	6	1321	3.642694928	GO:0140677 Molecular function activator activity	*BMP7, BMP2, SH3PXD2B, NOTCH1, TGFBR2, JAG1*
0.036930722	2	89	18.02247191	GO:0019199 Transmembrane receptor protein kinase activity	*IGF1R, TGFBR2*
0.037143756	1	5	160.4	GO:0004427 Inorganic diphosphate phosphatase activity	*ALPL*
0.037143756	1	5	160.4	GO:0043035 Chromatin insulator sequence binding	*CTCFL*
0.037143756	1	5	160.4	GO:0043559 Insulin binding	*IGF1R*
0.037143756	1	5	160.4	GO:0062037 D-loop dna binding	*RECQL4*
0.037309617	8	2380	2.695798319	GO:0000166 Nucleotide binding	*PCK1, SPO11, ADCK1, RAB23, RUNX2, IGF1R, RECQL4, TGFBR2*
0.037309617	1	6	133.6666667	GO:0003726 Double-stranded rna adenosine deaminase activity	*ZBP1*
0.037309617	1	6	133.6666667	GO:0009378 Four-way junction helicase activity	*RECQL4*
0.037309617	2	98	16.36734694	GO:0042562 Hormone binding	*PTH2R, IGF1R*
0.037309617	1	6	133.6666667	GO:0043262 Adp phosphatase activity	*ALPL*
0.037309617	1	6	133.6666667	GO:0098919 Structural constituent of postsynaptic density	*DLG1*
0.037309617	8	2398	2.675562969	GO:1901265 Nucleoside phosphate binding	*PCK1, SPO11, ADCK1, RAB23, RUNX2, IGF1R, RECQL4, TGFBR2*
0.037344293	4	636	5.044025157	GO:0004672 Protein kinase activity	*IGF1R, TGFBR2, PCK1, ADCK1*
0.038524246	5	1024	3.916015625	GO:0016772 Transferase activity transferring phosphorus-containing groups	*IGF1R, TGFBR2, PCK1, ADCK1, DLG1*
0.040647694	1	7	114.5714286	GO:0098879 Structural constituent of postsynaptic specialization	*DLG1*
0.043584129	1	8	100.25	GO:0000405 Bubble dna binding	*RECQL4*
0.043584129	1	8	100.25	GO:0003916 Dna topoisomerase activity	*SPO11*
0.043584129	1	8	100.25	GO:1990814 Dna/dna annealing activity	*RECQL4*

Table S8. miRNA prediction using all CS genes.

Gene	ID	Source	p-value	q-value Bonferroni	q-value FDR B&H	q-value FDR B&Y	Hit Count in Query List	Hit Count in Genome
ABCC9	hsa-miR-651-3p	MirDB	5.90E-22	5.00E-18	5.00E-18	4.81E-17	35	1753
ACTB	hsa-miR-362-3p:mirSVR_lowEffct	MicroRNA.org	8.33E-17	7.06E-13	3.53E-13	3.40E-12	30	1785
ADAMTSL4	hsa-miR-425:mirSVR_lowEffct	MicroRNA.org	1.51E-16	1.28E-12	4.26E-13	4.09E-12	30	1825
ADCK1	hsa-miR-605:mirSVR_highEffct	MicroRNA.org	5.03E-16	4.26E-12	1.07E-12	1.02E-11	24	1100
AHDC1	hsa-miR-570:PITA	PITA	9.74E-16	8.25E-12	1.65E-12	1.59E-11	23	1015
ALPL	hsa-miR-335-3p	MirDB	1.32E-15	1.11E-11	1.84E-12	1.77E-11	29	1829
ALX4	hsa-miR-411*:mirSVR_lowEffct	MicroRNA.org	1.52E-15	1.29E-11	1.84E-12	1.77E-11	30	1991
ANTXR1	mmu-miR-6344	MirDB	2.86E-15	2.42E-11	3.03E-12	2.91E-11	23	1068
ASPM	hsa-miR-377:mirSVR_highEffct	MicroRNA.org	4.98E-15	4.22E-11	4.69E-12	4.51E-11	27	1629
ASXL1	hsa-miR-624:mirSVR_lowEffct	MicroRNA.org	5.77E-15	4.89E-11	4.89E-12	4.70E-11	28	1786
ASXL3	mmu-miR-1192	MirDB	9.63E-15	8.16E-11	7.42E-12	7.14E-11	25	1392
AXIN2	mmu-miR-466i-5p	MirDB	1.45E-14	1.23E-10	1.03E-11	9.87E-11	26	1558
B3GAT3	hsa-miR-218-2*:mirSVR_lowEffct	MicroRNA.org	3.28E-14	2.78E-10	2.14E-11	2.06E-10	27	1763
BBS9	hsa-miR-1264:PITA	PITA	3.77E-14	3.19E-10	2.28E-11	2.19E-10	16	453
BCOR	hsa-let-7b-5p:Functional MTI	miRTarbase	4.17E-14	3.54E-10	2.36E-11	2.27E-10	23	1214
BMP2	hsa-miR-497-5p:Functional MTI	miRTarbase	4.76E-14	4.03E-10	2.52E-11	2.43E-10	16	460
BMP7	hsa-miR-4668-3p	MirDB	5.80E-14	4.91E-10	2.73E-11	2.63E-10	20	865
BMPER	hsa-miR-33b:mirSVR_lowEffct	MicroRNA.org	6.54E-14	5.54E-10	2.73E-11	2.63E-10	28	1972
CASR	hsa-miR-424-5p:Functional MTI	miRTarbase	6.84E-14	5.79E-10	2.73E-11	2.63E-10	16	471
CASS4	mmu-miR-12200-5p	MirDB	6.86E-14	5.82E-10	2.73E-11	2.63E-10	24	1379
CBL	mmu-miR-495-3p	MirDB	6.97E-14	5.91E-10	2.73E-11	2.63E-10	24	1380
CDC45	mmu-miR-669f-3p	MirDB	7.42E-14	6.28E-10	2.73E-11	2.63E-10	24	1384
CEP135	mmu-miR-467a-3p	MirDB	7.42E-14	6.28E-10	2.73E-11	2.63E-10	24	1384
CETP	mmu-miR-669b-3p	MirDB	8.01E-14	6.79E-10	2.78E-11	2.68E-10	24	1389
CHD7	hsa-miR-548k:mirSVR_highEffct	MicroRNA.org	8.22E-14	6.96E-10	2.78E-11	2.68E-10	26	1680
CHST3	hsa-miR-1303:mirSVR_lowEffct	MicroRNA.org	9.99E-14	8.46E-10	3.25E-11	3.13E-10	27	1848
CIMIP1	hsa-miR-520d-5p	MirDB	1.29E-13	1.09E-09	4.04E-11	3.88E-10	27	1868
CNOT2	hsa-miR-524-5p	MirDB	1.46E-13	1.24E-09	4.41E-11	4.24E-10	27	1878
COLEC10	hsa-miR-1277-5p	MirDB	2.46E-13	2.09E-09	7.19E-11	6.92E-10	26	1763
COLEC11	mmu-miR-290b-5p	MirDB	4.38E-13	3.71E-09	1.15E-10	1.11E-09	22	1223
CPT1A	hsa-miR-548ah-3p	MirDB	4.58E-13	3.88E-09	1.15E-10	1.11E-09	25	1657
CRTAP	hsa-miR-548am-3p	MirDB	4.58E-13	3.88E-09	1.15E-10	1.11E-09	25	1657
CTCF	hsa-miR-548ap-3p	MirDB	4.75E-13	4.02E-09	1.15E-10	1.11E-09	22	1228
CTCFL	hsa-miR-548t-3p	MirDB	4.75E-13	4.02E-09	1.15E-10	1.11E-09	22	1228
CTNNB1	hsa-miR-548aa	MirDB	4.75E-13	4.02E-09	1.15E-10	1.11E-09	22	1228
CTSK	hsa-miR-548j-3p	MirDB	5.02E-13	4.25E-09	1.16E-10	1.12E-09	25	1664
CYP17A1	hsa-miR-548ae-3p	MirDB	5.22E-13	4.42E-09	1.16E-10	1.12E-09	25	1667
CYP21B	hsa-miR-548aq-3p	MirDB	5.22E-13	4.42E-09	1.16E-10	1.12E-09	25	1667
CYP26B1	hsa-miR-9-5p	MirDB	5.71E-13	4.84E-09	1.24E-10	1.19E-09	20	980

DDX3X	mmu-miR-466k	MirDB	9.17E-13	7.77E-09	1.92E-10	1.85E-09	24	1558
DIAPH1	mmu-miR-466d-5p	MirDB	9.29E-13	7.87E-09	1.92E-10	1.85E-09	24	1559
DIS3L2	hsa-miR-3714	MirDB	1.01E-12	8.52E-09	2.03E-10	1.95E-09	20	1011
DLG1	hsa-miR-374b:PITA	PITA	1.09E-12	9.19E-09	2.09E-10	2.01E-09	18	776
DMPK	hsa-miR-374a:PITA	PITA	1.09E-12	9.19E-09	2.09E-10	2.01E-09	18	776
DPH1	hsa-miR-548p	MirDB	1.29E-12	1.09E-08	2.41E-10	2.32E-09	22	1292
EFNA4	hsa-miR-8485	MirDB	1.31E-12	1.11E-08	2.41E-10	2.32E-09	25	1739
EFNB1	hsa-miR-466:mirSVR_highEffct	MicroRNA.org	1.64E-12	1.39E-08	2.96E-10	2.85E-09	26	1918
EFTUD2	hsa-miR-548m:mirSVR_highEffct	MicroRNA.org	2.25E-12	1.91E-08	3.97E-10	3.82E-09	25	1783
ERCC2	hsa-miR-200b:mirSVR_highEffct	MicroRNA.org	2.48E-12	2.10E-08	4.28E-10	4.12E-09	25	1791
ERF	hsa-miR-331-5p:mirSVR_highEffct	MicroRNA.org	3.17E-12	2.69E-08	5.37E-10	5.17E-09	18	828
ESCO2	hsa-miR-20a*:mirSVR_highEffct	MicroRNA.org	3.76E-12	3.19E-08	6.25E-10	6.01E-09	23	1513
FAM209A	hsa-miR-561:PITA	PITA	4.44E-12	3.76E-08	7.23E-10	6.95E-09	19	968
FAM20C	hsa-miR-570-3p	MirDB	4.89E-12	4.15E-08	7.82E-10	7.53E-09	24	1688
FBN1	hsa-miR-767-5p:mirSVR_lowEffct	MicroRNA.org	5.16E-12	4.37E-08	8.09E-10	7.79E-09	23	1537
FGF9	hsa-miR-624*:mirSVR_lowEffct	MicroRNA.org	5.30E-12	4.49E-08	8.17E-10	7.86E-09	21	1246
FGFR1	hsa-miR-589*:mirSVR_highEffct	MicroRNA.org	5.71E-12	4.84E-08	8.49E-10	8.17E-09	25	1862
FGFR2	hsa-let-7c-3p	MirDB	5.77E-12	4.89E-08	8.49E-10	8.17E-09	20	1114
FGFR3	hsa-miR-548x-3p	MirDB	5.91E-12	5.01E-08	8.49E-10	8.17E-09	25	1865
FGFR4	hsa-miR-548aj-3p	MirDB	5.91E-12	5.01E-08	8.49E-10	8.17E-09	25	1865
FGFRL1	hsa-miR-95-5p	MirDB	6.21E-12	5.27E-08	8.75E-10	8.42E-09	22	1401
FLNA	mmu-miR-7229-3p	MirDB	6.30E-12	5.34E-08	8.75E-10	8.42E-09	19	988
FOXP1	hsa-miR-16-5p:Functional MTI	miRTarbase	6.68E-12	5.66E-08	9.13E-10	8.79E-09	23	1557
FREM1	hsa-miR-98-3p	MirDB	7.13E-12	6.04E-08	9.28E-10	8.93E-09	22	1411
FTO	hsa-miR-2052:mirSVR_highEffct	MicroRNA.org	7.22E-12	6.11E-08	9.28E-10	8.93E-09	23	1563
GINS2	hsa-let-7f-1-3p	MirDB	7.23E-12	6.12E-08	9.28E-10	8.93E-09	22	1412
GLI3	hsa-let-7b-3p	MirDB	7.23E-12	6.12E-08	9.28E-10	8.93E-09	22	1412
GLIS3	hsa-let-7a-3p	MirDB	7.43E-12	6.29E-08	9.29E-10	8.94E-09	22	1414
GNAS	hsa-miR-429:mirSVR_highEffct	MicroRNA.org	7.69E-12	6.51E-08	9.29E-10	8.94E-09	25	1888
GNPTAB	hsa-miR-1237:PITA	PITA	7.70E-12	6.53E-08	9.29E-10	8.94E-09	11	214
GPC3	hsa-miR-3125:mirSVR_highEffct	MicroRNA.org	7.76E-12	6.58E-08	9.29E-10	8.94E-09	24	1726
HDAC9	hsa-miR-4753-3p	MirDB	7.78E-12	6.59E-08	9.29E-10	8.94E-09	21	1272
HNRNPK	hsa-miR-15b-5p:Functional MTI	miRTarbase	8.46E-12	7.17E-08	9.96E-10	9.58E-09	17	760
HUWE1	hsa-miR-371-5p:mirSVR_highEffct	MicroRNA.org	9.66E-12	8.19E-08	1.12E-09	1.08E-08	19	1013
IFT122	mmu-miR-7234-5p	MirDB	1.04E-11	8.81E-08	1.19E-09	1.15E-08	21	1292
IFT140	hsa-miR-582-5p:TargetScan	TargetScan	1.06E-11	8.98E-08	1.20E-09	1.15E-08	14	457
IFT81	hsa-miR-615-3p:Functional MTI	miRTarbase	1.10E-11	9.28E-08	1.22E-09	1.18E-08	18	893
IGF1R	mmu-miR-32-3p	MirDB	1.16E-11	9.79E-08	1.27E-09	1.22E-08	15	557
IL11RA	mmu-let-7c-2-3p	MirDB	1.31E-11	1.11E-07	1.35E-09	1.30E-08	19	1031
JAG1	mmu-let-7a-1-3p	MirDB	1.31E-11	1.11E-07	1.35E-09	1.30E-08	19	1031
KAT6A	hsa-miR-211-5p:TargetScan	TargetScan	1.31E-11	1.11E-07	1.35E-09	1.30E-08	15	562

KAT6B	hsa-miR-204-5p:TargetScan	TargetScan	1.31E-11	1.11E-07	1.35E-09	1.30E-08	15	562
KMT2D	mmu-miR-98-3p	MirDB	1.33E-11	1.12E-07	1.35E-09	1.30E-08	19	1032
KRAS	hsa-miR-150:mirSVR_lowEffct	MicroRNA.org	1.36E-11	1.15E-07	1.35E-09	1.30E-08	25	1939
LHX3	hsa-miR-548bb-3p	MirDB	1.36E-11	1.15E-07	1.35E-09	1.30E-08	22	1459
LRP5	mmu-miR-3094-3p	MirDB	1.36E-11	1.15E-07	1.35E-09	1.30E-08	22	1459
LTBP1	mmu-let-7f-1-3p	MirDB	1.42E-11	1.20E-07	1.38E-09	1.33E-08	19	1036
MAGEL2	mmu-let-7b-3p	MirDB	1.42E-11	1.20E-07	1.38E-09	1.33E-08	19	1036
MASP1	hsa-miR-4668-5p	MirDB	1.47E-11	1.24E-07	1.41E-09	1.36E-08	20	1174
MBTPS1	hsa-miR-548ac	MirDB	1.59E-11	1.35E-07	1.47E-09	1.41E-08	22	1471
MCPH1	hsa-miR-524-5p:PITA	PITA	1.60E-11	1.36E-07	1.47E-09	1.41E-08	18	914
MED13L	hsa-miR-520d-5p:PITA	PITA	1.60E-11	1.36E-07	1.47E-09	1.41E-08	18	914
MEGF8	hsa-miR-548h-3p	MirDB	1.61E-11	1.36E-07	1.47E-09	1.41E-08	22	1472
MSX2	hsa-miR-548z	MirDB	1.61E-11	1.36E-07	1.47E-09	1.41E-08	22	1472
MYADML2	hsa-miR-3977	MirDB	1.72E-11	1.46E-07	1.55E-09	1.49E-08	14	474
NELL1	hsa-miR-548d-3p	MirDB	1.76E-11	1.49E-07	1.55E-09	1.49E-08	22	1479
NFIA	hsa-miR-603	MirDB	1.77E-11	1.50E-07	1.55E-09	1.49E-08	14	475
NOTCH1	hsa-miR-548ar-3p	MirDB	1.78E-11	1.51E-07	1.55E-09	1.49E-08	21	1330
NOTCH2	hsa-miR-548e-3p	MirDB	2.10E-11	1.78E-07	1.82E-09	1.75E-08	20	1198
OSTM1	hsa-miR-6504-3p	MirDB	2.20E-11	1.86E-07	1.88E-09	1.81E-08	18	932
P4HB	hsa-miR-320a:Functional MTI	miRTarbase	2.24E-11	1.90E-07	1.90E-09	1.83E-08	15	584
PCK1	hsa-miR-548bc	MirDB	2.26E-11	1.92E-07	1.90E-09	1.83E-08	20	1203
PDILT	hsa-miR-548f-3p	MirDB	2.37E-11	2.00E-07	1.97E-09	1.89E-08	20	1206
POR	hsa-miR-12136	MirDB	2.40E-11	2.04E-07	1.98E-09	1.90E-08	25	1992
PPP1CB	hsa-miR-548az-3p	MirDB	2.47E-11	2.09E-07	2.01E-09	1.94E-08	20	1209
PPP2R1A	hsa-miR-218-5p:Non-Functional MTI	miRTarbase	2.56E-11	2.17E-07	2.06E-09	1.98E-08	17	816
PTCH1	hsa-miR-139-5p:TargetScan	TargetScan	2.66E-11	2.26E-07	2.13E-09	2.05E-08	12	314
PTH2R	hsa-miR-1262:mirSVR_lowEffct	MicroRNA.org	2.69E-11	2.28E-07	2.13E-09	2.05E-08	22	1512
PTPRD	hsa-miR-618:mirSVR_highEffct	MicroRNA.org	2.74E-11	2.32E-07	2.15E-09	2.07E-08	19	1077
RAB23	hsa-miR-625*:mirSVR_lowEffct	MicroRNA.org	3.11E-11	2.63E-07	2.41E-09	2.32E-08	21	1371
RAB5IF	hsa-miR-200a-3p	MirDB	3.65E-11	3.09E-07	2.81E-09	2.70E-08	17	835
RBM38	hsa-miR-141-3p	MirDB	3.85E-11	3.27E-07	2.94E-09	2.83E-08	17	838
RECQL4	hsa-miR-1245:mirSVR_lowEffct	MicroRNA.org	3.89E-11	3.30E-07	2.94E-09	2.83E-08	21	1388
RNU12	hsa-miR-329:mirSVR_lowEffct	MicroRNA.org	4.29E-11	3.64E-07	3.22E-09	3.10E-08	23	1710
RTF2	hsa-miR-548an	MirDB	4.71E-11	3.99E-07	3.50E-09	3.36E-08	19	1112
RUNX2	hsa-miR-92a-3p:Functional MTI (Weak)	miRTarbase	4.99E-11	4.23E-07	3.68E-09	3.54E-08	21	1407
SCN4A	hsa-miR-34a-5p:Functional MTI (Weak)	miRTarbase	5.36E-11	4.54E-07	3.92E-09	3.77E-08	16	735
SH3PXD2B	hsa-miR-3658	MirDB	5.56E-11	4.71E-07	4.03E-09	3.87E-08	22	1571
SHC4	hsa-miR-217:mirSVR_highEffct	MicroRNA.org	6.21E-11	5.27E-07	4.46E-09	4.29E-08	20	1274
SHH	hsa-miR-10399-5p	MirDB	6.74E-11	5.71E-07	4.77E-09	4.59E-08	17	869
SHOC2	hsa-miR-129-5p:mirSVR_highEffct	MicroRNA.org	6.76E-11	5.73E-07	4.77E-09	4.59E-08	23	1750
SKI	hsa-miR-200b-3p:TargetScan	TargetScan	7.11E-11	6.02E-07	4.90E-09	4.71E-08	17	872

SLC25A24	hsa-miR-200c-3p:TargetScan	TargetScan	7.11E-11	6.02E-07	4.90E-09	4.71E-08	17	872
SMC1A	hsa-miR-429:TargetScan	TargetScan	7.11E-11	6.02E-07	4.90E-09	4.71E-08	17	872
SPAG17	hsa-miR-6838-5p:Functional MTI (Weak)	miRTarbase	7.23E-11	6.13E-07	4.94E-09	4.75E-08	13	431
SPO11	hsa-miR-583:mirSVR_highEffct	MicroRNA.org	7.33E-11	6.21E-07	4.97E-09	4.78E-08	21	1437
SPRY1	hsa-miR-4455	MirDB	7.44E-11	6.30E-07	5.00E-09	4.81E-08	13	432
TBX3	hsa-miR-182:mirSVR_lowEffct	MicroRNA.org	7.79E-11	6.60E-07	5.18E-09	4.99E-08	24	1932
TCF12	hsa-miR-195-5p:Functional MTI	miRTarbase	7.83E-11	6.64E-07	5.18E-09	4.99E-08	15	639
TCOF1	hsa-miR-191:mirSVR_highEffct	MicroRNA.org	8.14E-11	6.90E-07	5.25E-09	5.05E-08	12	346
TCTN3	hsa-miR-1185-2-3p	MirDB	8.18E-11	6.93E-07	5.25E-09	5.05E-08	17	880
TGFBR1	hsa-miR-1185-1-3p	MirDB	8.18E-11	6.93E-07	5.25E-09	5.05E-08	17	880
TGFBR2	hsa-miR-548n:PITA	PITA	8.18E-11	6.93E-07	5.25E-09	5.05E-08	18	1011
TRAF7	hsa-miR-641:mirSVR_highEffct	MicroRNA.org	8.39E-11	7.10E-07	5.34E-09	5.14E-08	24	1939
TRIM37	hsa-miR-141:mirSVR_highEffct	MicroRNA.org	9.82E-11	8.32E-07	6.21E-09	5.97E-08	22	1619
TRPM3	hsa-miR-32:mirSVR_lowEffct	MicroRNA.org	1.15E-10	9.72E-07	7.20E-09	6.92E-08	23	1798
TSHR	hsa-miR-4262:mirSVR_highEffct	MicroRNA.org	1.20E-10	1.01E-06	7.46E-09	7.18E-08	23	1802
TWIST1	hsa-miR-485-3p:mirSVR_lowEffct	MicroRNA.org	1.29E-10	1.09E-06	7.99E-09	7.68E-08	23	1809
WDR19	hsa-miR-140-5p:PITA	PITA	1.48E-10	1.26E-06	9.09E-09	8.75E-08	11	282
WDR35	hsa-miR-3167:mirSVR_lowEffct	MicroRNA.org	1.50E-10	1.27E-06	9.16E-09	8.82E-08	24	1996
ZBP1	hsa-miR-1275:mirSVR_lowEffct	MicroRNA.org	1.53E-10	1.30E-06	9.27E-09	8.92E-08	23	1825
ZEB2	hsa-miR-1283	MirDB	1.55E-10	1.31E-06	9.29E-09	8.94E-08	18	1052
ZIC1	hsa-miR-369-3p:TargetScan	TargetScan	1.65E-10	1.40E-06	9.84E-09	9.46E-08	13	461
ZNF462	hsa-miR-19b:mirSVR_highEffct	MicroRNA.org	1.70E-10	1.44E-06	9.93E-09	9.56E-08	21	1505

Table S9. miRNAs predicted from CS associated genes

miRNA	*p* value	*q* value FDR B&H*	Gene Number	Target genes
**A. miRNAs from all CS genes**				
hsa-miR-651-3p	5.90×10^{-22}	5.00×10^{-18}	34	*ABCC9*, *ALX4*, *BCOR*, *CBL*, *CNOT2*, *CPT1A*, *DLG1*, *FBN1*, *HDAC9*, *HNRNPK*, *KAT6A*, KAT6B, KRAS, *MCPH1*, *MED13L*, *MSX2*, *NFIA*, *NOTCH2*, *OSTM1*, *PCK1*, *PPP1CB*, *PTH2R*, *RAB23*, *SH3PXD2B*, *SKI*, *SLC25A24*, *SMC1A*, *SPO11*, *SPRY1*, *TBX3*, *TCF12*, *TGFBR1*, *TSHR*, *ZEB2*
hsa-miR-362-3p	8.33×10^{-17}	3.53×10^{-13}	30	*ABCC9*, *ASXL1*, *BBS9*, *BMP2*, *BMP7*, *BMPER*, *CASS4*, *CNOT2*, *CTCF*, *CYP26B1*, *DLG1*, *DIS3L2*, *DMPK*, *FBN1*, *IFT122*, *IL11RA*, *KMT2D*, *KRAS*, *NFIA*, *OSTM1*, *PPP1CB*, *PPP2R1A*, *PTCH1*, *PTH2R*, *PTPRD*, *RAB23*, *SCN4A*, *SH3PXD2B*, *TBX3*, *ZIC1*
hsa-miR-425	1.51×10^{-16}	4.26×10^{-13}	30	*ABCC9*, *ANTXR1*, *ASPM*, *AXIN2*, *BMPER*, *CBL*, *CEP135*, *CHD7*, *DLG1*, *FAM20C*, *FBN1*, *FGF9*, *GLIS3*, *GPC3*, *HDAC9*, *IFT81*, *KRAS*, *MED13L*, *NOTCH1*, *NOTCH2*, *PCK1*, *PPP2R1A*, *RAB5IF*, *SCN4A*, *SPAG17*, *SPO11*, *SPRY1*, *TCF12*, *TRPM3*, *TWIST1*
hsa-miR-605	5.03×10^{-16}	1.07×10^{-12}	24	*ASXL1*, *BBS9*, *CASS4*, *CEP135*, *DIS3L2*, *DLG1*, *EFTUD2*, *ESCO2*, *FGF9*, *FOXP1*, *GLI3*, *GLIS3*, *HNRNPK*, *KAT6B*, *LRP5*, *LTBP1*, *MASP1*, *NOTCH2*, *P4HB*, *PTCH1*, *RUNX2*, *TRPM3*, *WDR35*, *ZEB2*
hsa-miR-570	9.47×10^{-16}	1.65×10^{-12}	23	*AHDC1*, *BMP2*, *CEP135*, *DDX3X*, *GLIS3*, *GPC3*, *IGF1R*, *KAT6B*, *KMT2D*, *KRAS*, *MED13L*, *POR*, *PPP1CB*, *PTCH1*, *PTPRD*, *RAB5IF*, *RBM38*, *RUNX2*, *SPRY1*, *TBX3*, *TCF12*, *TGFBR1*, *ZIC1*
hsa-miR-335-3p	1.32×10^{-15}	1.84×10^{-12}	29	*ABCC9*, *AHDC1*, *ASXL3*, *BMP7*, *BCOR*, *CASR*, *CEP135*, *CNOT2*, *CPT1A*, *CYP26B1*, *DIAPH1*, *DLG1*, *FGF9*, *FOXP1*, *GLIS3*, *HDAC9*, *HNRNPK*, *IFT81*, *JAG1*, *KRAS*, *LTBP1*, *MASP1*, *MCPH1*, *NFIA*, *OSTM1*, *SHH*, *SLC25A24*, *WDR35*, *ZEB2*
hsa-miR-411	1.52×10^{-15}	1.84×10^{-12}	29	*ASXL1*, *BBS9*, *BCOR*, *CHD7*, *CNOT2*, *COLEC10*, *CTCF*, *CTSK*, *FBN1*, *FGFR1*, *FGFR2*, *FGFR3*, *GLI3*, *IFT122*, *IGF1R*, *KAT6B*, *LTBP1*, *NOTCH2*, *OSTM1*, *PCK1*, *PTCH1*, *RAB23*, *RUNX2*, *SHOC2*, *TCF12*, *TGFBR2*, *TRIM37*, *WDR35*, *ZEB2*
hsa-miR-377	4.98×10^{-15}	4.69×10^{-12}	28	*ABCC9*, *ASXL3*, *CEP135*, *CHD7*, *CHST3*, *CTCF*, *CTNNB1*, *FGFR1*, *FOXP1*, *GINS2*, *GNAS*, *GNPTAB*, *HDAC9*, *IL11RA*, *JAG1*, *KAT6A*, *KAT6B*, *MED13L*, *NELL1*, *NOTCH2*, *PTCH1*, *RUNX2*, *SLC25A24*, *TCF12*, *TRPM3*, *TRIM37*, *ZEB2*, *ZNF462*
hsa-miR-624	5.77×10^{-15}	4.89×10^{-12}	28	*ABCC9*, *ANTXR1*, *ASXL1*, *ASXL3*, *AXIN2*, *BBS9*, *BCOR*, *CASS4*, *CHD7*, *CNOT2*, *CPT1A*, *EFTUD2*, *FBN1*, *FGFR2*, *FREM1*, *GNPTAB*, *HNRNPK*, *HUWE1*, *JAG1*, *KRAS*, *MCPH1*, *RAB5IF*, *RBM38*, *SHOC2*, *TCTN3*, *WDR19*, *WDR35*, *ZEB2*
hsa-miR-218-2	3.28×10^{-14}	2.14×10^{-11}	27	*BBS9*, *BCOR*, *BMP2*, *CASR*, *CEP135*, *CTCFL*, *DLG1*, *DPH1*, *ERF*, *FBN1*, *FOXP1*, *GLIS3*, *GNPTAB*, *GPC3*, *HUWE1*, *KAT6B*, *KRAS*, *MCPH1*, *MED13L*, *PTPRD*, *RAB23*, *SHOC2*, *SMC1A*, *TBX3*, *TCF12*, *TRPM3*, *ZIC1*
**B. miRNAs from syndromic CS genes**				
hsa-miR-651-3p	2.89×10^{-20}	2.40×10^{-16}	31	*ABCC9*, *ALX4*, *BCOR*, *CBL*, *CNOT2*, *CPT1A*, *FBN1*, *FOXP1*, *HDAC9*, *HNRNPK*, *KAT6A*, *KAT6B*, *KRAS*, *MCPH1*, *MED13L*, *MSX2*, *SH3PXD2B*, *NFIA*, *NOTCH2*, *OSTM1*, *PPP1CB*, *RAB23*, *SKI*, *SLC25A24*, *SMC1A*, *SPRY1*, *TBX3*, *TCF12*, *TGFBR1*, *TSHR*, *ZEB2*
hsa-miR-377	5.71×10^{-16}	2.37×10^{-12}	26	*ABCC9*, *ASXL3*, *CEP135*, *CHD7*, *CTCF*, *CTNNB1*, *FGFR1*, *FOXP1*, *GINS2*, *GNAS*, *GNPTAB*, *HDAC9*, *IL11RA*, *JAG1*, *KAT6A*, *KAT6B*, *MED13L*, *NOTCH2*, *PTCH1*, *RUNX2*, *SLC25A24*, *TCF12*, *TRPM3*, *TRIM37*, *ZEB2*, *ZNF462*
hsa-miR-335-3p	9.64×10^{-16}	2.67×10^{-12}	27	*ABCC9*, *AHDC1*, *ASXL3*, *BCOR*, *CASR*, *CEP135*, *CNOT2*, *CPT1A*, *CYP26B1*, *DIAPH1*, *FGF9*, *FOXP1*, *GLIS3*, *HDAC9*, *HNRNPK*, *IFT81*, *JAG1*, *KRAS*, *LTBP1*, *MASP1*, *MCPH1*, *NFIA*, *OSTM1*, *SLC25A24*, *SHH*, *WDR35*, *ZEB2*
hsa-miR-425-5p	4.61×10^{-15}	9.56×10^{-12}	16	*AXIN2*, *CYP26B1*, *DDX3X*, *DIAPH1*, *DMPK*, *EFTUD2*, *FGFR1*, *FGFR4*, *KMT2D*, *NFIA*, *NOTCH2*, *PTCH1*, *PTPRD*, *RAB23*, *SHOC2*, *SKI*
hsa-miR-605	1.63×10^{-14}	2.71×10^{-11}	21	*ASXL1*, *CEP135*, *DIS3L2*, *EFTUD2*, *ESCO2*, *FGF9*, *FOXP1*, *GLI3*, *GLIS3*, *HNRNPK*, *KAT6B*, *LRP5*, *LTBP1*, *MASP1*, *NOTCH2*, *RUNX2*, *P4HB*, *PTCH1*, *TRPM3*, *WDR35*, *ZEB2*
hsa-miR-570	4.09×10^{-14}	5.65×10^{-11}	20	*AHDC1*, *CEP135*, *DDX3X*, *GLIS3*, *GPC3*, *KAT6B*, *KMT2D*, *KRAS*, *MED13L*, *POR*, *PPP1CB*, *SPRY1*, *PTCH1*, *PTPRD*, *RAB5IF*, *RUNX2*, *TBX3*, *TCF12*, *TGFBR1*, *ZIC1*
hsa-miR-33b	4.88×10^{-14}	5.75×10^{-11}	26	*ACTB*, *ANTXR1*, *CTNNB1*, *CTCF*, *DDX3X*, *ESCO2*, *FBN1*, *FGFR2*, *FOXP1*, *GLIS3*, *GNPTAB*, *HUWE1*, *IFT81*, *KAT6B*, *MBTPS1*, *MCPH1*, *MED13L*, *NFIA*, *RUNX2*, *SHOC2*, *SKI*, *TBX3*, *TCF12*, *TGFBR1*, *TRPM3*, *TWIST1*
hsa-miR-411	6.08×10^{-14}	5.74×10^{-11}	26	*ASXL1*, *BCOR*, *CHD7*, *COLEC10*, *CTCF*, *RAB23*, *CNOT2*, *CTSK*, *FBN1*, *FGFR1*, *FGFR2*, *FGFR3*, *GLI3*, *IFT122*, *KAT6B*, *LTBP1*, *NOTCH2*, *TCF12*, *OSTM1*, *PTCH1*, *RUNX2*, *SHOC2*, *TGFBR2*, *TRIM37*, *WDR35*, *ZEB2*

hsa-miR-497-5p	6.25×10^{-14}	5.75×10^{-11}	15	*AXIN2*, *CYP26B1*, *DDX3X*, *DIAPH1*, *DMPK*, *EFTUD2*, *FGFR4*, *KMT2D*, *NOTCH2*, *PTPRD*, *RAB23*, *RUNX2*, *SHOC2*, *SKI*, *TWIST1*
hsa-miR-425	6.93×10^{-14}	5.75×10^{-11}	25	*ABCC9*, *ANTXR1*, *ASPM*, *AXIN2*, *CBL*, *CEP135*, *CHD7*, *FAM20C*, *FBN1*, *FGF9*, *GLIS3*, *GPC3*, *HDAC9*, *IFT81*, *KRAS*, *MED13L*, *NOTCH2*, *PPP2R1A*, *RAB5IF*, *SCN4A*, *SPAG17*, *SPRY1*, *TCF12*, *TRPM3*, *TWIST1*
**C. miRNAs from nonsyndromic CS genes**				
hsa-miR-371-5p	5.78×10^{-7}	2.00×10^{-3}	7	*ADCK1*, *BBS9*, *CTCFL*, *DLG1*, *JAG1*, *PTH2R*, *ZBP1*
hsa-miR-329	1.49×10^{-6}	2.00×10^{-3}	8	*BMP2*, *BMP7*, *BMPER*, *DLG1*, *BBS9*, *PTH2R*, *RAB23*, *SH3PXD2B*
hsa-miR-204-5p	1.57×10^{-6}	2.00×10^{-3}	5	*ALPL*, *BBS9*, *NOTCH1*, *RUNX2*, *TGFBR2*
hsa-miR-362-3p	2.05×10^{-6}	2.00×10^{-1}	8	*BBS9*, *BMP2*, *BMP7*, *BMPER*, *DLG1*, *PTH2R*, *RAB23*, *SH3PXD2B*
hsa-miR-379-3p	3.82×10^{-6}	2.00×10^{-3}	5	*DLG1*, *IGF1R*, *RAB23*, *SPO11*, *TGFBR2*
hsa-miR-411-3p	3.86×10^{-6}	2.00×10^{-3}	5	*DLG1*, *IGF1R*, *RAB23*, *SPO11*, *TGFBR2*
hsa-miR-31	4.13×10^{-6}	2.00×10^{-3}	6	*BBS9*, *BMPER*, *DLG1*, *RAB23*, *SHC4*, *SPO11*
hsa-miR-625	4.28×10^{-6}	2.00×10^{-3}	7	*BBS9*, *BMPER*, *JAG1*, *PTH2R*, *RTF2*, *RUNX2*, *SHC4*
hsa-miR-584-5p	9.74×10^{-6}	4.14×10^{-3}	4	*FREM1*, *JAG1*, *RAB23*, *RUNX2*
hsa-miR-432-5p	1.71×10^{-5}	5.36×10^{-3}	4	*ADCK1*, *ALPL*, *IGF1R*, *TGFBR2*

*B&H represents the Benjamini-Hochberg (BH) procedure.

Table S10. miRNA prediction using syndromic CS genes.

Gene	ID	Source	p-value	q-value Bonferroni	q-value FDR B&H	q-value FDR B&Y	Hit Count in Query List	Hit Count in Genome
ABCC9	hsa-miR-651-3p	MirDB	2.89E-20	2.40E-16	2.40E-16	2.31E-15	31	1753
ACTB	hsa-miR-377:mirSVR_highEffct	MicroRNA.org	5.71E-16	4.74E-12	2.37E-12	2.28E-11	26	1629
ADAMTSL4	hsa-miR-335-3p	MirDB	9.64E-16	8.00E-12	2.67E-12	2.56E-11	27	1829
AHDC1	hsa-miR-424-5p:Functional MTI	miRTarbase	4.61E-15	3.82E-11	9.56E-12	9.17E-11	16	471
ALX4	hsa-miR-605:mirSVR_highEffct	MicroRNA.org	1.63E-14	1.35E-10	2.71E-11	2.60E-10	21	1100
ANTXR1	hsa-miR-570:PITA	PITA	4.09E-14	3.39E-10	5.65E-11	5.42E-10	20	1015
ASPM	hsa-miR-33b:mirSVR_lowEffct	MicroRNA.org	4.88E-14	4.05E-10	5.75E-11	5.52E-10	26	1972
ASXL1	hsa-miR-411*:mirSVR_lowEffct	MicroRNA.org	6.08E-14	5.05E-10	5.75E-11	5.52E-10	26	1991
ASXL3	hsa-miR-497-5p:Functional MTI	miRTarbase	6.25E-14	5.19E-10	5.75E-11	5.52E-10	15	460
AXIN2	hsa-miR-425:mirSVR_lowEffct	MicroRNA.org	6.93E-14	5.75E-10	5.75E-11	5.52E-10	25	1825
B3GAT3	hsa-miR-1303:mirSVR_lowEffct	MicroRNA.org	9.14E-14	7.59E-10	6.77E-11	6.50E-10	25	1848
BCOR	mmu-miR-6344	MirDB	1.04E-13	8.66E-10	6.77E-11	6.50E-10	20	1068
CASR	hsa-let-7b-5p:Functional MTI	miRTarbase	1.09E-13	9.04E-10	6.77E-11	6.50E-10	21	1214
CBL	hsa-miR-520d-5p	MirDB	1.16E-13	9.63E-10	6.77E-11	6.50E-10	25	1868
CDC45	mmu-miR-290b-5p	MirDB	1.26E-13	1.04E-09	6.77E-11	6.50E-10	21	1223
CEP135	hsa-miR-524-5p	MirDB	1.31E-13	1.08E-09	6.77E-11	6.50E-10	25	1878
CETP	mmu-miR-1192	MirDB	1.60E-13	1.33E-09	7.82E-11	7.51E-10	22	1392
CHD7	hsa-miR-624:mirSVR_lowEffct	MicroRNA.org	3.54E-13	2.94E-09	1.56E-10	1.50E-09	24	1786
CHST3	hsa-miR-548p	MirDB	3.57E-13	2.97E-09	1.56E-10	1.50E-09	21	1292
CNOT2	hsa-miR-3714	MirDB	4.29E-13	3.56E-09	1.78E-10	1.71E-09	19	1011
COLEC10	hsa-miR-15b-5p:Functional MTI	miRTarbase	5.23E-13	4.34E-09	2.07E-10	1.98E-09	17	760
COLEC11	hsa-miR-615-3p:Functional MTI	miRTarbase	5.86E-13	4.86E-09	2.21E-10	2.12E-09	18	893
CPT1A	hsa-miR-548k:mirSVR_highEffct	MicroRNA.org	8.05E-13	6.68E-09	2.87E-10	2.76E-09	23	1680
CRTAP	hsa-miR-20a*:mirSVR_highEffct	MicroRNA.org	8.31E-13	6.90E-09	2.87E-10	2.76E-09	22	1513
CTCF	hsa-miR-1237:PITA	PITA	1.20E-12	9.99E-09	3.82E-10	3.67E-09	11	214
CTNNB1	mmu-miR-12200-5p	MirDB	1.23E-12	1.02E-08	3.82E-10	3.67E-09	21	1379
CTSK	mmu-miR-495-3p	MirDB	1.24E-12	1.03E-08	3.82E-10	3.67E-09	21	1380
CYP17A1	hsa-miR-3125:mirSVR_highEffct	MicroRNA.org	1.39E-12	1.16E-08	4.08E-10	3.92E-09	23	1726
CYP21B	hsa-miR-16-5p:Functional MTI	miRTarbase	1.46E-12	1.21E-08	4.08E-10	3.92E-09	22	1557
CYP26B1	mmu-miR-466i-5p	MirDB	1.48E-12	1.22E-08	4.08E-10	3.92E-09	22	1558
DDX3X	hsa-miR-331-5p:mirSVR_highEffct	MicroRNA.org	2.03E-12	1.68E-08	5.43E-10	5.21E-09	17	828
DIAPH1	hsa-miR-218-2*:mirSVR_lowEffct	MicroRNA.org	2.14E-12	1.78E-08	5.56E-10	5.33E-09	23	1763
DIS3L2	hsa-miR-9-5p	MirDB	2.74E-12	2.27E-08	6.72E-10	6.45E-09	18	980
DMPK	hsa-miR-362-3p:mirSVR_lowEffct	MicroRNA.org	2.75E-12	2.28E-08	6.72E-10	6.45E-09	23	1785
DPH1	mmu-miR-7234-5p	MirDB	3.33E-12	2.76E-08	7.89E-10	7.58E-09	20	1292
EFNA4	mmu-miR-3094-3p	MirDB	3.53E-12	2.93E-08	8.15E-10	7.82E-09	21	1459
EFNB1	hsa-miR-4668-3p	MirDB	4.03E-12	3.35E-08	9.04E-10	8.68E-09	17	865

EFTUD2	hsa-miR-548ah-3p	MirDB	4.90E-12	4.06E-08	1.04E-09	1.00E-08	22	1657
ERCC2	hsa-miR-548am-3p	MirDB	4.90E-12	4.06E-08	1.04E-09	1.00E-08	22	1657
ERF	hsa-miR-548j-3p	MirDB	5.31E-12	4.41E-08	1.08E-09	1.03E-08	22	1664
ESCO2	hsa-miR-548ae-3p	MirDB	5.50E-12	4.56E-08	1.08E-09	1.03E-08	22	1667
FAM20C	hsa-miR-548aq-3p	MirDB	5.50E-12	4.56E-08	1.08E-09	1.03E-08	22	1667
FBN1	hsa-miR-548ar-3p	MirDB	5.60E-12	4.65E-08	1.08E-09	1.03E-08	20	1330
FGF9	hsa-miR-4668-5p	MirDB	5.71E-12	4.74E-08	1.08E-09	1.03E-08	19	1174
FGFR1	hsa-miR-195-5p:Functional MTI	miRTarbase	6.78E-12	5.63E-08	1.25E-09	1.20E-08	15	639
FGFR2	hsa-miR-548e-3p	MirDB	8.08E-12	6.70E-08	1.46E-09	1.40E-08	19	1198
FGFR3	hsa-miR-6838-5p:Functional MTI (Weak)	miRTarbase	8.45E-12	7.01E-08	1.48E-09	1.43E-08	13	431
FGFR4	hsa-miR-548bc	MirDB	8.68E-12	7.20E-08	1.48E-09	1.43E-08	19	1203
FGFRL1	hsa-miR-1283	MirDB	8.76E-12	7.27E-08	1.48E-09	1.43E-08	18	1052
FLNA	hsa-miR-548f-3p	MirDB	9.05E-12	7.51E-08	1.49E-09	1.43E-08	19	1206
FOXP1	hsa-miR-548az-3p	MirDB	9.45E-12	7.84E-08	1.49E-09	1.43E-08	19	1209
FTO	hsa-miR-524-5p:PITA	PITA	9.54E-12	7.92E-08	1.49E-09	1.43E-08	17	914
GINS2	hsa-miR-520d-5p:PITA	PITA	9.54E-12	7.92E-08	1.49E-09	1.43E-08	17	914
GLI3	mmu-miR-669f-3p	MirDB	1.14E-11	9.45E-08	1.71E-09	1.64E-08	20	1384
GLIS3	mmu-miR-467a-3p	MirDB	1.14E-11	9.45E-08	1.71E-09	1.64E-08	20	1384
GNAS	hsa-miR-1245:mirSVR_lowEffct	MicroRNA.org	1.20E-11	9.95E-08	1.71E-09	1.64E-08	20	1388
GNPTAB	mmu-miR-669b-3p	MirDB	1.22E-11	1.01E-07	1.71E-09	1.64E-08	20	1389
GPC3	hsa-miR-548ap-3p	MirDB	1.23E-11	1.02E-07	1.71E-09	1.64E-08	19	1228
HDAC9	hsa-miR-548t-3p	MirDB	1.23E-11	1.02E-07	1.71E-09	1.64E-08	19	1228
HNRNPK	hsa-miR-548aa	MirDB	1.23E-11	1.02E-07	1.71E-09	1.64E-08	19	1228
HUWE1	hsa-miR-6504-3p	MirDB	1.29E-11	1.07E-07	1.76E-09	1.69E-08	17	932
IFT122	hsa-miR-95-5p	MirDB	1.42E-11	1.18E-07	1.88E-09	1.80E-08	20	1401
IFT140	mmu-miR-32-3p	MirDB	1.44E-11	1.20E-07	1.88E-09	1.80E-08	14	557
IFT81	hsa-miR-641:mirSVR_highEffct	MicroRNA.org	1.45E-11	1.20E-07	1.88E-09	1.80E-08	23	1939
IL11RA	hsa-miR-92a-3p:Functional MTI (Weak)	miRTarbase	1.53E-11	1.27E-07	1.95E-09	1.87E-08	20	1407
JAG1	hsa-miR-1264:PITA	PITA	1.57E-11	1.30E-07	1.96E-09	1.88E-08	13	453
KAT6A	hsa-miR-624*:mirSVR_lowEffct	MicroRNA.org	1.58E-11	1.31E-07	1.96E-09	1.88E-08	19	1246
KAT6B	hsa-miR-1277-5p	MirDB	1.62E-11	1.34E-07	1.97E-09	1.90E-08	22	1763
KMT2D	hsa-miR-582-5p:TargetScan	TargetScan	1.75E-11	1.45E-07	2.10E-09	2.02E-08	13	457
KRAS	hsa-miR-200b:mirSVR_highEffct	MicroRNA.org	2.19E-11	1.82E-07	2.57E-09	2.47E-08	22	1791
LHX3	hsa-let-7c-3p	MirDB	2.23E-11	1.85E-07	2.57E-09	2.47E-08	18	1114
LRP5	hsa-miR-4753-3p	MirDB	2.24E-11	1.86E-07	2.57E-09	2.47E-08	19	1272
LTBP1	hsa-miR-217:mirSVR_highEffct	MicroRNA.org	2.31E-11	1.91E-07	2.57E-09	2.47E-08	19	1274
MAGEL2	hsa-miR-561:PITA	PITA	2.33E-11	1.93E-07	2.57E-09	2.47E-08	17	968
MASP1	hsa-miR-4262:mirSVR_highEffct	MicroRNA.org	2.46E-11	2.04E-07	2.57E-09	2.47E-08	22	1802
MBTPS1	hsa-miR-12136	MirDB	2.47E-11	2.05E-07	2.57E-09	2.47E-08	23	1992
MCPH1	hsa-miR-181a-5p:TargetScan	TargetScan	2.48E-11	2.06E-07	2.57E-09	2.47E-08	17	972
MED13L	hsa-miR-181b-5p:TargetScan	TargetScan	2.48E-11	2.06E-07	2.57E-09	2.47E-08	17	972
MEGF8	hsa-miR-181c-5p:TargetScan	TargetScan	2.48E-11	2.06E-07	2.57E-09	2.47E-08	17	972

MSX2	hsa-miR-181d-5p:TargetScan	TargetScan	2.48E-11	2.06E-07	2.57E-09	2.47E-08	17	972
MYADML2	hsa-miR-200a-3p	MirDB	2.63E-11	2.18E-07	2.68E-09	2.57E-08	16	835
NFIA	hsa-miR-7856-5p	MirDB	2.64E-11	2.19E-07	2.68E-09	2.57E-08	17	976
NOTCH2	hsa-miR-3977	MirDB	2.74E-11	2.28E-07	2.74E-09	2.63E-08	13	474
OSTM1	hsa-miR-141-3p	MirDB	2.77E-11	2.30E-07	2.74E-09	2.63E-08	16	838
P4HB	mmu-miR-7229-3p	MirDB	3.19E-11	2.65E-07	3.12E-09	2.99E-08	17	988
POR	hsa-miR-548n:PITA	PITA	4.55E-11	3.78E-07	4.39E-09	4.22E-08	17	1011
PPP1CB	hsa-miR-10399-5p	MirDB	4.72E-11	3.92E-07	4.42E-09	4.24E-08	16	869
PPP2R1A	hsa-miR-548x-3p	MirDB	4.74E-11	3.93E-07	4.42E-09	4.24E-08	22	1865
PTCH1	hsa-miR-548aj-3p	MirDB	4.74E-11	3.93E-07	4.42E-09	4.24E-08	22	1865
PTPRD	hsa-miR-200b-3p:TargetScan	TargetScan	4.97E-11	4.12E-07	4.48E-09	4.30E-08	16	872
RAB23	hsa-miR-200c-3p:TargetScan	TargetScan	4.97E-11	4.12E-07	4.48E-09	4.30E-08	16	872
RAB5IF	hsa-miR-429:TargetScan	TargetScan	4.97E-11	4.12E-07	4.48E-09	4.30E-08	16	872
RECQL4	hsa-miR-570-3p	MirDB	5.24E-11	4.35E-07	4.68E-09	4.49E-08	21	1688
RNU12	hsa-miR-3138:mirSVR_lowEffct	MicroRNA.org	5.57E-11	4.62E-07	4.88E-09	4.68E-08	22	1881
RUNX2	hsa-miR-4672	MirDB	5.58E-11	4.63E-07	4.88E-09	4.68E-08	16	879
SCN4A	mmu-let-7c-2-3p	MirDB	6.16E-11	5.11E-07	5.27E-09	5.06E-08	17	1031
SH3PXD2B	mmu-let-7a-1-3p	MirDB	6.16E-11	5.11E-07	5.27E-09	5.06E-08	17	1031
SHC4	mmu-miR-98-3p	MirDB	6.25E-11	5.18E-07	5.29E-09	5.08E-08	17	1032
SHH	mmu-let-7f-1-3p	MirDB	6.63E-11	5.50E-07	5.50E-09	5.28E-08	17	1036
SHOC2	mmu-let-7b-3p	MirDB	6.63E-11	5.50E-07	5.50E-09	5.28E-08	17	1036
SKI	hsa-miR-3120-3p	MirDB	7.63E-11	6.33E-07	6.27E-09	6.02E-08	16	898
SLC25A24	mmu-miR-466k	MirDB	9.21E-11	7.64E-07	7.49E-09	7.19E-08	20	1558
SMC1A	mmu-miR-466d-5p	MirDB	9.31E-11	7.73E-07	7.50E-09	7.20E-08	20	1559
SPAG17	hsa-miR-382:mirSVR_highEffct	MicroRNA.org	9.44E-11	7.83E-07	7.53E-09	7.23E-08	19	1385
SPRY1	hsa-miR-2052:mirSVR_highEffct	MicroRNA.org	9.74E-11	8.08E-07	7.70E-09	7.39E-08	20	1563
TBX3	hsa-miR-129-5p:mirSVR_highEffct	MicroRNA.org	1.01E-10	8.39E-07	7.80E-09	7.49E-08	21	1750
TCF12	hsa-miR-374b:PITA	PITA	1.02E-10	8.43E-07	7.80E-09	7.49E-08	15	776
TCOF1	hsa-miR-374a:PITA	PITA	1.02E-10	8.43E-07	7.80E-09	7.49E-08	15	776
TCTN3	hsa-miR-23a-3p:TargetScan	TargetScan	1.16E-10	9.59E-07	8.60E-09	8.25E-08	16	924
TGFBR1	hsa-miR-23c:TargetScan	TargetScan	1.16E-10	9.59E-07	8.60E-09	8.25E-08	16	924
TGFBR2	hsa-miR-23b-3p:TargetScan	TargetScan	1.16E-10	9.59E-07	8.60E-09	8.25E-08	16	924
TRAF7	hsa-miR-1305	MirDB	1.16E-10	9.63E-07	8.60E-09	8.25E-08	18	1234
TRIM37	hsa-miR-618:mirSVR_highEffct	MicroRNA.org	1.20E-10	9.97E-07	8.79E-09	8.44E-08	17	1077
TRPM3	hsa-miR-548l:PITA	PITA	1.21E-10	1.00E-06	8.79E-09	8.44E-08	14	655
TSHR	hsa-miR-3120:mirSVR_highEffct	MicroRNA.org	1.24E-10	1.03E-06	8.92E-09	8.56E-08	22	1962
TWIST1	hsa-miR-98-3p	MirDB	1.29E-10	1.07E-06	9.17E-09	8.80E-08	19	1411
WDR19	hsa-let-7f-1-3p	MirDB	1.30E-10	1.08E-06	9.17E-09	8.80E-08	19	1412
WDR35	hsa-let-7b-3p	MirDB	1.30E-10	1.08E-06	9.17E-09	8.80E-08	19	1412
ZEB2	hsa-let-7a-3p	MirDB	1.34E-10	1.11E-06	9.31E-09	8.94E-08	19	1414
ZIC1	hsa-miR-548m:mirSVR_highEffct	MicroRNA.org	1.42E-10	1.18E-06	9.82E-09	9.43E-08	21	1783
ZNF462	hsa-miR-3688-3p	MirDB	1.48E-10	1.23E-06	1.02E-08	9.76E-08	16	940

Table S11. miRNA prediction using nonsyndromic CS genes.

Gene	ID	Source	p-value	q-value Bonferroni	q-value FDR B&H	q-value FDR B&Y	Hit Count in Query List	Hit Count in Genome
ADCK1	hsa-miR-371-5p:mirSVR_highEffct	MicroRNA.org	5.78E-07	2.70E-03	2.00E-03	1.81E-02	7	1013
ALPL	hsa-miR-329:mirSVR_lowEffct	MicroRNA.org	1.49E-06	6.97E-03	2.00E-03	1.81E-02	8	1710
ALX4	hsa-miR-204-5p:Functional MTI	miRTarbase	1.57E-06	7.36E-03	2.00E-03	1.81E-02	5	396
BBS9	hsa-miR-362-3p:mirSVR_lowEffct	MicroRNA.org	2.05E-06	9.59E-03	2.00E-03	1.81E-02	8	1785
BMP2	hsa-miR-379-3p	MirDB	3.82E-06	1.79E-02	2.00E-03	1.81E-02	5	475
BMP7	hsa-miR-411-3p	MirDB	3.86E-06	1.80E-02	2.00E-03	1.81E-02	5	476
BMPER	AAAGGGA,MIR-204:MSigDB	MSigDB	3.91E-06	1.83E-02	2.00E-03	1.81E-02	4	211
CIMIP1	AAAGGGA,MIR-211:MSigDB	MSigDB	3.91E-06	1.83E-02	2.00E-03	1.81E-02	4	211
CTCFL	hsa-miR-31:mirSVR_highEffct	MicroRNA.org	4.13E-06	1.93E-02	2.00E-03	1.81E-02	6	868
DLG1	hsa-miR-625*:mirSVR_lowEffct	MicroRNA.org	4.28E-06	2.00E-02	2.00E-03	1.81E-02	7	1371
FAM209A	hsa-miR-584-5p	MirDB	9.74E-06	4.56E-02	4.14E-03	3.74E-02	4	266
FREM1	mmu-miR-1271-5p	MirDB	1.18E-05	5.52E-02	4.60E-03	4.15E-02	5	600
IGF1R	hsa-miR-548an	MirDB	1.68E-05	7.88E-02	5.36E-03	4.84E-02	6	1112
JAG1	hsa-miR-432-5p	MirDB	1.71E-05	8.00E-02	5.36E-03	4.84E-02	4	307
NELL1	hsa-miR-6808-5p	MirDB	1.72E-05	8.04E-02	5.36E-03	4.84E-02	5	649
NOTCH1	mmu-miR-96-5p	MirDB	2.00E-05	9.37E-02	5.80E-03	5.23E-02	5	670
PCK1	hsa-miR-651-3p	MirDB	2.11E-05	9.85E-02	5.80E-03	5.23E-02	7	1753
PDILT	hsa-miR-613:mirSVR_lowEffct	MicroRNA.org	2.63E-05	1.23E-01	5.82E-03	5.26E-02	7	1815
PTH2R	hsa-miR-34b:mirSVR_highEffct	MicroRNA.org	2.76E-05	1.29E-01	5.82E-03	5.26E-02	6	1214
RAB23	hsa-miR-548ap-3p	MirDB	2.94E-05	1.37E-01	5.82E-03	5.26E-02	6	1228
RBM38	hsa-miR-548t-3p	MirDB	2.94E-05	1.37E-01	5.82E-03	5.26E-02	6	1228
RECQL4	hsa-miR-548aa	MirDB	2.94E-05	1.37E-01	5.82E-03	5.26E-02	6	1228
RTF2	hsa-miR-448:mirSVR_lowEffct	MicroRNA.org	2.95E-05	1.38E-01	5.82E-03	5.26E-02	7	1848
RUNX2	hsa-miR-34a-5p:Functional MTI (Weak)	miRTarbase	3.11E-05	1.46E-01	5.82E-03	5.26E-02	5	735
SH3PXD2B	hsa-miR-10526-3p	MirDB	3.15E-05	1.47E-01	5.82E-03	5.26E-02	4	359
SHC4	hsa-miR-142-5p:PITA	PITA	3.24E-05	1.51E-01	5.82E-03	5.26E-02	5	741
SPO11	hsa-miR-466:mirSVR_highEffct	MicroRNA.org	3.74E-05	1.75E-01	6.16E-03	5.56E-02	7	1918
TGFBR2	hsa-miR-802:mirSVR_highEffct	MicroRNA.org	3.82E-05	1.79E-01	6.16E-03	5.56E-02	7	1924
ZBP1	hsa-miR-182:mirSVR_lowEffct	MicroRNA.org	3.92E-05	1.83E-01	6.16E-03	5.56E-02	7	1932
